# AI-powered transmitted light microscopy for functional analysis of live cells

**DOI:** 10.1038/s41598-019-54961-x

**Published:** 2019-12-05

**Authors:** Dongyoung Kim, Yoohong Min, Jung Min Oh, Yoon-Kyoung Cho

**Affiliations:** 1 Center for Soft and Living Matter, Institute for Basic Science (IBS), Ulsan, 44919 Republic of Korea; 2 Department of Biomedical Engineering, School of Life Sciences, Ulsan National Institute of Science and Technology (UNIST), Ulsan, 44919 Republic of Korea

**Keywords:** Microscopy, Software, Cancer imaging, Cell migration, Imaging the immune system

## Abstract

Transmitted light microscopy can readily visualize the morphology of living cells. Here, we introduce artificial-intelligence-powered transmitted light microscopy (AIM) for subcellular structure identification and labeling-free functional analysis of live cells. AIM provides accurate images of subcellular organelles; allows identification of cellular and functional characteristics (cell type, viability, and maturation stage); and facilitates live cell tracking and multimodality analysis of immune cells in their native form without labeling.

## Introduction

Microscopy imaging experiments constitute essential assays for cell biology research^1^. There are three common aims: (**1**) subcellular structure segmentation^2^, (**2**) cell status determination^3^, and (**3**) analysis of live cell dynamics^4^. Subcellular structure visualizations are typically performed using fluorescence labeling. Cell status characteristics such as their viability, type, and activity can be classified by dyeing representative biomarkers and evaluating their expression levels. However, performance of such analyses on a live cell is challenging. Expression of exogenous proteins through various transfection techniques would allow application of a specific fluorescent tag to target certain subcellular structures and/or biomarkers in live cells. However, several problems are associated with this approach: expression of exogenous proteins can have unexpected side effects, and some cells, especially immune or primary cells, are not transfectable^5,6^. It is notable that all limitations pertain to fluorescence labeling, with the additional restriction of fluorophore/wavelength selection^7,8^.

Digital image processing heavily extends the ability of the optical microscopy. Algorithms such as detection and segmentation allow making measurements and quantifications from the microscopic images^3^. Although, it may fail with many biological sample data due to its innate heterogeneity and complexity^9^. Recent advances in image processing with artificial intelligence (AI) break such limitations. Especially, deep neural network (DNN) explicate microscopy images in classification and segmentation with great performance^10^. For example, a DNN is able to interpret tissue section images and classify diseases on the level of trained experts^11^. Cell segmentation from microscopic images is carried out using DNNs^12,13^. In-silico staining approaches^14–16^ were developed, in which DNNs generate predictions for fluorescent labels from unstained cells. Microscopic object tracking is demonstrated by a DNN^17^.

**Figure 1:**
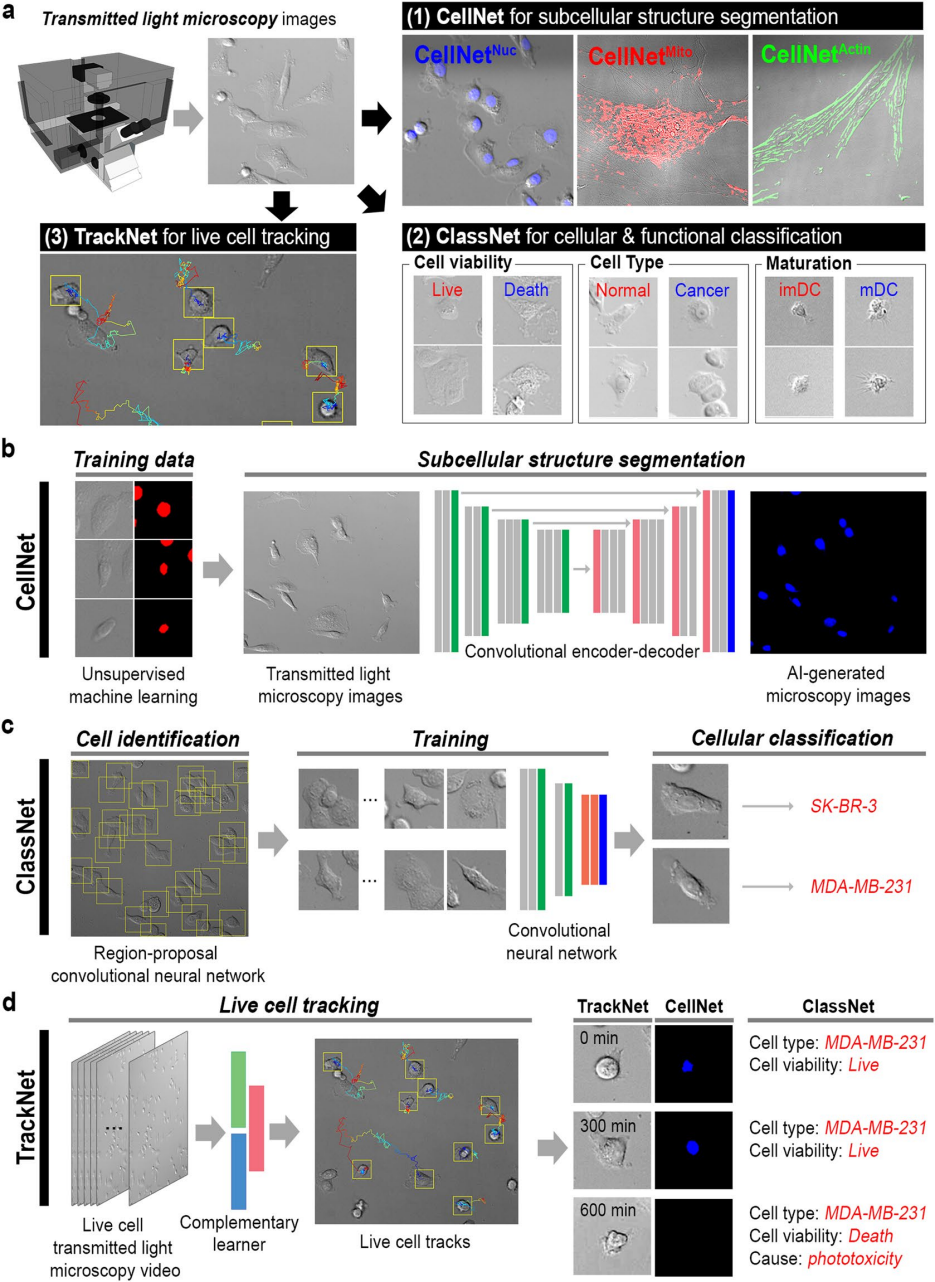
Artificial-intelligence-powered transmitted light microscopy (AIM) with three functional interfaces finds subcellular structures, cellular & functional status information, and cell trajectories from transmitted light microscopy images. (**a**) AIM workflow. Transmitted light images are fed into (**b**) CellNet for subcellular structure segmentation and (**c**) ClassNet for cellular and functional classification, which identifies cell type, viability, conditions, etc. This is extended to live cell tracking and analysis using (**d**) TrackNet (see Methods for details).

Here, we introduce an AI-based software package to perform a complete live cell microscopy data analysis, called AI-powered transmitted light microscopy (AIM, Fig. 1a). Using a set of AI modalities including hierarchical k-means clustering algorithm of unsupervised machine learning, convolutional neural networks in deep learning^18,19^ and a complementary learner solving regression problems of machine learning^20^, AIM performs all three common aims from transmitted light microscopy (TL) images. In this work, we demonstrate (**1**) production of subcellular structure images of cell nuclei, mitochondria, and cytoskeleton fibers using AIM. In addition, (**2**) cellular and functional status information is attained; for example, cell viability, cell type, and immune cell maturation stages are identified. Furthermore, (**3**) accurate live cell tracking with subsequent analysis of the multimodality functions described above is presented, which enables completely label-free and multiplexed live cell imaging.

## Results

The AIM package consists of a hierarchical k-means clustering algorithm of unsupervised machine learning, convolutional neural networks in deep learning and a complementary learner solving regression problems of machine learning. We propose three functional networks: CellNet, ClassNet, and TrackNet (see Methods and Supplementary Notes 1–4). CellNet is engineered to develop images showing subcellular structures from TL images. This is achieved using both unsupervised and supervised machine learning techniques (Fig. 1b and Supplementary Note 2). Intensity clusters are first created from fluorescence microscopy (FL) images using a hierarchical *K*-means clustering algorithm^21^. The intensity cluster and corresponding TL images are fed to a fully convolutional neural network (FCN) that performs pixel-wise classification (see Methods and Supplementary Note 2)^22^. This trains CellNet to create FL-like images from TL images (Fig. 1a). ClassNet is designed for cell location and status classification (Fig. 1a) and is implemented through two convolutional neural networks (CNNs): one region-proposal CNN for cell searching and another CNN for cell classification (Fig. 1c and Supplementary Note 3)^23,24^. Finally, TrackNet is for automated cell tracking. TrackNet exploits an ensemble composed of a correlation filter and a pixel-wise probability to track a live cell (Fig. 1d and Supplementary Note 4)^20^. For each tracking procedure, cellular and functional information are obtained through subsequent analysis with CellNet and ClassNet (Fig. 1d).

**Figure 2:**
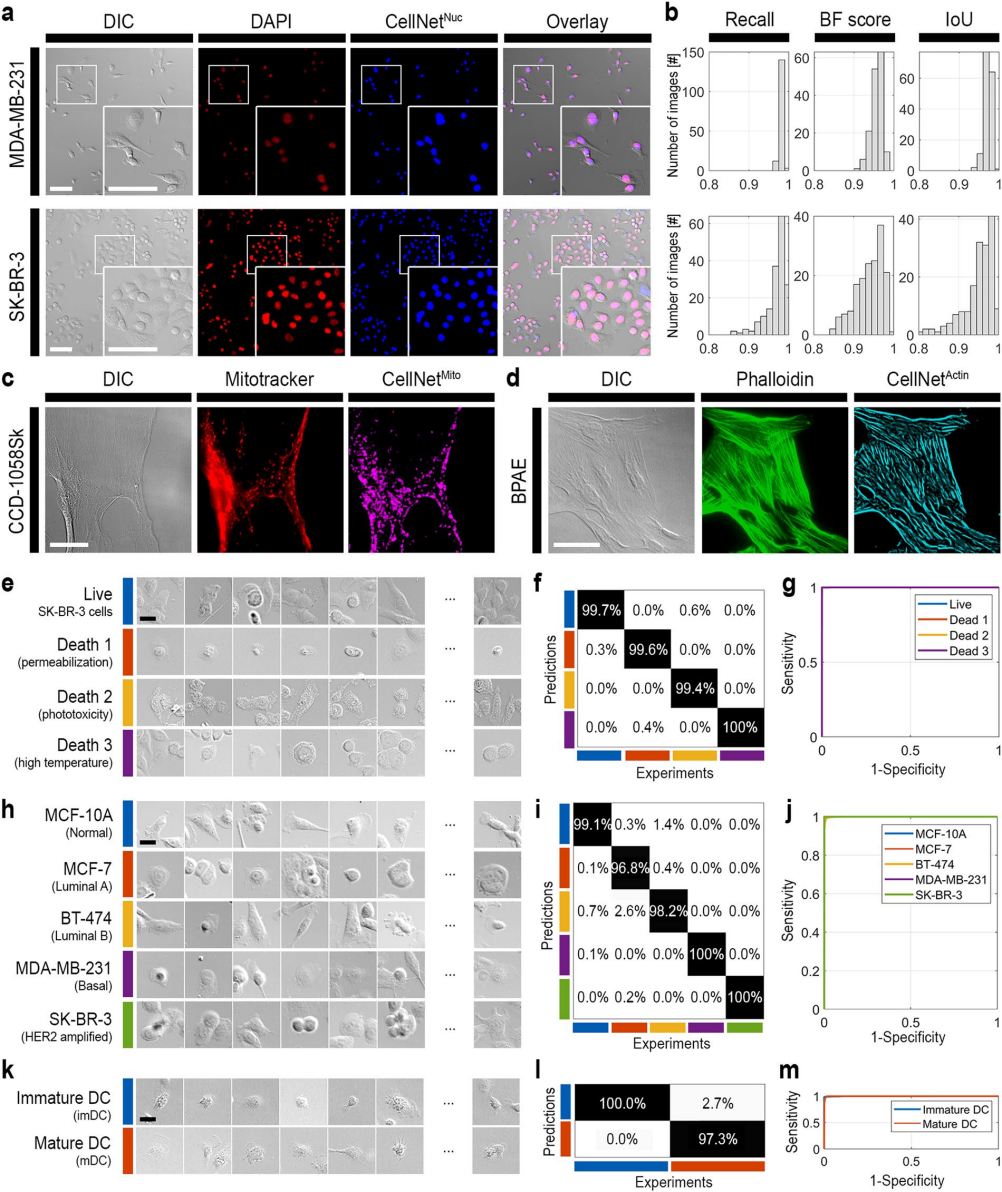
Label-free subcellular structure identification and cellular and functional classification using AIM. (**a**) CellNet^Nuc^ identified cell nuclei from DIC images of MDA-MD-231 and SK-BR-3 cells. (**b**) The CellNet^Nuc^ performance was evaluated based on recall, BF score, and IoU. Demonstrations of (**c**) CellNet^Mito^ and (**d**) CellNet^Actin^ for mitochondrial and actin fiber identification from DIC images, respectively. ClassNet identified the (**e**) cell type, (**h**) cell viability, and (**k**) maturation stage of dendritic cells. The ClassNet classification performance was evaluated using (**f**,**i**,**l**) confusion matrixes and (**g**,**j**,**m**) receiver operating characteristic curves (ROCs) for cell type (**f**,**g**), cell viability (**I**,**j**), and maturation stage identification for dendritic cells (**l**,**m**). Scale bar: (**a**,**c**,**d**) 100 µm, (**e**,**h**,**k**) 20 µm.

CellNet synthesizes FL images from TL images. In this study, subcellular structures (nuclei, mitochondria, and actin fibers) were predicted using this DNN (Fig. 2a–c). From differential interference contrast (DIC) microscopy images of MDA-MB-231 and SK-BR-3 cells, CellNet^Nuc^ generated cell nucleus images, which were comparable to 4′,6-diamidino-2-phenylindole (DAPI)-stained FL images (Fig. 2a). We noticed a few apparent false-positive cells, i.e., cells without DAPI staining that were identified as nuclei by CellNet. In each investigation, we found that most of those cases were in fact true positives with very low DAPI stains (see Supplementary Fig. 1). The CellNet^Nuc^ performance was evaluated according to three criteria: the recall, contour matching score (denoted by the BF score), and intersection-over-union (IoU) score (Fig. 2b, see Methods). CellNet^Nuc^ identified the MDA-MB-231 and SK-BR-3 cell nuclei with more than 98.36% and 96.93% recall and 97.27% and 94.85% IoU, respectively. It also found the respective nucleus boundaries (BF score) with 95.77% and 93.85%. Similarly, CellNet^Mito^ and CellNet^Actin^ were constructed to find mitochondria and actin fibers from TL images, respectively (Fig. 2c,d, Supplementary Note 2). Hence, FL-comparable images could be generated from TL images with 96.88% and 90.12% recall for mitochondria and actin fibers, respectively (Supplementary Fig. 2).

ClassNet was designed to find cell status information, such as details of cell viability, cell type, and dendritic cell maturation stage from TL images alone (Fig. 2e–m). We performed cell viability tests of SK-BR-3 cells with three different causes of death (Fig. 2e, see Methods). A control (live sample) was also considered. The three causes of death were as follows: cell permeabilization (Death 1), phototoxicity (Death 2), and high temperature (Death 3). From the cell images, ClassNet^Viability^ could not only identify the cell viability with 99.78% classification accuracy (Supplementary Fig. 3a), but also classify each death condition with 99.69% classification accuracy (Fig. 2f). The receiver operating characteristic (ROC) curves for ClassNet^Viability^ indicate almost perfect classification performance (Fig. 2g and Supplementary Fig. 3b).

In addition, cancer cell classification was demonstrated using ClassNet^Cancer^ (Fig. 2h–j). Five cell lines from the breast cancer cell panel were used, which were differentiated by molecular signatures (Supplementary Table 1). ClassNet^Cancer^ could distinguish breast cancer cells from normal breast cells with 99.47% classification accuracy (Supplementary Fig. 3c), and differentiate five breast-cancer-cell subtypes with more than 98.80% classification accuracy (Fig. 2i). The ROC curves of both cancer and subtype classifications mark the perfect classification performance of ClassNet^Cancer^ (Supplementary Figs. 2j and 3d).

Dendritic cells (DCs) play a crucial role in adaptive immunity against pathogens and cancer cells via the maturation process^25^. DC maturation involves upregulation of MHC class II, CD40, CD80, and CD86^26^. We successfully demarcated immature DC (imDC) from mature DC (mDC) using ClassNet^DC^ (Fig. 2k–m), again from DIC images obtained without fluorescent labeling. ClassNet^DC^ found the imDC and mDC with 98.63% classification accuracy and the results were well correlated with the CD86 and CD40 expression levels (Fig. 2l and Supplementary Fig. 4). The corresponding ROC curve confirms the good classification performance of ClassNet^DC^ (Fig. 2m).

**Figure 3:**
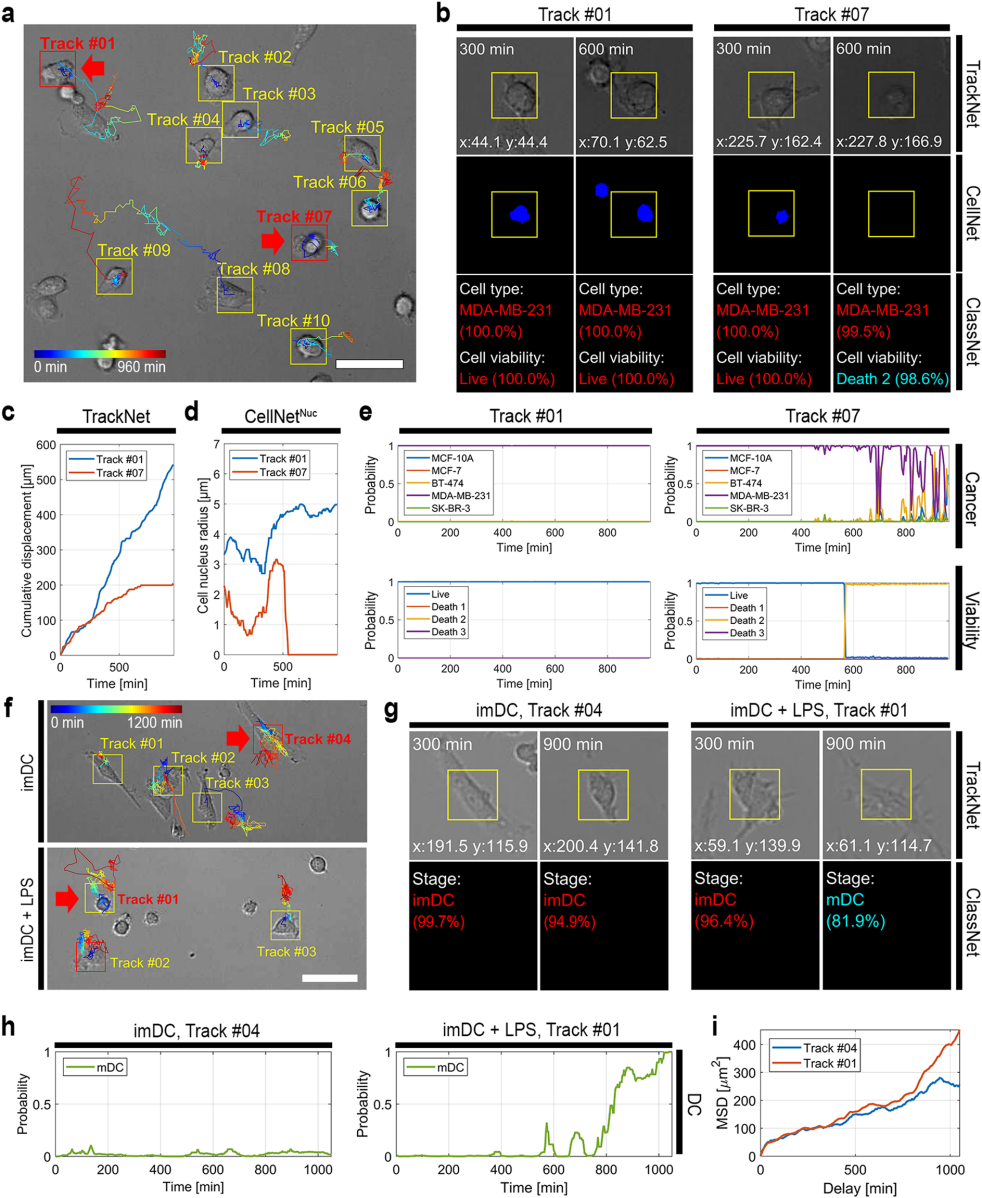
AIM for live cell analysis. (**a**) Example of live cancer cell tracking using TrackNet. (**b**) Label-free, multiplexed live cell analysis performed by combining all three networks: CellNet, ClassNet, and TrackNet. Tracks #01 and #07 of (**a**) were taken as representative examples. (**c–e**) Cumulative trajectory displacements, cell nucleus areas detected using CellNet^Nuc^, and classification probabilities of ClassNet^Cancer^ and ClassNet^Viability^, respectively, for Tracks #01 and #07 plotted over time. (**f**) Live cell tracking and analysis of immature dendritic cells (imDC) for 20 h. The imDCs were in a cell culture medium (**imDC**; top) or a cell culture medium containing lipopolysaccharides (LPS) (**imDC** + **LPS**; bottom). (**g**) Live cell trajectories were obtained for imDC and imDC + LPS using TrackNet (Tracks #04 and #01, respectively) and the maturation stages were identified using ClassNet^DC^. (**h**) The classification probabilities of the DC maturation stage were plotted for Tracks #04 and #01 of (**g**). (**i**) The mean squared displacements (MSDs) were plotted for Tracks #04 and #01 from (**g**). Scale bar: (**a**,**f**) 100 µm.

Label-free, multiplexed live cell tracking and analysis were achieved by combining CellNet, ClassNet, and TrackNet. MDA-MD-231 breast cancer cells were imaged for 16 hours using TL and analyzed using TrackNet (Fig. 3a and Supplementary Video 1–3). Cell images along the trajectory were evaluated by CellNet and ClassNet, which identified the cell nuclei, type, and viability over time (Fig. 3b). Here, we report on two cases: Tracks #01 and #07 of Fig. 3a. Based on the trajectories, the cumulative displacements were plotted over time (Fig. 3c). The Track #01 cell moved continuously to the end of the track whereas the motility of the Track #07 cell decreased. The cell nucleus area was computed for each trajectory (Fig. 3d). No cell nucleus was found after 500 min for Track #07. The cell type (ClassNet^Cancer^) and viability (ClassNet^Viability^) classification probabilities were plotted over time (Fig. 3e). We found that the cell in Track #07 was dead after 500 min, because of the phototoxicity, which was correlated with its motility and the measured cell nucleus size.

We also demonstrated our AIM through application to immune cells. Live imDCs were collected and imaged for 20 h using TL (Fig. 3f–i, Supplementary Videos 4–7). To stimulate DC maturation, lipopolysaccharide (LPS) was added (denoted “imDC + LPS”). The DC trajectories were found using TrackNet and the maturation stages were evaluated using ClassNet^DC^. Two trajectories, i.e., Tracks #04 and #01 for imDC and imDC + LPS, respectively, are discussed as examples here (Fig. 3g). ClassNet^DC^ found that the cell in Track #01 became an mDC after 830 min (Fig. 3g,h, see Supplementary Note 5). The diffusion rate of Track #01 was measured as being higher than that of Track #04 (Fig. 3i). In particular, the mean squared displacement (MSD) increased dramatically after an 830-min delay, implying that the DC diffusive behavior changed after maturation (Fig. 3i; Track #01).

## Discussion

The AIM is an AI toolkit for live cell microscopy from TL images. Three functional AIs, CellNet, ClassNet, and TrackNet, performs cell staining, cell classification and cell tracking accurately (Fig. 1). We were able to produce subcellular structure images of cell nuclei, mitochondria, and cytoskeleton fibers using CellNet (Fig. 2a–d). Identifying cell viability, cell type, and immune cell maturation stage with over 99% classification accuracy was possible with ClassNet (Fig. 2e–m). TrackNet in addition of CellNet and ClassNet performs live cell tracking and analysis (Fig. 3, Supplementary Videos 1–7). The AIM is easy to incorporate to cell biology experiment with existing conventional microscopy setup. The modular architecture of our AIM brings flexibility of experimental design and reusability of each component (Supplementary Notes 1–4). The AIM is an example of using existing AI technologies into a scientific problem. The AI such as SegNet^22^, GoogLeNet^24^, or Staple^20^ were used for self-driving system, image classification, or object tracking problems and were transformed to solve cell staining, disease classification, or living cell tracking in our AIM toolkit. AIM performance is limited by the information available in the TL images. However, this performance could be improved through application of state-of-the-art AI technology. For instance, the spatial resolution of CellNet may be improved using other semantic segmentation techniques^27^ (Supplementary Note 2). In addition, we may construct experiment-exclusive DNNs for ClassNet rather than using networks optimally designed for the ImageNet Large Scale Visual Recognition Competition classification challenge^28^ (Supplementary Note 3, Figs. 5 and 6). Similarly, TrackNet can be improved by using different supervised or unsupervised techniques^17,29^ (Supplementary Note 4). The AIM package developed in this work introduces a new dimension of microscopy and live cell imaging.

## Methods

### AI-powered transmitted light microscopy (AIM)

The artificial-intelligence-powered transmitted light microscopy (AIM) package is constructed from three functional neural networks: CellNet, ClassNet, and TrackNet (Fig. 1). CellNet is designed based on a SegNet^22^ to perform semantic segmentation of subcellular structures. ClassNet is a convolutional neural network (CNN)^23,24^ for cellular and functional classification. TrackNet is constructed based on an ensemble composed of a correlation filter and the intensity histogram approach^20^ and is designed for living cell tracking (see Supplementary Notes 1–4 for details).

CellNet consists of an unsupervised and a supervised machine learning algorithm. Desired features are first identified from fluorescence microscopy images using the hierarchical *K*-means clustering algorithm (HK-means)^21,30,31^. HK-means is an unsupervised machine learning approach^30,31^ and defines intensity classes from fluorescence microscopy images. The results of the unsupervised machine learning are used to supervise CellNet, which is structured using the convolutional encoder-decoder architecture of SegNet^22^ (Fig. 1b and Supplementary Fig. 7). This structure recovers a fine-resolution classification map from a low-resolution encoder feature map. CellNet must be tuned in accordance with the imaging conditions, e.g., the effective pixel size, desired structure dimension, and input image size (Supplementary Fig. 8)^22^. See Supplementary Note 2 for information on data preprocessing and optimization conditions.

ClassNet uses a CNN for cell classification (Fig. 1c and Supplementary Note 3) Two CNN approaches are employed in two steps: cells are found using a region proposal CNN (R-CNN) and the cell status is classified using experiment-specific CNNs. A Faster R-CNN^23^ is used for the cell search. Cell classification is performed by training existing CNNs such as AlexNet^18^, GoogLeNet^24^, Inception-V3^32^, and Inception-ResNet-V2^33^ (Supplementary Note 3 and Supplementary Fig. 5). Note that the performance of these networks differs from the input class (Supplementary Fig. 6). GoogLeNet^24^ was used for the examples presented in the main manuscript (Fig. 2d–l). DNNs were modified according to the input image size and the output class numbers.

TrackNet performs live cell tracking and analysis using CellNet and ClassNet (Fig. 1d). Cells are first found using the R-CNN approach used in ClassNet (Supplementary Fig. 9). For each detection process, live cell tracking is performed by computing the image correlation and intensity histogram in subsequent frames^20^. For each trajectory, the cell images are extracted and analyzed using the pretrained CellNet and ClassNet (Fig. 3, Supplementary Notes 2–4, and Videos 1–7).

All computations reported in this paper were performed using MATLAB (MathWorks, USA) on a personal computer configured with an Intel i7 7700 central processing unit and a single Nvidia GTX 1080 graphics processing unit (GPU). The computations were mostly performed using the GPU. Full details of the network structure used in the manuscript are listed in Supplementary Data 1 to 6.

### Performance evaluation

To evaluate the CellNet performance, the following scores were examined: recall, BF score, and IoU (see Fig. 2b,d)^34^. Recall denotes the ratio of correctly labeled pixels on the following relation:

1 $$Recall=\frac{{\boldsymbol{TP}}}{{\boldsymbol{TP}}+{\boldsymbol{FN}}},$$

where ***TP*** and ***FN*** are the numbers of true positive and false negative pixels, respectively^35^. The boundary F1 contour matching score (BF score) finds the proximity of the boundary at the given error tolerance $\theta =0.75 \%$ of the image diagonal. The precision ***P*** and recall ***R*** are defined as follows:

2 $$\begin{array}{ll}\begin{array}{ccc}{\boldsymbol{P}} & = & \frac{\sum {\boldsymbol{T}}{{\boldsymbol{P}}}_{{\boldsymbol{g}}}}{|{{\boldsymbol{B}}}_{{\boldsymbol{p}}}|},\end{array} & \begin{array}{cc}\begin{array}{ccc}{\boldsymbol{T}}{{\boldsymbol{P}}}_{{\boldsymbol{g}}} & = & \{\begin{array}{c}1,\\ 0,\end{array}\end{array} & \begin{array}{l}\,if\,d({\boldsymbol{x}},{{\boldsymbol{B}}}_{{\boldsymbol{g}}}) < \theta ,x\in {{\boldsymbol{B}}}_{{\boldsymbol{p}}}\\ \,otherwise\,\end{array},\end{array}\\ \begin{array}{ccc}{\boldsymbol{R}} & = & \,\frac{\sum {\boldsymbol{T}}{{\boldsymbol{P}}}_{{\boldsymbol{p}}}}{|{{\boldsymbol{B}}}_{{\boldsymbol{g}}}|},\end{array} & \begin{array}{cc}\begin{array}{ccc}{\boldsymbol{T}}{{\boldsymbol{P}}}_{{\boldsymbol{p}}} & = & \{\begin{array}{c}1,\\ 0,\end{array}\end{array} & \begin{array}{l}if\,d({\boldsymbol{x}},{{\boldsymbol{B}}}_{{\boldsymbol{p}}}) < \theta ,x\in {{\boldsymbol{B}}}_{{\boldsymbol{g}}}\\ otherwise\,\end{array},\end{array}\end{array}$$

where ***B***_*p*_ is the CellNet-image contour map, ***B***_*g*_ is the ground-truth contour map, ***d*** denotes the Euclidean distance, and |***A***| denotes the number of elements in ***A***. The BF score is expressed as follows:

3 $$BF\,score=\,\frac{2\cdot {\boldsymbol{P}}\cdot {\boldsymbol{R}}}{{\boldsymbol{P}}+{\boldsymbol{R}}}.$$

Further, the intersection over union (IoU, or Jaccard similarity coefficient) is scored as

4 $$IoU\,score=\frac{{\boldsymbol{TP}}}{{\boldsymbol{TP}}+{\boldsymbol{FP}}+{\boldsymbol{FN}}},$$

where ***FP*** is the number of false positive pixels.

In this work, the ClassNet performance was evaluated by calculating the confusion matrix^36^ and the receiver operating characteristic (ROC) curve^37^. Classification accuracy is defined:

5 $$Classification\,accuracy=\frac{{\boldsymbol{TP}}+{\boldsymbol{TN}}}{{\boldsymbol{TP}}+{\boldsymbol{FP}}+{\boldsymbol{TN}}+{\boldsymbol{FN}}},$$

where ***TP***, ***FP***, ***TN***, or ***FN*** are the true positive, false positive, true negative, or false negative of the predictions from the ClassNet. Confusion matrixes with absolute data counts are available in Supplementary Table 2. The ROC curves were obtained using the one versus the rest approach^38^. All the performance evaluations were conducted using experimentally independent data sets. Please see Supplementary Note 6 for our comments on this evaluation approach.

### Transmitted light and fluorescence microscopy

The transmitted light and fluorescence microscopy imaging were performed using an inverted microscope (Eclipse-Ti; Nikon, Japan) configured with 20x and 40x dry objective lenses (Plan Apo 20x/0.75NA and 40x/0.95NA, respectively; Nikon, Japan). The transmitted light microscopy was conducted using a differential interference (DIC) contrast setup (Nikon, Japan) configured with white-light light-emitting-diode (LED) illumination (pE-100; CoolLED, UK). For the fluorescence microscopy, the sample was illuminated using colored LED light sources (pE-4000; CoolLED, UK). TRI, RFP, and Cy5 filters (Nikon, Japan) were used, depending on the fluorescence label. The microscopic images were recorded using an electron-multiplying charge-coupled-device camera (iXon Ultra; Andor, UK). A focus stabilization system (PFS; Nikon, Japan) was used for all imaging experiments. All the data were acquired by focusing fiducial markers immediately above the coverslip. The microscope system was controlled using MetaMorph software (Molecular Device, USA).

### Data acquisition and preprocessing for AIM

A motorized stage (Ludl Electronic product, USA) with automated sample scanning capability and a multi-position imaging system (MetaMorph; Molecular Device, USA) were configured for the data acquisition. DIC and nucleus (4′,6-diamidino-2-phenylindole (DAPI) or Hoechst 33342) stained cell images were acquired for all fixed cell imaging experiments. Other fluorescence channel images were obtained in accordance with the experimental conditions. Imaging area were set to 16 mm × 16 mm per sample. The cell nucleus images were segmented using the HK-means algorithm and regions of interest (ROIs) were found by centering the cell nucleus. ROIs were in 101 pixels × 101 pixels (equivalent to 65 μm × 65 μm in 20x magnification, or equivalent to 32.5 μm × 32.5 μm in 40x magnification). 10,000 to 15,000 ROIs were identified per sample. Three samples were prepared per conditions, 20,000 to 30,000 ROIs from two samples were used as training data and 10,000 to 15,000 ROIs from the other sample were used for validation data. Number of training, validation, and testing images used the manuscript is available in Supplementary Table 3.

### Cell lines and reagents

MCF-10A, MCF-7, BT-474, MDA-MB-231, SK-BR-3, and CCD-1058Sk breast cell lines were obtained from the American Type Culture Collection (ATCC) and maintained by following ATCC protocol (Supplementary Table 1). DAPI (Sigma-Aldrich, USA) or Hoechst 33342 (ThermoFisher, USA) were used in accordance with the manufacturer protocol for the cell nucleus staining. A FluoCells^TM^ prepared slide #1 (ThermoFisher, USA) was used for the mitochondrial and actin fiber imaging experiments (Fig. 2c). The dendritic cells were fluorescently labeled using the following antibodies: anti-CD86 fluorescein isothiocyanate (FITC)-conjugated antibody and anti-CD40 phycoerythrin (PE)-conjugated antibody (ThermoFisher, USA). Cells were fixed using 3.4% paraformaldehyde (PFA; Sigma-Aldrich, USA) in phosphate buffered saline (1xPBS; Sigma-Aldrich, USA).

### Cell viability assay

SK-BR-3 cells were plated on glass-bottom dishes (SPL Life Science, South Korea). Cell death conditions, i.e., permeabilization (Death 1, Fig. 2d), phototoxicity (Death 2, Fig. 2g) and high temperature (Death 3, Fig. 2j), were simulated as follows. To synthesize Death 1, SK-BR-3 cells were treated with 0.1% saponin (Sigma Aldrich, USA) for 10 min at room temperature. Death 2 was achieved by irradiating SK-BR-3 cells with ultraviolet light at 27.9 mW/cm^2^ for 1 h (UVO cleaner; Jelight Company, USA). For Death 3, the SK-BR-3 cells were heat shocked for 10 min at 45 °C using a water bath. The cells were stained using a live/dead viability/cytotoxicity kit (ThermoFisher, USA) according to the manufacturer’s protocol. The cells were fixed using 3.4% PFA in 1xPBS for 10 min at room temperature. The cells were stained with DAPI and imaged using the microscope as described above.

### Cell line classification assay

MCF-10A, MCF-7, BT-474, MDA-MB-231, and SK-BR-3 cells were plated on glass-bottom dishes and incubated for one day in the incubator. The cells were then fixed using 3.4% PFA in 1xPBS for 10 min at room temperature. The cells were stained with DAPI and imaged using the microscope as described above.

### Animals

All animal experiments were conducted under protocols approved by the Institutional Animal Care and Use Committee of Ulsan National Institute of Science and Technology (UNISTIACUC-16-13). All animal experiments were conducted in accordance with the National Institutes of Health “Guide for the Care and Use of Laboratory Animals” (The National Academies Press, 8th Ed., 2011). The personnel who performed the experiment had completed the animal research and ethics courses of the Collaborative Institutional Training Initiative (CITI) Program (USA).

### Dendritic cell preparation

The dendritic cells were isolated as described previously^39^. Briefly, the tibias and femurs of BALB/c mice (8–12 weeks of age, females) were used. To isolate bone-marrow-derived dendritic cells (BMDCs), red blood cells were lysed using ammonium-chloride-potassium (ACK) lysis buffer (Gibco, USA). Bone marrow cells were plated on 24-well cell culture plates (1 × 10^6^ cells/ml). The cells were incubated in a culture medium containing RPMI 1640 supplemented with 5% fetal bovine serum, 1% 100x antibiotic-antimycotic solution, 1% 100x 4-(2-hydroxyethyl)-1-piperazineethanesulfonic acid (HEPES) buffer, 0.1% 1000x 2-mercaptoethanol, 1% L-glutamine (all from Gibco, USA) and 20 ng/ml recombinant mouse granulocyte macrophage colony-stimulating factor (GM-CSF) (Peprotech, USA). The culture medium was replaced on days two, four, and six. Non-adhesive and loosely adherent cells were gently collected through a pipette and transferred to Petri dishes. Immature MDCs, which appeared as floating cells, were collected after one day. The BMDCs were validated through flow cytometry assay (Supplementary Note 7 and Figs. 10 and 11).

### Dendritic cell imaging experiment

Glass-bottom dishes were plasma-treated using a plasma cleaner (CUTE-1MPR; Femto Science, South Korea) for 90 s at 100 W. The dishes were then coated with 10 µg/ml fibronectin for one hour at room temperature. BMDCs were plated on the fibronectin-coated dish and incubated for one day in the incubator. To stimulate BMDC maturation, immature BMDCs were treated with 100-ng/ml lipopolysaccharide (LPS; Sigma, USA) for 18 h. The cells were fixed using 3.4% PFA in 1xPBS for 10 min at room temperature. The cells were fluorescently stained and imaged as described above.

### Live cell imaging

The microscope was installed in a cage incubator system (Chamlide HK; Live Cell Instrument, South Korea), which maintained the microscope stage case at 37 °C with 95% humidity and 5% CO_2_ during the experiments. The cancer cells were imaged for 16 h and DIC images were obtained every 5 min (Fig. 3a–e). The dendritic cells were imaged for 20 h and DIC images were obtained every 3 min (Fig. 3f–i).

### Live cell tracking and analysis

Live cell trajectories were found from sets of DIC images using TrackNet (Fig. 3a,f). Along the trajectories, a set of regions of interest (ROIs) was created by centering the track coordinates. The ROIs were analyzed by CellNet and/or ClassNet (Fig. 3b,g). The cumulative displacements (Fig. 3c) or mean squared displacements (MSDs) (Fig. 3h)^40^ were calculated from the live cell trajectories provided by TrackNet. Cell nucleus images were obtained from the ROIs using CellNet (Fig. 3b). The cell nucleus size was measured from the segmented cell nucleus nearest to the track coordinates (Fig. 3d). The classification probabilities were estimated from ClassNet and plotted over time (Fig. 3e,i). See Supplementary Note 8 for living cell analysis pseudo code.

## Supplementary information

Supplementary Information

Supplementary Video 1. Cancer cell tracking demonstration

Supplementary Video 2. Cancer cell tracking demonstration (Track #01)

Supplementary Video 3. Cancer cell tracking demonstration (Track #07)

Supplementary Video 4. Immature dendritic cell tracking demonstration (without lipoplysaccharide (LPS))

Supplementary Video 5. Immature dendritic cell tracking demonstration (without lipoplysaccharide (LPS), Track #04)

Supplementary Video 6. Immature dendritic cell tracking demonstration (with lipoplysaccharide (LPS))

Supplementary Video 7. Immature dendritic cell tracking demonstration (with lipoplysaccharide (LPS), Track #01)

## Data Availability

The data that support the findings of this study are available from the corresponding author upon reasonable request.

## Code availability

All software will be available on GitHub when the manuscript being public. The software used for live cell tracking was originally from Staple^20^.
