# Supplementary material for: AI-powered transmitted light microscopy for functional analysis of live cells
Source: Sci Rep. 2019 Dec 5;9:18428. doi: 10.1038/s41598-019-54961-x (PMC6895055; doi:10.1038/s41598-019-54961-x)
Supplement: Supplementary file 1 — Supplementary Information [file 41598_2019_54961_MOESM1_ESM.docx]

Supplementary Information for

AI-powered transmitted light microscopy for functional analysis of live cells

Dongyoung Kim^1^, Yoohong Min^1^, Jung Min Oh^1,2^, and Yoon-Kyoung Cho^*1,2^

^1^Center for Soft and Living Matter, Institute for Basic Science (IBS), Ulsan 44919, Republic of Korea

^2^Department of Biomedical Engineering, School of Life Sciences, Ulsan National Institute of Science and Technology (UNIST), Ulsan 44919, Republic of Korea

*To whom correspondence should be addressed: E-mail: ykcho@unist.ac.kr

Table of Contents

[Supplementary Notes 4](#_Toc18420300)

[Supplementary Note 1. Concept underlying AI-powered transmitted light microscopy (AIM) 4](#_Toc18420301)

[Supplementary Note 2. CellNet implementation and training 7](#_Toc18420302)

[Supplementary Note 3. ClassNet implementation and training 9](#_Toc18420303)

[Supplementary Note 4. TrackNet implementation 10](#_Toc18420304)

[Supplementary Note 5. ClassNet^DC^ for DC maturation classification 11](#_Toc18420305)

[Supplementary Note 6. CellNet evaluation problem 11](#_Toc18420306)

[Supplementary Note 7. Dendritic cell flow cytometry assay 12](#_Toc18420307)

[Supplementary Note 8. Pseudo code of AIM for living cell analysis. 12](#_Toc18420308)

[Supplementary Figures 14](#_Toc18420309)

[Supplementary Figure 1. CellNet detection of hidden fluorescence signal 14](#_Toc18420310)

[Supplementary Figure 2. Performance evaluation of CellNet^Mito^ and CellNet^Actin^ 15](#_Toc18420311)

[Supplementary Figure 3. Living/dead and cancer cell classifications using ClassNet 16](#_Toc18420312)

[Supplementary Figure 4. ClassNet^DC^ identification and maturation marker expression levels 17](#_Toc18420313)

[Supplementary Figure 5. CNNs tested for ClassNet 18](#_Toc18420314)

[Supplementary Figure 6. ClassNet performance evaluation 20](#_Toc18420315)

[Supplementary Figure 7. CellNet structures 21](#_Toc18420316)

[Supplementary Figure 8. CellNet performance evaluations according to network depth 22](#_Toc18420317)

[Supplementary Figure 9. Cell detection using Faster R-CNN 23](#_Toc18420318)

[Supplementary Figure 10. Dendritic cell maturation analyzed using flow cytometry 24](#_Toc18420319)

[Supplementary Figure 11. Flow cytometry data gating strategy 25](#_Toc18420320)

[Supplementary Tables 26](#_Toc18420321)

[Supplementary Table 1. Cell lines used in study. 26](#_Toc18420322)

[Supplementary Table 2. Confusion matrixes with absolute data count 27](#_Toc18420323)

[Supplementary Table 3. Number of training, validation, and test images used in this study. 29](#_Toc18420324)

[Supplementary Videos 30](#_Toc18420325)

[Supplementary Video 1. Cancer cell tracking demonstration 30](#_Toc18420326)

[Supplementary Video 2. Cancer cell tracking demonstration (Track #01) 31](#_Toc18420327)

[Supplementary Video 3. Cancer cell tracking demonstration (Track #07) 32](#_Toc18420328)

[Supplementary Video 4. Immature dendritic cell tracking demonstration (without lipoplysaccharide (LPS)) 33](#_Toc18420329)

[Supplementary Video 5. Immature dendritic cell tracking demonstration (without lipoplysaccharide (LPS), Track #04) 34](#_Toc18420330)

[Supplementary Video 6. Immature dendritic cell tracking demonstration (with lipoplysaccharide (LPS)) 35](#_Toc18420331)

[Supplementary Video 7. Immature dendritic cell tracking demonstration (with lipoplysaccharide (LPS) , Track #01) 36](#_Toc18420332)

[Supplementary Data 37](#_Toc18420333)

[Supplementary Data 1. CellNet^Nuc^ network details. 37](#_Toc18420334)

[Supplementary Data 2. CellNet^Mito^ network details. 45](#_Toc18420335)

[Supplementary Data 3. CellNet^Actin^ network details. 49](#_Toc18420336)

[Supplementary Data 4. ClassNet^Viability^ network details. 53](#_Toc18420337)

[Supplementary Data 5. ClassNet^Cancer^ network details. 66](#_Toc18420338)

[Supplementary Data 6. ClassNet^DC^ network details. 83](#_Toc18420339)

[Supplementary Data 7. CellNet^Nuc^ convolutional features 100](#_Toc18420340)

[Supplementary Data 8. CellNet^Mito^ convolutional features 103](#_Toc18420341)

[Supplementary Data 9. CellNet^Actin^ convolutional features 105](#_Toc18420342)

[Supplementary Data 10. ClassNet^Viability^ convolutional features 107](#_Toc18420343)

[Supplementary Data 11. ClassNet^Cancer^ convolutional features 115](#_Toc18420344)

[Supplementary Data 12. ClassNet^DC^ convolutional features 123](#_Toc18420345)

[Supplementary Data 13. TrackNet examples 131](#_Toc18420346)

[Supplementary Data 14. AIM examples 137](#_Toc18420347)

[References 147](#_Toc18420348)

# Supplementary Notes

## Supplementary Note 1. Concept underlying AI-powered transmitted light microscopy (AIM)

AI-powered transmitted light microscopy (AIM) is a software package that performs general microscopy image analysis tasks for live cell studies. The aims of such experiments include (**1**) subcellular structure observation, (**2**) cell status determination (e.g., living or dead), and (**3**) live cell dynamics studies. Such microscopy experiments are typically performed using fluorescence microscopy. With highly specific fluorescent labeling or transfection, fluorescence microscopy images provide structural and molecular details of the target specimen, which in turn provides functional status information such as details of cell viability or cancerous activity. As regards live cell tracking, fluorescent labels are preferred for ease of automated segmentation and tracking. Despite its many advantages, however, fluorescence microscopy has intrinsic limitations^1,2^. Firstly, it relies on labeling agents, the signals from which may not be perfectly identical to the native forms of the live cells. For example, well-known problems include transfection efficiency heterogeneity, limited understanding of alien gene activity with a fluorescent protein, and difficulties for specific cell types such as immune and stem cell^3,4^. Secondly, the number of labeling agents that can be used simultaneously is limited for several reasons, including fluorophore availability, absorption/emission spectrum range overlap, and detector sensitivity over wavelength.

There are several studies analyzing medical images using AI. We found two major approaches: firstly, from the immunohistochemistry slides images, many studies are trying to classify the disease by applying deep neural network (DNN)^5–14^. Second, a set of measurements were made from images using traditional image processing approaches or from own methodologies, and then classifying specific subjects using machine learning classifiers^15,16^. There is a label-free microscopy approach with the use of AI, which requires special optics to obtain sophisticated spectral information from the specimen^17^. AI generated image approaches has recently published^18–20^. Cell segmentation from microscopic images is carried out using DNNs^21,22^. This is similar to one of our proposal, CellNet, although CellNet is built on a mixture of unsupervised and supervised AI to minimize human instruction. In addition, here we propose AIM as a complete AI toolkit for living cell analysis. This not only including stained image generation but also experimental classification with absolutely no staining and AI-assisted cell tracking. Plus, it is implemented in a modular architecture which makes the use highly practical.

In this study, use of transmitted light microscopy, e.g., phase contrast (PH) microscopy or differential interference contrast (DIC) microscopy, with the developed AIM is proposed to achieve the above experiment aims (**Fig. 1**). The major hypothesis behind our approach is that fluorescence-microscopy-equivalent information exists in a transmitted light microscopy image. This equivalence can be intuitively explained. If an image of a cell specimen is recorded using transmitted light microscopy, the image visualizes refractive index differences among the cell membrane and organelles in accordance with the cell morphology. These cellular and subcellular signatures are supposed to be genetically imprinted. In the case of cell differentiation, along with complex gene and protein regulations, differentiation occurs with cellular and subcellular structural or morphological changes. For example, cell viability is accompanied by significant cell morphological changes or disappearances through refractive index equilibrium due to permeability increases. Dendritic cell maturation may be accompanied by increasing cell dendrite. Although transmitted light microscopy images contain a variety of information related to cellular and subcellular activities, fluorescence microscopy with specific labeling ability is preferred, as it provides higher-performance. That is, analysis of transmitted light microscopy images through human or traditional image processing techniques is highly inaccurate. Using the power of AI, the AIM package proposed in this study is designed to deliver highly accurate information from transmitted light microscopy images, with the produced information being comparable to that obtained via fluorescence microscopy.

In the **main text** and **Supplementary Notes 2**−**4**, technical implementation of the three functional networks of AIM, i.e., CellNet, ClassNet, and TrackNet, is reported, for subcellular structure segmentation, cell status classification, and live cell tracking, respectively. Individual AI techniques for application in many different fields are already available. For example, the deep neural network (DNN) concept we used for CellNet and ClassNet was developed in 1989^23^. Using such techniques, AIM is designed to obtained functional information from transmitted light microscopy that is of equivalent performance to that from fluorescence microscopy. To achieve higher-performance using AIM, each functional network or component is experimentally miniaturized; this is in contrast to use of a universal predictor. For example, the CellNet implementations reported in the main text (**Figs. 2a**−**2d**) involved three separate pretrained networks, i.e., different CellNets for study of cell nuclei, mitochondria, or actin fibers. Technically, all three can be combined to form a universal subcellular structure segmentation network. However, separate networks were designed to achieve the highest possible performance by reducing the data complexity. Similarly, ClassNets (**Figs. 2e**−**2m**) were also implemented in independent networks for each class. Our design approach gives not only the highest possible performance, but also experimental flexibility without repetitive network training. Addition of a class to an existing DNN typically requires training of the new class with the entire dataset. Techniques such as learning transfer exist, as well as specially designed DNNs for addition of new knowledge to the network^24,25^; however, those approaches sacrifice classification accuracy. In addition, cell biology experiments involve stepwise hypothesis validation and many repetitions, and minimalized networks can be used as the unit operators among such experiments.

The tracking approach employed in TrackNet is from Staple^26^. We housed this unsupervised learning modality, which does not require a training process. A CellNet or ClassNet training set can be experimentally prepared without manual analysis; i.e., no human bias is involved in the analysis. Preparation of training material for tracking, however, requires manual tracking by humans, and is potentially inaccurate and inconsistent. Although a professional may be employed to create training data for tracking, many questions remain regarding the cell location (cell center) and manual tracking accuracy definitions, etc. Based on these considerations, a Staple unsupervised tracking approach is adopted in AIM for further live cell analysis, in addition to CellNet and ClassNet.

## Supplementary Note 2. CellNet implementation and training

CellNet is composed of two different machine learning (ML) approaches: unsupervised and supervised ML (**Fig. 1b**). The training data for the supervised ML are generated from the unsupervised ML. This forms a semi-self-training AI for which the training material for the unsupervised ML is obtained through human instruction; i.e., input of experimentally obtained fluorescence images. In accordance with the target subcellular structure, DIC and fluorescence microscopy images are obtained. The fluorescence microscopy images are processed using the hierarchical *K*-means (HK-means) clustering algorithm; this is the unsupervised ML approach^27,28^. The HK-means processed data contain intensity classifications of the desired subcellular structures. The DIC images and the corresponding HK-means images are fed into a fully convolutional neural network (FCN) for pixel-to-pixel classification^29^. Here, a convolutional encoder-decoder structure of SegNet is employed^30,31^. This SegNet structure with upconvolution layers^4^ helps maintain the resolution of the reconstructed image. However, a series of max-pooling layers induces a loss of spatial resolution^32,33^. In this study, we validated CellNet for different encoder-decoder network depths (**Supplementary Fig. 8**), finding that depth 3 is optimal for nucleus detection under the specific imaging conditions described in the **Methods** (**Supplementary Fig. 7**).

Note that the network structure requires optimization according to the desired subcellular structure and microscopy configuration. In this study, stochastic gradient descent with momentum (SGDM)^34^ was used to train the network in **Supplementary Figs. 7** and **8.** We further fine tune the CellNet using Adam^35^ optimizer to make the best performance at the given data for the data presented in the main figures. The initial learning rate was set to 1e-2 or 5e-4 for SGDM or the Adam optimizer, respectively. The maximum epoch was set to 20. For the Adam optimizer, the gradient decay factor, squared gradient decay factor, and epsilon were set to 0.9, 0.999, and 1e-8, respectively. The training data were shuffled every epoch. Validations were performed during the training using statistically independent data. All the test results reported in the manuscript were obtained from experimentally independent samples. Full details of the CellNet network structure and their convolutional features used in the manuscript are listed in **Supplementary Data 1** to **3** and **7** to **9**. No pretraining nor transfer learning was performed.

The results from the CellNet is limited to the segmentation outcome from HK-means. The HK-means algorithm is generally used for fluorescence micrograph segmentation^36–40^. Although the HK-means algorithm performs the best for fluorescence microscopy image segmentation^36^, the segmentation quality would be highly depending on the image quality and complexity of target structures. Yet, HK-means produces the segmentation of the fluorescence signal at the best of the approach followed by SegNet learning the segmentation, and the inference of the CellNet is the segmentation of the fluorescence signal by the HK-means.

## Supplementary Note 3. ClassNet implementation and training

The ClassNet structure incorporates two convolutional neural networks (CNNs) implemented in two steps: a region proposal CNN (R-CNN) for cell detection and another CNN for cell classification of individual cells (**Fig. 1c**). Cell identifications from DIC images are performed using Faster R-CNN (**Supplementary Fig. 3**)^41^. The training materials are prepared similar to those for CellNet (**Supplementary Note 2**).

In this study, the cell nucleus of the DAPI channel image was segmented using HK-means. The bounding boxes of individual cells were then found by centering each cell nucleus. DIC images with corresponding bounding box coordinates were used to train Faster R-CNN. The network was optimized using SGDM^34^ with an initial learning rate of 1e-3 in 5 epochs. Cell status classification was performed using CNNs. The following CNNs were tested: AlexNet^42^, GoogLeNet^43^, Inception-V3^44^, and Inception-ResNet-V2^45^ (**Supplementary Figs. 5** and **6**). An input image size of 101 × 101 pixels. To avoid the overfitting problem, the input and training data were augmented using 40° random rotation and/or 6-pixel random translation in both the *x* and *y* directions. These augmentations were performed multiple times during training and validation, as necessary. The number of input and validation data elements per class were the same in each experiment. The fully connected layer and the classification layer were defined according to the desired output. We used SGDM^34^ or the Adam^35^ optimizer to train the network in **Supplementary Fig. 6** or **main figures**, respectively. The initial learning rate was set to 1e-3 or 5e-4 for SGDM or the Adam optimizer, respectively. For the latter, the gradient decay factor, squared gradient decay factor, and epsilon were set to 0.9, 0.999, and 1e-6, respectively. Validation was performed every 30 iterations. The maximum epoch was set to 20; however, training was terminated when the maximum number of validations increased. The training data were shuffled every epoch. Note the results from **Supplementary Fig. 6** differs to **Fig. 2** that we gained higher classification accuracies using Adam optimizer.

Using the given data, we tested different CNNs and compared their performance (see **Supplementary Figs. 8** and **9**), finding that GoogLeNet exhibited reliable functionality for all examined cases. All the sample ClassNet implementations discussed in the manuscript were performed using GoogLeNet. The validations during training were conducted using statistically independent data. All the test results shown in the manuscript were obtained from experimentally independent samples. Note that our two-step CNN approach for ClassNet may not be necessary, and could potentially be combined into one R-CNN. However, ClassNet was intentionally constructed using two independent CNNs for the following reasons. Experimentally, the cellular and functional classification problem is independent of the cell finding problem. For a given cell, a number of experiments and classifications can be implemented; i.e., multiplexed analysis can be performed. In other words, cells located using R-CNN can be tested with many CNNs to obtain information. This approach is exemplified in **Figures 2** and **3**. We may create many R-CNNs to perform the same task. However, Faster R-CNN training is more computationally expensive than that for CNNs. Thus, Faster R-CNN is typically implemented using a simple network structure. For instance, a simple two-layered CNN was used in this work. Such a network is not suitable for high-accuracy classification (see **Supplementary Figs. 8** and **9**).

Full details of the ClassNet network structure and their convolutional features used in the manuscript are listed in **Supplementary Data 4** to **6** and **10** to **12**. Neither pretraining nor transfer learning was performed.

## Supplementary Note 4. TrackNet implementation

Automated cell tracking was performed using the Staple approach^26^. In brief, for a given ROI, the correlation score in subsequent frame images and the intensity histogram score were estimated. Both scores were combined to find the cell trajectory through the series of frames (**Figs. 1d** and **3,** and **Supplementary Video** **1**−**7**). This is an unsupervised object tracking approach for which no training is required. As this technique searches for cells in a given area, the cells in subsequent frames should exhibit some continuity. Therefore, determination of a good time resolution for the algorithm depending on cell motility is important. Note we only track the cells from the first frame detected by R-CNN or the cell of interest manually selected for visualization purposes and no incoming cells over time is considered further tracking. There are a number of AI-based object tracking algorithms and a set of techniques specifically for cell tracking have been introduced^46^; those techniques could yield superior performance to the modality employed in this work. **Supplementary Data 13** shows demonstrations of 50 living cell trajectories. In addition, ten more living cell trajectories with CellNet^Nuc^, ClassNet^Viability^, ClassNet^Cancer^, and TrackNet analysis in two imaging conditions, i.e. UV irradiation or control, in **Supplementary Data 14**.

## Supplementary Note 5. ClassNet^DC^ for DC maturation classification

The living dendritic cell maturation in **Figs. 3f−3i** was identified using pretrained ClassNet^DC^ from **Figs.** **2k**−**2m**. ClassNet^DC^ was technically implemented as described in **Supplementary Note 3**. We defined “matured DCs” as immature DCs with 16-h lipopolysaccharide (LPS) treatment. Therefore, the DC maturation definition of ClassNet^DC^ was certainly limited to such DCs regardless of any early maturation stage. A superior DC maturation definition for our AIM implementation could be achieved by using several LPS treatment time points and by training ClassNet to find such a stage.

## Supplementary Note 6. CellNet evaluation problem

CellNet evaluation was performed using the evaluation criteria described in **Methods**. The evaluation required ground truth information which was obtained from the fluorescence microscopy images using HK-means intensity classification (see **Supplementary Note 2**). A problem arose in that there is no guarantee that the “true” ground truth is obtained from the HK-means technique. For example, **Supplementary Figure 1** shows a cell nucleus with a low DAPI signal. The HK-means algorithm missed cell nuclei of this kind, although CellNet identified such cell nuclei correctly, consequently, the evaluation score is lost. This problem could be solved by using superior segmentation approaches or other intensity-based colocalization evaluation methods^47^.

## Supplementary Note 7. Dendritic cell flow cytometry assay

The collected bone-marrow-derived dendritic cells (BMDCs) were cultured in either a cell culture medium or a medium containing 100 ng/mL LPS (Sigma-Aldrich, USA) for 24 h. The BMDCs were then fixed with 2% paraformaldehyde solution in 1xPBS for 10 min at room temperature. The cells were then incubated with the following antibodies^48^: anti-mouse CD11c antibody (allophycocyanin (APC)-conjugated) and anti-mouse CD80 antibody (fluorescein isothiocyanate (FITC)-conjugated, (both from BD-Biosciences, USA), as well as anti-mouse CD86 antibody (phycoerythrin (PE)-conjugated), anti-mouse CD40 antibody (PE-conjugated), and anti-mouse CCR7 antibody (PE-conjugated) (ThermoFisher, USA). The cells with anti-mouse CCR7 antibody were incubated for 30 min at 37 °C, and the remaining antibodies were incubated for 30 min at 4 °C. The fluorescently labeled BMDCs were analyzed using a FACSVerse flow cytometry system (BD Biosciences). Fluorescence compensation beads (ThermoFisher, USA) were prepared following the manufacturer’s protocol. Compensation calibration was performed before every flow cytometry assay by following the protocol in the FACSVerse system using the compensation beads. The acquired flow cytometry data were analyzed using FlowJo (FlowJo, USA) software. The results are shown in **Supplementary Figure 10**. Flow cytometry data gating strategy were exemplified in **Supplementary Figure 11**.

## Supplementary Note 8. Pseudo code of AIM for living cell analysis.

| **Algorithm**: AI Microscopy for living cell analysis $(I,M,D, T)$ |
| --- |
| **Input**:  Transmitted light microscopy images in time $t$, $I = \{I_{1}, I_{2}, \ldots, I_{t}\},$  Pre-trained cellNet or classNet models, $M = \{M_{1}, M_{2}, \ldots M_{k}\}$,  Pre-trained cell detection model $D$,  Object tracking module $T$.  **Output**:  Trajectories of $p$ cells, $S = \{S_{1}, S_{2}, \ldots, S_{p}\}$  Images or classifications from pre-trained cellNet or classNet models along each cell trajectory, $N = \{N_{1,1}, \ldots, N_{k,p}\}$  **Processing**  For $t$, do  From $I_{t}$, $D$ detect $p'$ cells that does not exist in previous timepoint.  For $p'$, do  Track the cell in image sequence $I$ using the object tracking module $T$.  Extract region of interests (ROIs) $R=\{R_{1}^{p},R_{2}^{p},\ldots,R_{t}^{p}\}$ along the trajectory.  Execute pre-trained models $M$ to $R$, and store the results to $N$.  End  End |

# Supplementary Figures


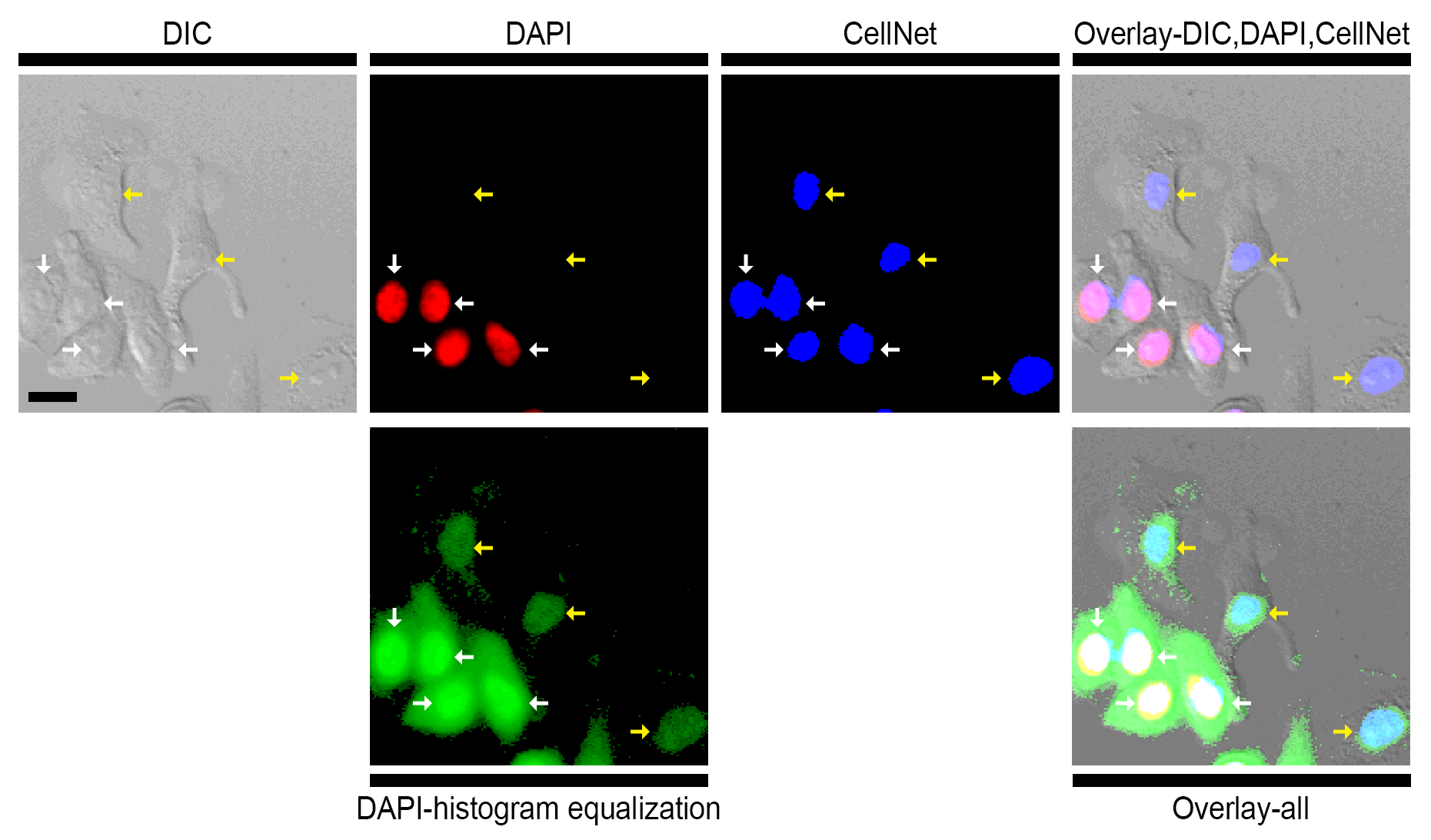


## Supplementary Figure 1. CellNet detection of hidden fluorescence signal

SK-BR-3 cells were stained with DAPI and imaged using DIC and fluorescence microscopy. No DAPI fluorescence signals (red) appeared for the cells with yellow arrows, but CellNet^Nuc^ identified the cell nuclei (blue). The DAPI image processed with histogram equalization shows the cell nuclei with the extremely low fluorescence signals, which are perfectly localized with those in the CellNet^Nuc^ image (blue). Such low fluorescence staining affects the CellNet evaluation (see **Supplementary Note 4**).


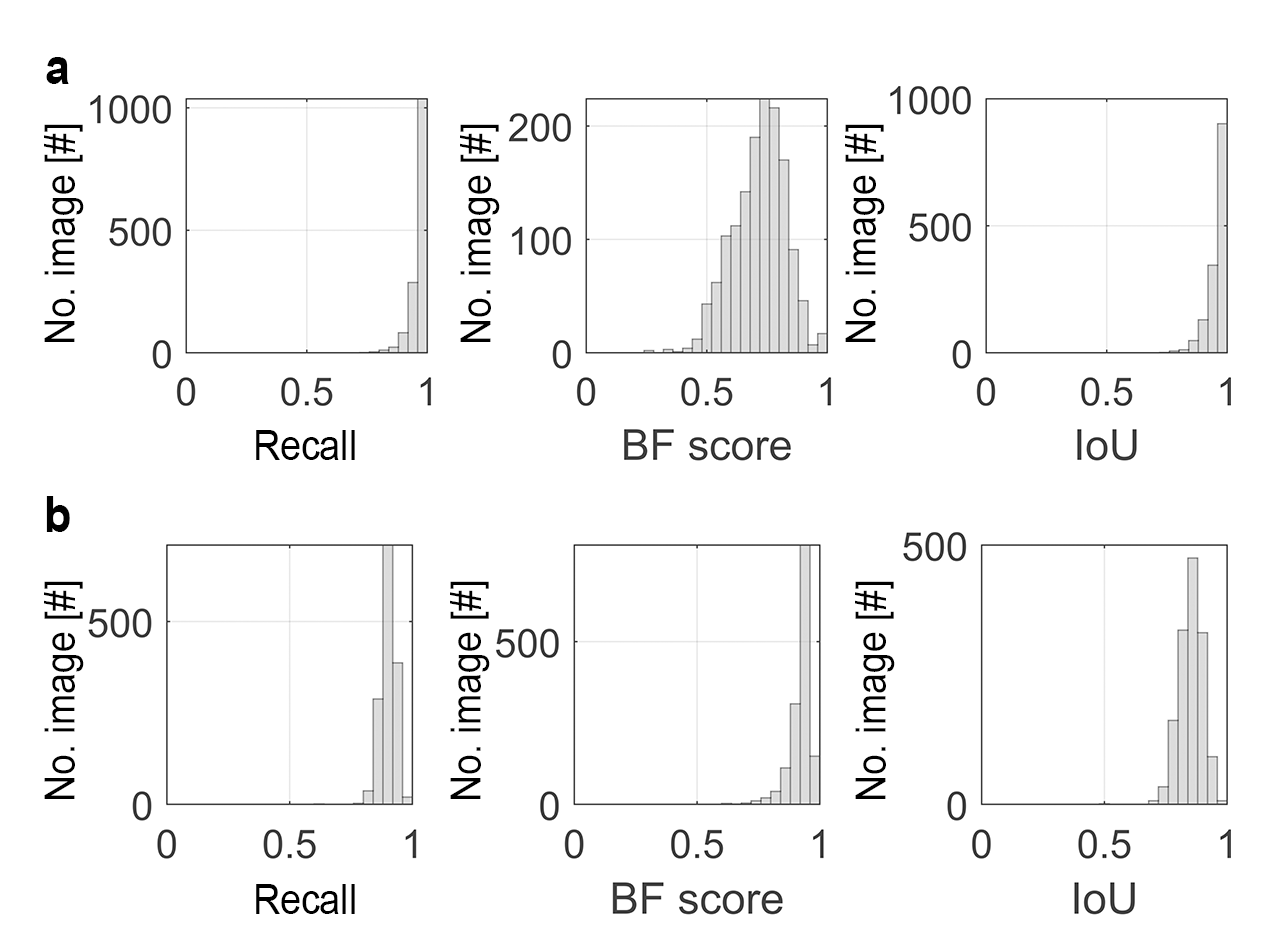


## Supplementary Figure 2. Performance evaluation of CellNet^Mito^ and CellNet^Actin^

The recall, BF score, and IoU were evaluated for (**a**) CellNet^Mito^ and (**b**) CellNet^Actin^ (see **Methods** for the evaluation criteria).


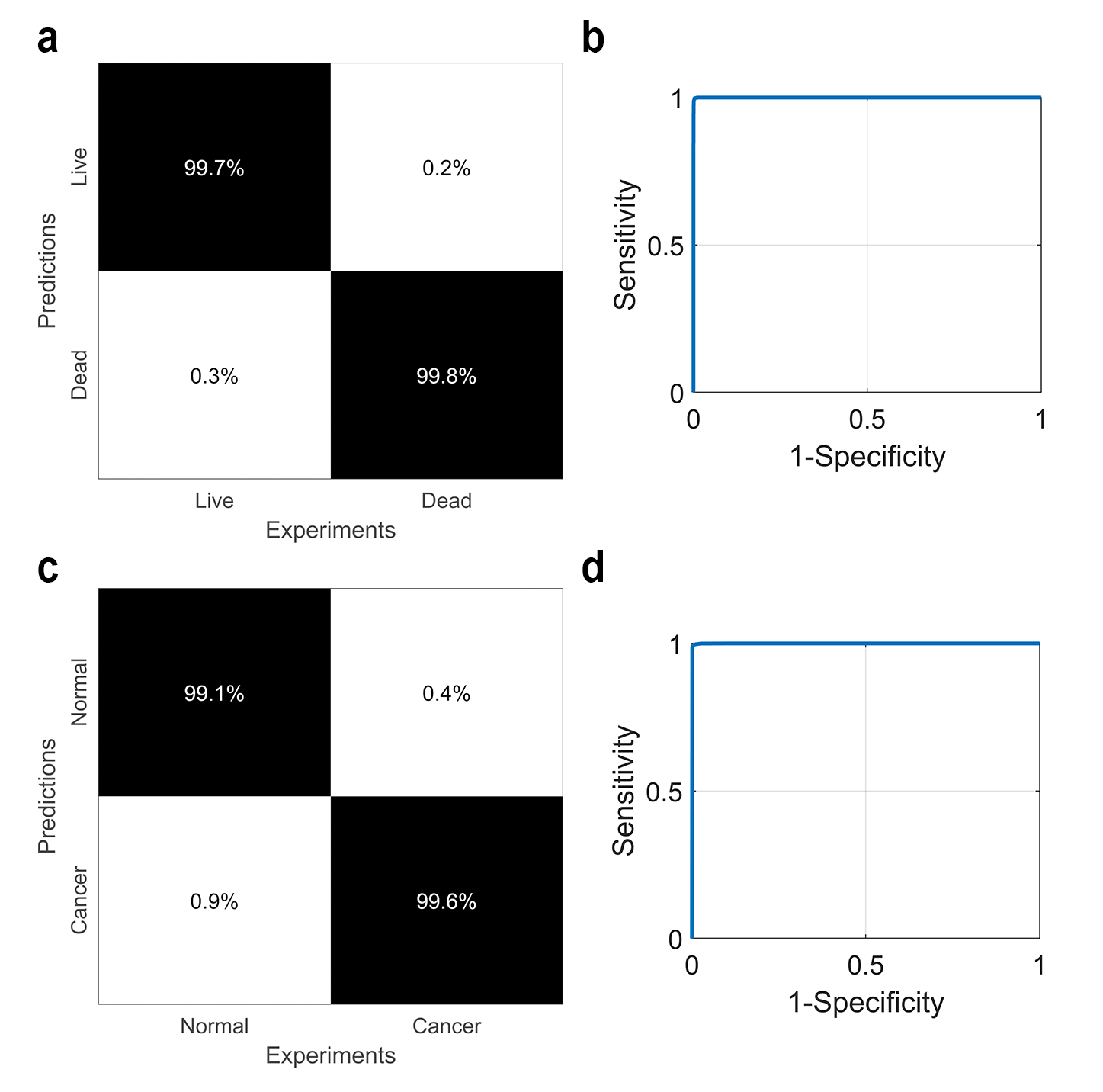


## Supplementary Figure 3. Living/dead and cancer cell classifications using ClassNet

Cell viability and cell lines were identified using ClassNet (**Figs. 2d**−**2i**). The (**a**, **b**) living or dead and (**c**, **d**) cancerous or non-cancerous classification performance was evaluated using the ClassNet implementations reported in the main paper. For both cases, the individual ClassNets could accurately identify each condition with more than 99% classification accuracy (**a**, **c**), with almost perfect classification performance (**b**, **d**; ROC curves).


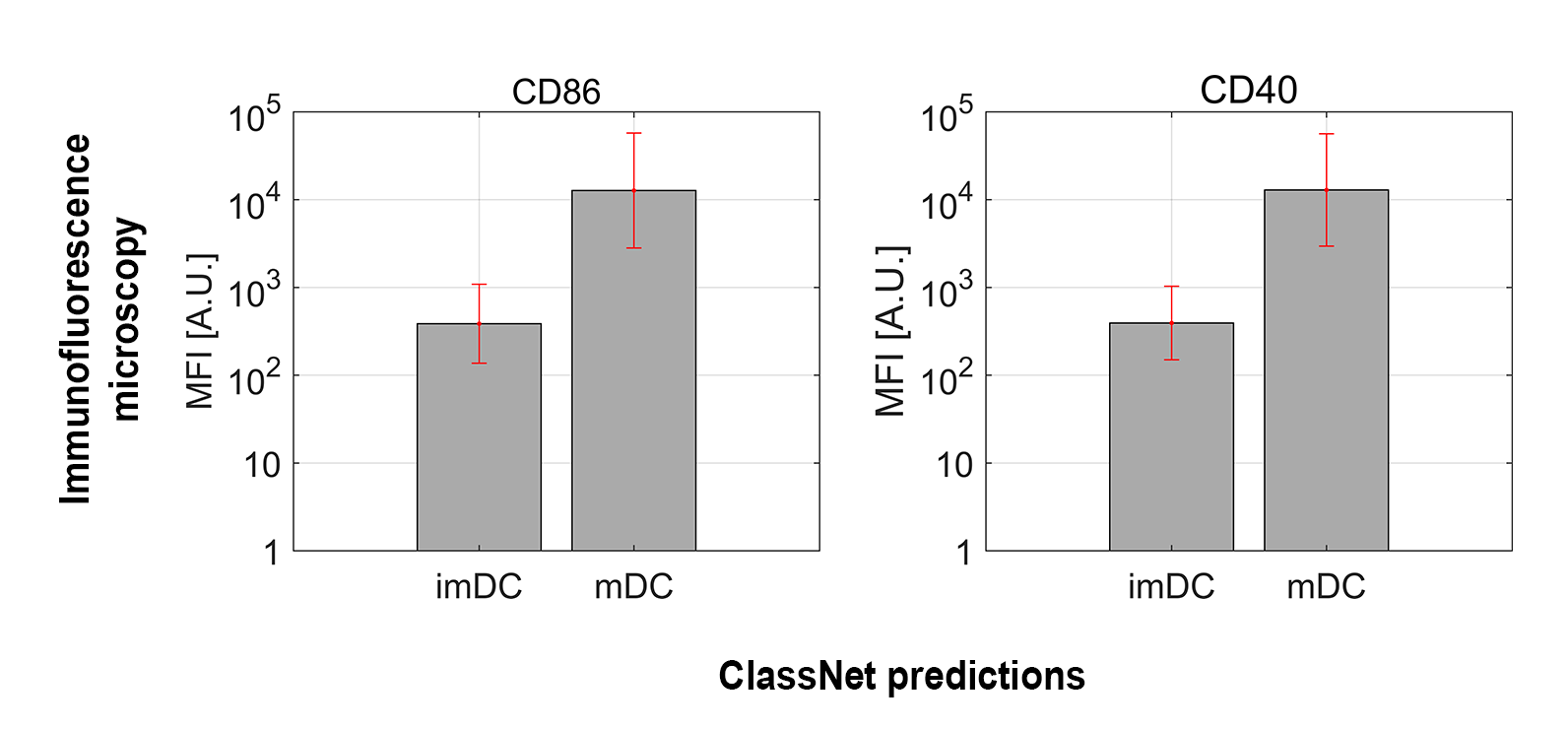


## Supplementary Figure 4. ClassNet^DC^ identification and maturation marker expression levels

DCs were fluorescently labeled with anti-CD86 and anti-CD40 antibodies and imaged using DIC and fluorescence microscopy (see **Methods**). ClassNet^DC^ identified immature DCs (imDCs) and mature DCs (mDCs) from the DIC images. From the corresponding fluorescence microscopy images, the mean fluorescence intensity (MFI) per cell per marker was evaluated. The mDCs classified from ClassNet^DC^ exhibited higher expression of both CD86 and CD40. The same results were obtained in the flow cytometry analysis (see **Supplementary Fig. 10**). The bars and error bars represent the mean and s.d., respectively.


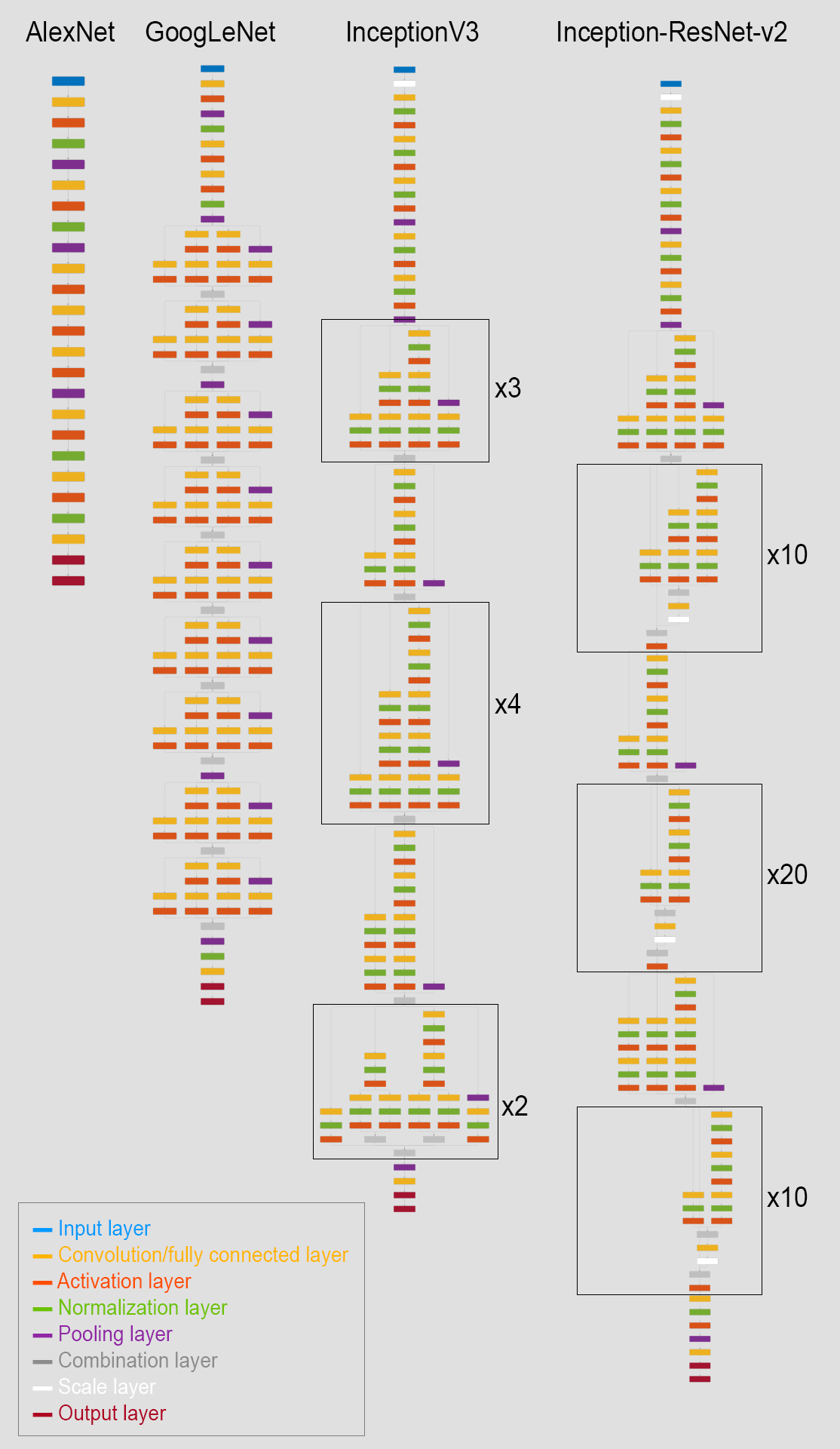


## Supplementary Figure 5. CNNs tested for ClassNet

The following CNNs were tested for ClassNet implementation: AlexNet^42^, GoogLeNet^43^, Inception-V3^44^, and Inception-ResNet-V2^45^. These networks were modified to have a specific data input and output, and no structural modification was added (**Supplementary Note 3**). See **Supplementary Figure 6** for the performance evaluation for these networks. Please find the references for details of the DNN architectures.


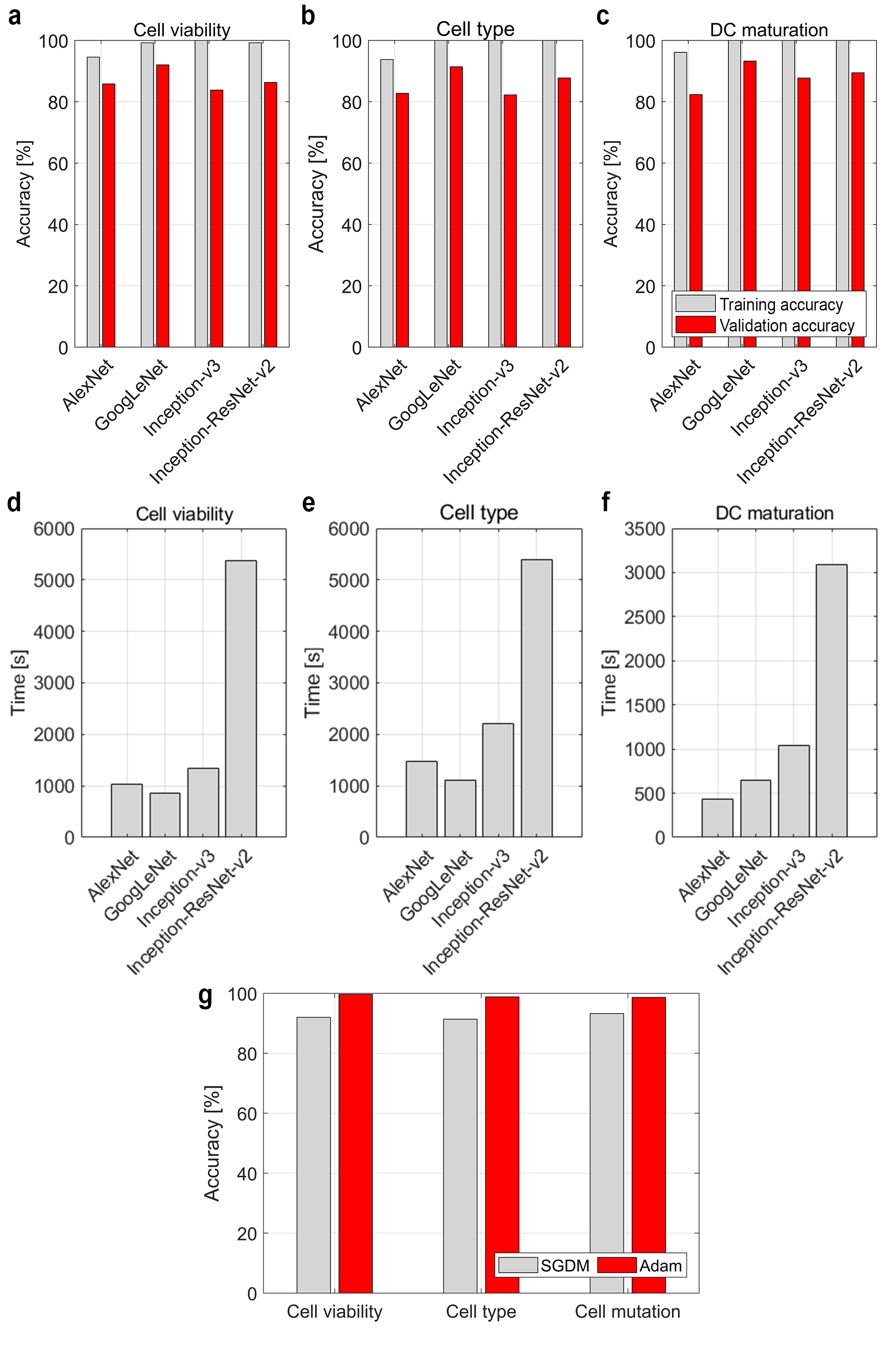


## Supplementary Figure 6. ClassNet performance evaluation

Four CNNs, i.e. AlexNet^42^, GoogLeNet^43^, Inception-V3^44^, and Inception-ResNet-V2^45^ were applied to data from three experiments: (**a**, **d**) cell viability (**Figs. 2e**−**g**), (**b**, **e**) cell type (**Figs. 2h**−**j**), and (**c**, **f**) DC maturation (**Figs. 2k**−**m**). The training accuracy, validation accuracy, and training time were measured for each network. The stochastic gradient descent with momentum (SGDM) optimizer was used for all computations (see **Supplementary Note 3**). Overall, GoogLeNet^43^ yielded the highest validation accuracy with the fastest training performance. (**g**) GoogLeNet can be further optimized using adaptive moment estimation (Adam) which gives better validation accuracy shown in **Fig. 2**.


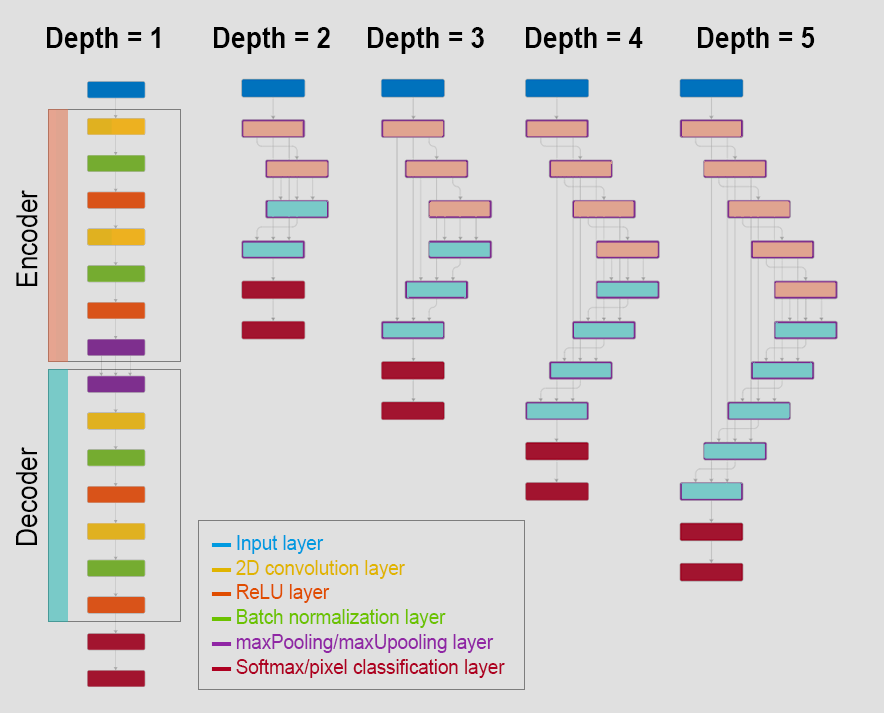


## Supplementary Figure 7. CellNet structures

CellNet follows the SegNet^30^ encoder-decoder structure (see **Supplementary Note 2**). We tested the CellNet performance according to the encoder/decoder depth (depths 1−5). See **Supplementary Figure 8** for the evaluation results.


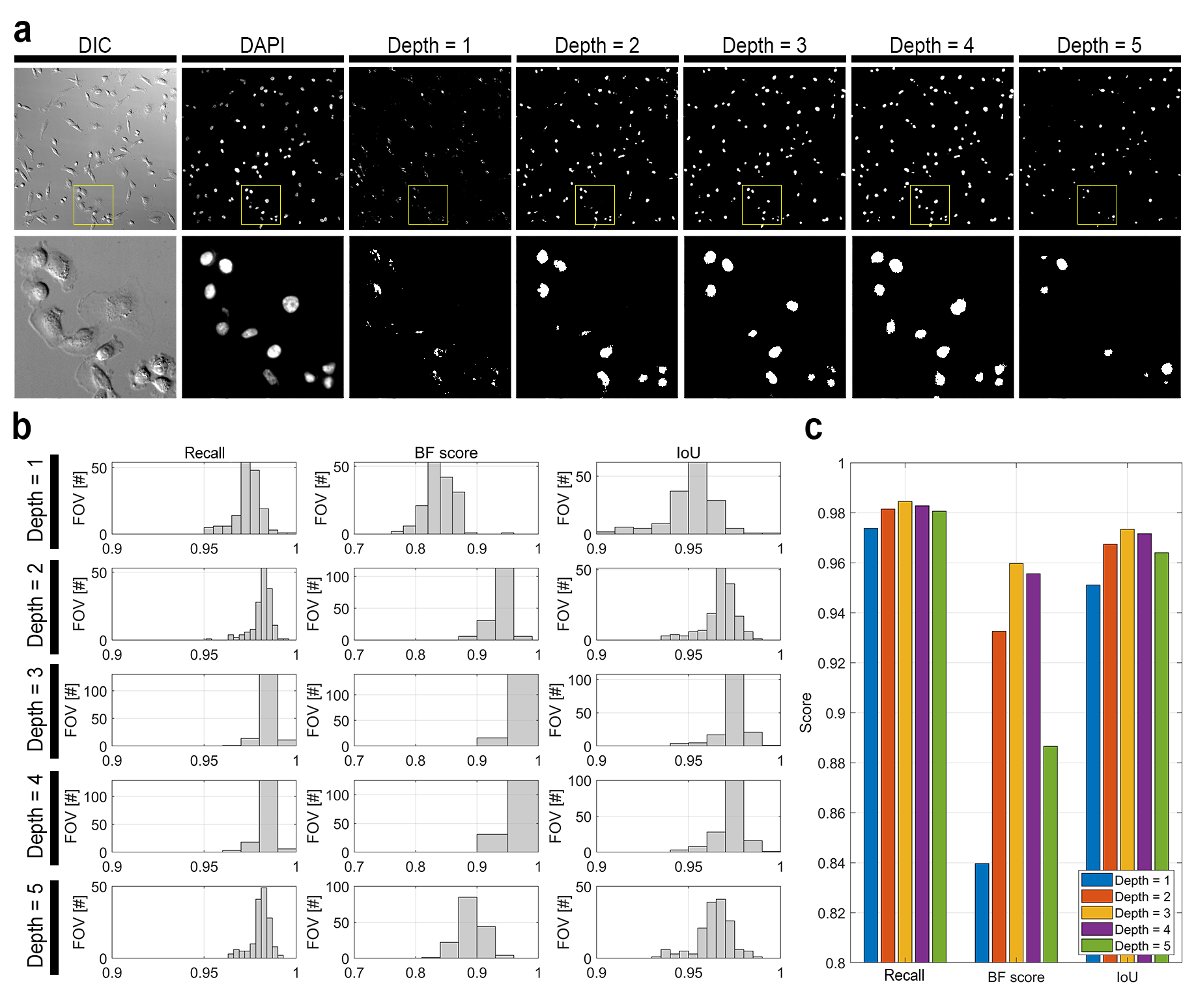


## Supplementary Figure 8. CellNet performance evaluations according to network depth

A CellNet^Nuc^ set was created with different network depths (depths 1−5, see **Supplementary Fig. 7** and **Supplementary Note 2**). MDA-MB-231 cell samples were prepared and imaged using DIC and fluorescence microscopy (see **Methods**). (**a**) CellNet^Nuc^ identification of cell nuclei from DIC images for depths 1−5. (**b**) Performance evaluation of CellNet^Nuc^ with depths 1−5 in terms of recall, BF score, and IoU (see **Methods**) according to field of view (FOV). (**c**) Plot of average scores in (**b**).


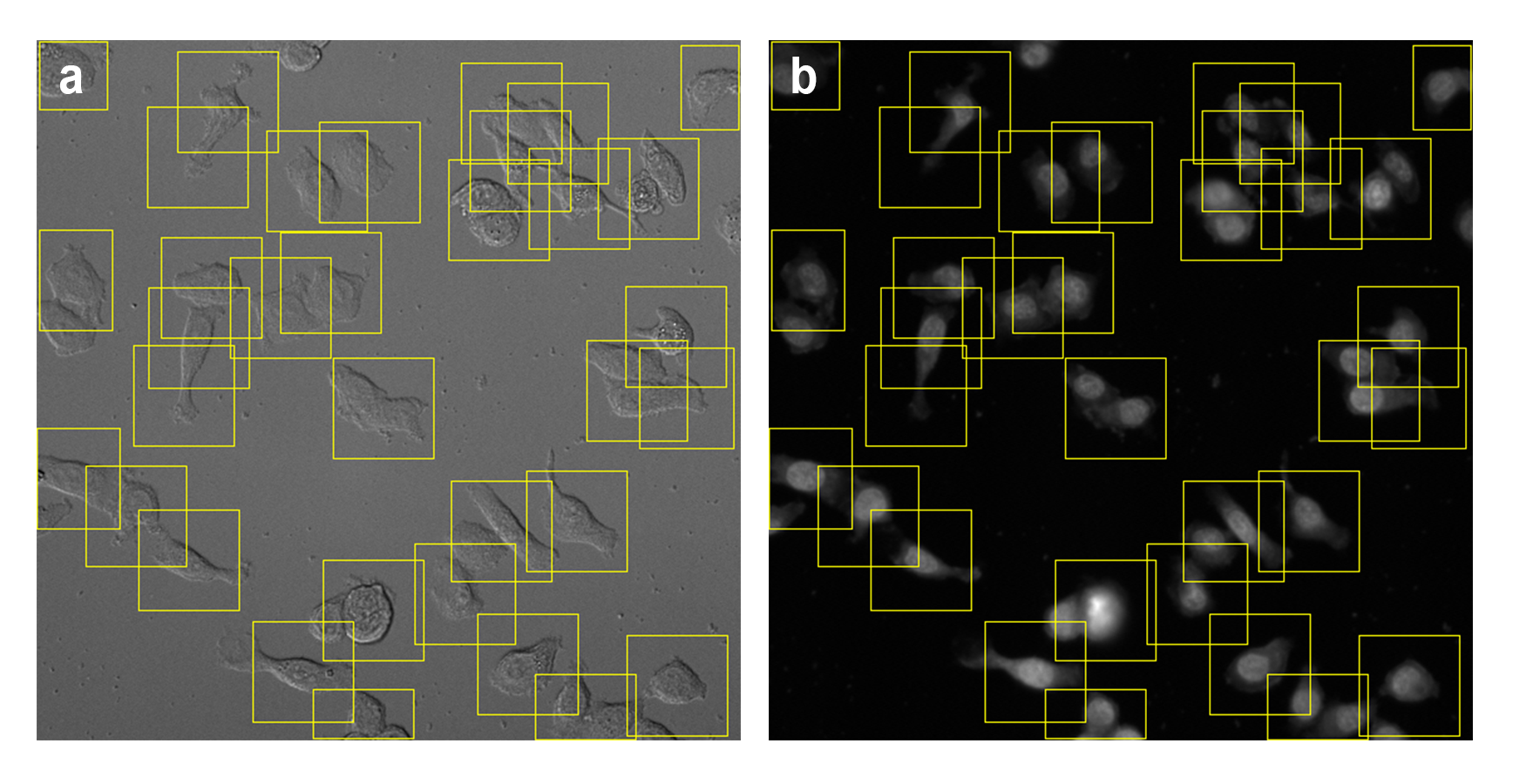


## Supplementary Figure 9. Cell detection using Faster R-CNN

Faster R-CNN^49^ was trained for cell detection (**Supplementary Note 3**). (**a**) SK-BR-3 cells detected using Faster R-CNN from DIC image (yellow bounding boxes) and (**b**) corresponding DAPI fluorescence microscopy image.


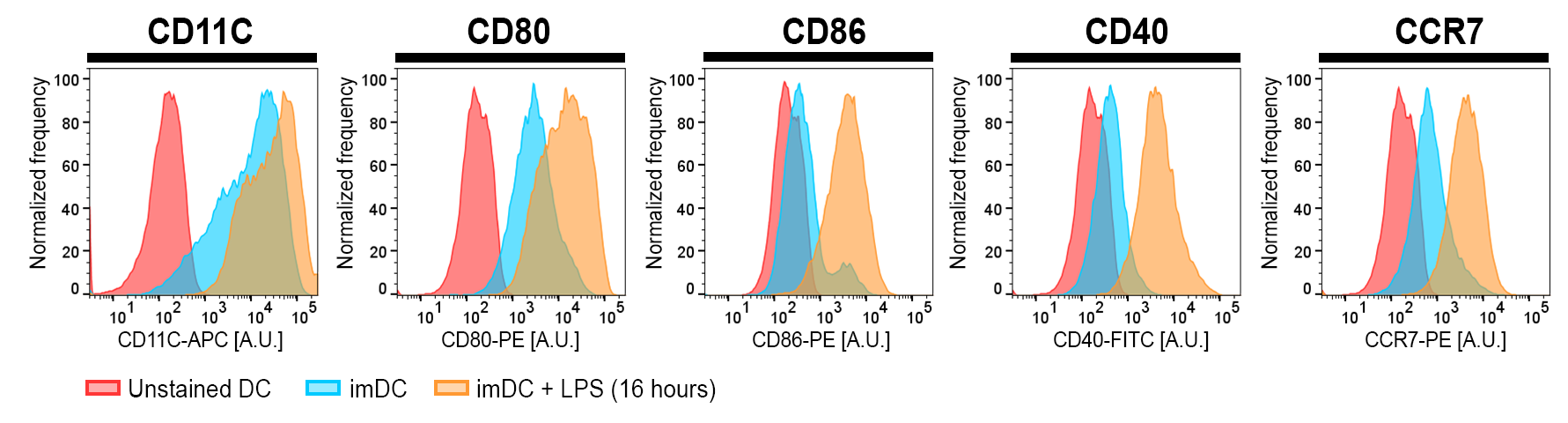


## Supplementary Figure 10. Dendritic cell maturation analyzed using flow cytometry

The following surface protein expression levels were evaluated using flow cytometry, for immature DCs and DCs incubated with LPS for 16 h: CD11c. CD80, CD86, CD40, and CCR7. See **Supplementary Note 7** for details of the flow cytometry assay. The fluorescence intensities were normalized to the median. Here, the red, blue, and orange histogram plots denote unstained DC (negative control), immature DC, and LPS-treated DC, respectively.


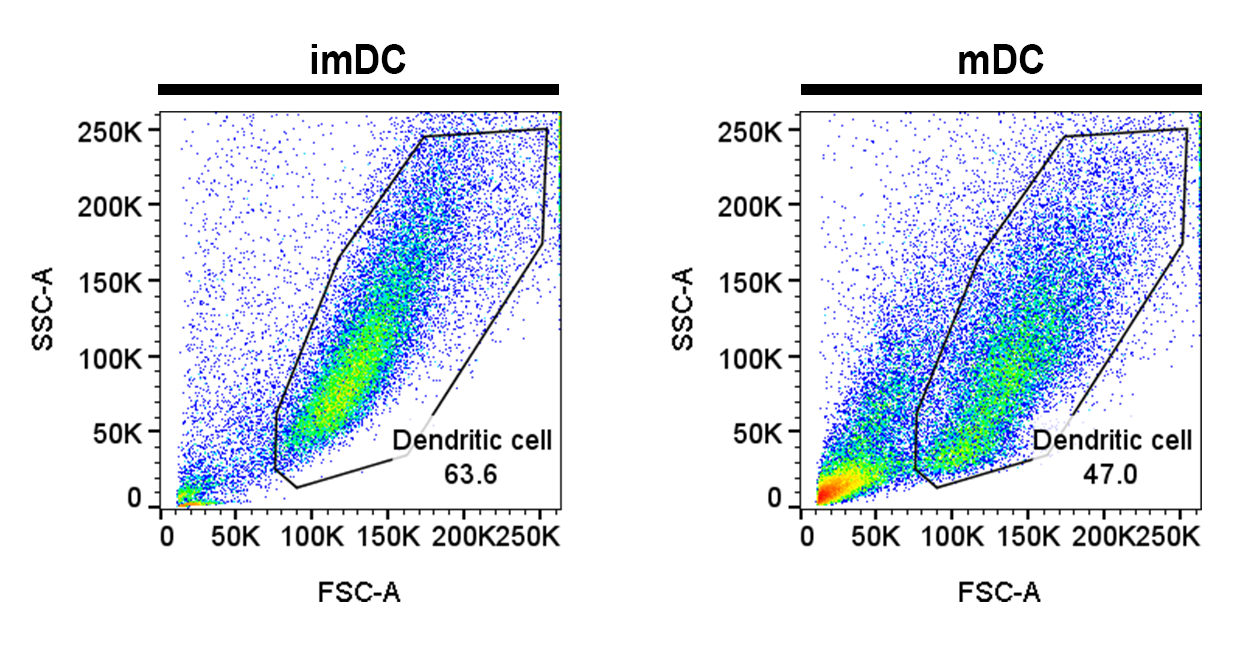


## Supplementary Figure 11. Flow cytometry data gating strategy

Immature and mature dendritic cells (imDCs and mDCs) were analyzed using flow cytometry assay (**Supplementary Note 7**). The cells were obtained by gating data in forward-scattered light (FCS) and side-scattered light (SSC) as described in the figure.

# Supplementary Tables

## Supplementary Table 1. Cell lines used in study.

| **Cell line** | **Description** |
| --- | --- |
| MCF-10A | Normal breast cell line. ER -, PR -, HER2 -. |
| MCF-7 | Breast cancer cell line. Subtype: Luminal A, ER +, PR +/-, HER2 -. |
| BT-474 | Breast cancer cell line. Subtype: Luminal B, ER +, PR +/-, HER2 +. |
| MDA-MB-231 | Breast cancer cell line. Subtype: Basal (triple negative), ER -, PR -, HER2 -. |
| SK-BR-3 | Breast cancer cell line. Subtype: HER2 amplified, ER -, PR -, HER2 +. |

## Supplementary Table 2. Confusion matrixes with absolute data count

The confusion matrixes with the absolute numbers of the image count as well as the percentages used in **Fig. 2** and **Supplementary Fig. 3** are shown below.

|  | **Live** | **Death 1** | **Death 2** | **Death 3** |
| --- | --- | --- | --- | --- |
| **Live** | **99.7%** | **0.0%** | **0.6%** | **0.0%** |
|  | 27039 | 0 | 171 | 0 |
| **Death 1** | **0.3%** | **99.6%** | **0.0%** | **0.0%** |
|  | 70 | 27004 | 0 | 0 |
| **Death 2** | **0.0%** | **0.0%** | **99.4%** | **0.0%** |
|  | 0 | 0 | 26938 | 0 |
| **Death 3** | **0.0%** | **0.4%** | **0.0%** | **100.0%** |
|  | 0 | 105 | 0 | 27109 |

|  | **MCF-10A** | **MCF-7** | **BT-474** | **MDA-MB-231** | **SK-BR-3** |
| --- | --- | --- | --- | --- | --- |
| **MCF-10A** | **99.1%** | **0.3%** | **1.4%** | **0.0%** | **0.0%** |
|  | 11555 | 37 | 166 | 0 | 0 |
| **MCF-7** | **0.1%** | **96.8%** | **0.4%** | **0.0%** | **0.0%** |
|  | 12 | 11288 | 41 | 0 | 0 |
| **BT-474** | **0.7%** | **2.6%** | **98.2%** | **0.0%** | **0.0%** |
|  | 77 | 308 | 11453 | 0 | 0 |
| **MDA-MB-231** | **0.1%** | **0.0%** | **0.0%** | **100.0%** | **0.0%** |
|  | 16 | 0 | 0 | 11660 | 0 |
| **SK-BR-3** | **0.0%** | **0.2%** | **0.0%** | **0.0%** | **100.0%** |
|  | 0 | 26 | 0 | 0 | 11660 |

|  | **imDC** | **mDC** |
| --- | --- | --- |
| **imDC** | **100.0%** | **2.7%** |
|  | 14881 | 398 |
| **mDC** | **0.0%** | **97.3%** |
|  | 0 | 14483 |

|  | **Live** | **Death*** |
| --- | --- | --- |
| **Live** | **99.70%** | **0.20%** |
|  | 27039 | 171 |
| **Death*** | **0.30%** | **99.80%** |
|  | 70 | 81156 |

* “Death” includes cell counts of “Death 1”, “Death 2”, and “Death 3” from **Fig. 2**.

|  | **Normal*** | **Cancer**** |
| --- | --- | --- |
| **Normal*** | **99.10%** | **0.40%** |
|  | 11555 | 203 |
| **Cancer**** | **0.90%** | **99.60%** |
|  | 105 | 46436 |

* “Normal” denotes cell counts from MDF-10A cell line.

** “Cancer” includes cell counts of “MCF-7”, “BT-474”, “MDA-MB-231”, and “SK-BR-3” from **Fig. 2**.

## Supplementary Table 3. Number of training, validation, and test images used in this study.

Training and validation data were spared to 8:2. Additional data argumentation was carried out using “augmentedImageDatastore” function in MATLAB with random rotations and reflections. Training+validation and test images are obtained from independent experiments in same experimental condition.

| Name | Training+Validation | Training | Validation | Testing |
| --- | --- | --- | --- | --- |
| ClassNet^Viability^ | 113256 | 90605 | 22651 | 108436 |
| ClassNet^Cancer^ | 107770 | 86216 | 21554 | 58300 |
| ClassNet^DC^ | 32422 | 25938 | 6484 | 29762 |
| CellNet^Nuc^ | 50185 | 40148 | 10037 | 25106 |
| CellNet^Mito^ | 151600 | 121280 | 30320 | 37900 |
| CellNet^Actin^ | 151600 | 121280 | 30320 | 37900 |

# Supplementary Videos


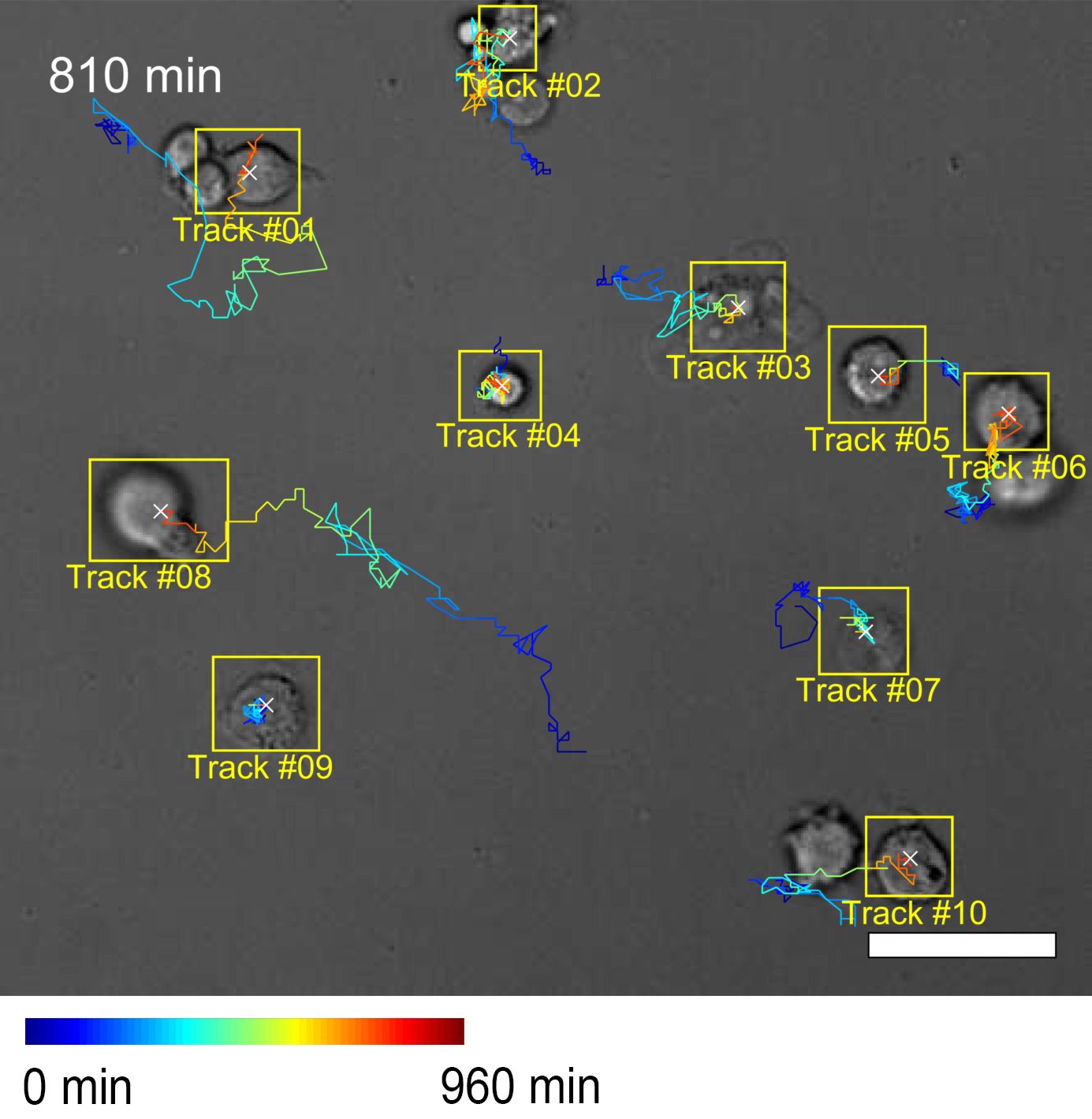


## Supplementary Video 1. Cancer cell tracking demonstration

MDA-MB-231 cells were imaged for 16 h (see **Fig. 3** and **Methods**). The cell trajectories were found using TrackNet (**Methods** and **Supplementary Note 3**). Scale bar: 100 µm.


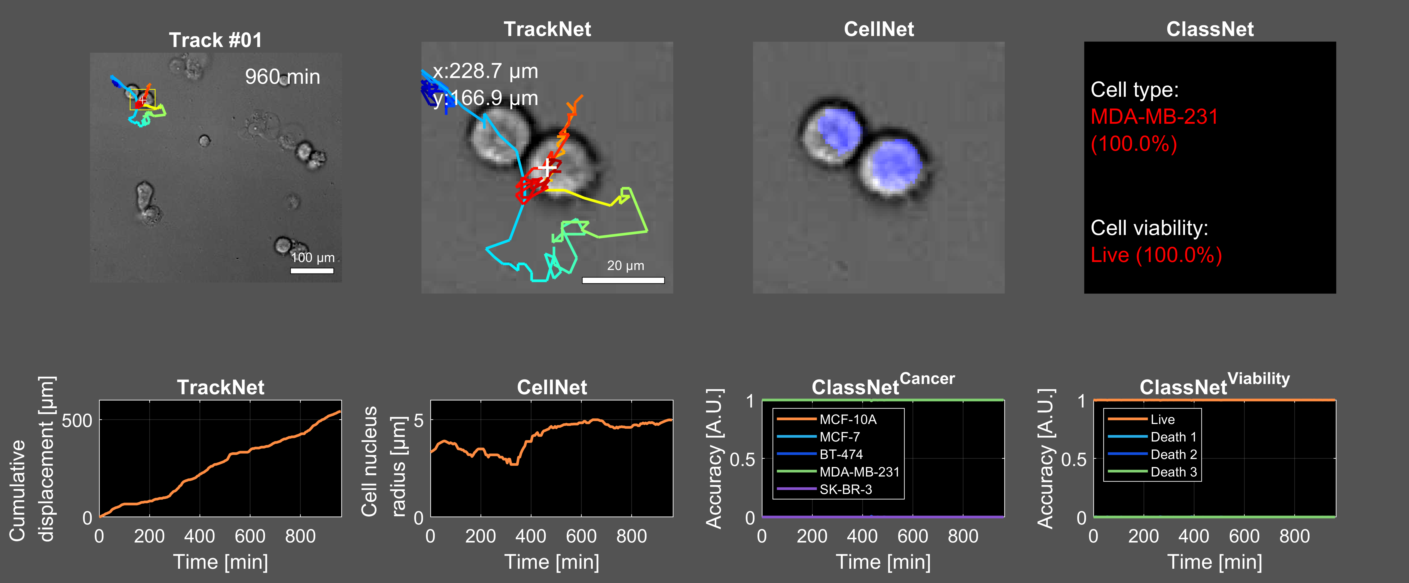


## Supplementary Video 2. Cancer cell tracking demonstration (Track #01)

Track #01 from **Supplementary Video 1** is shown in detail using AIM (**Fig.** **3a**−**3e**).


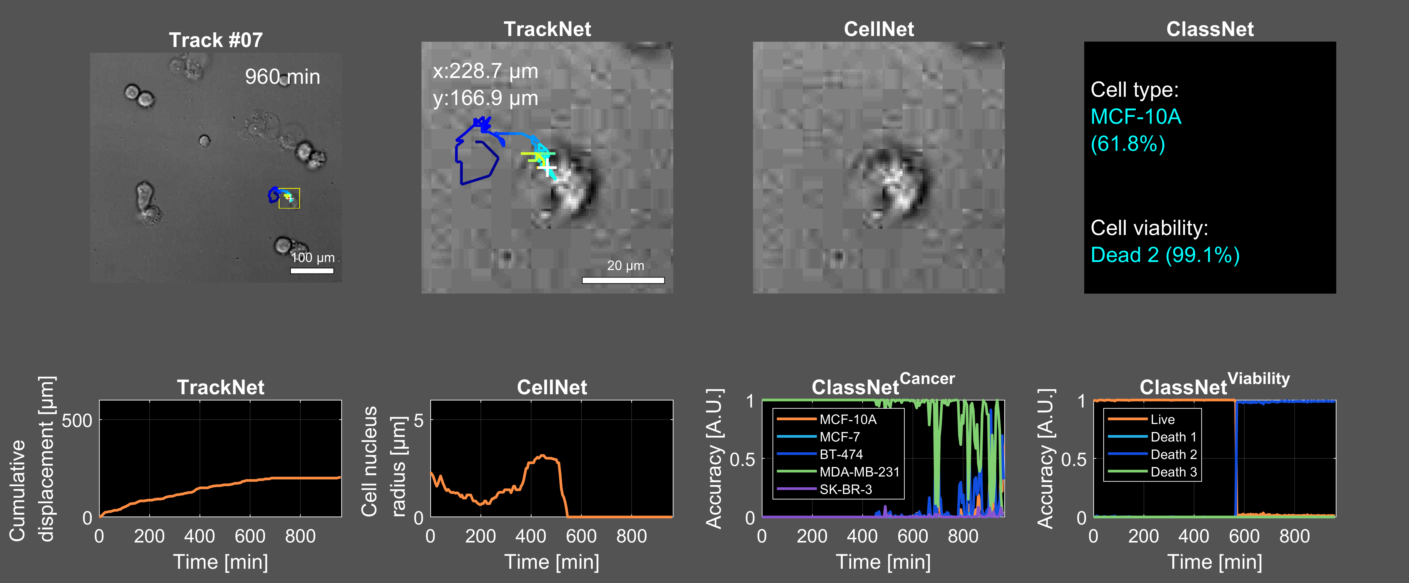


## Supplementary Video 3. Cancer cell tracking demonstration (Track #07)

Track #07 from **Supplementary Video 1** is shown in detail using AIM (**Fig.** **3a**−**3e**).


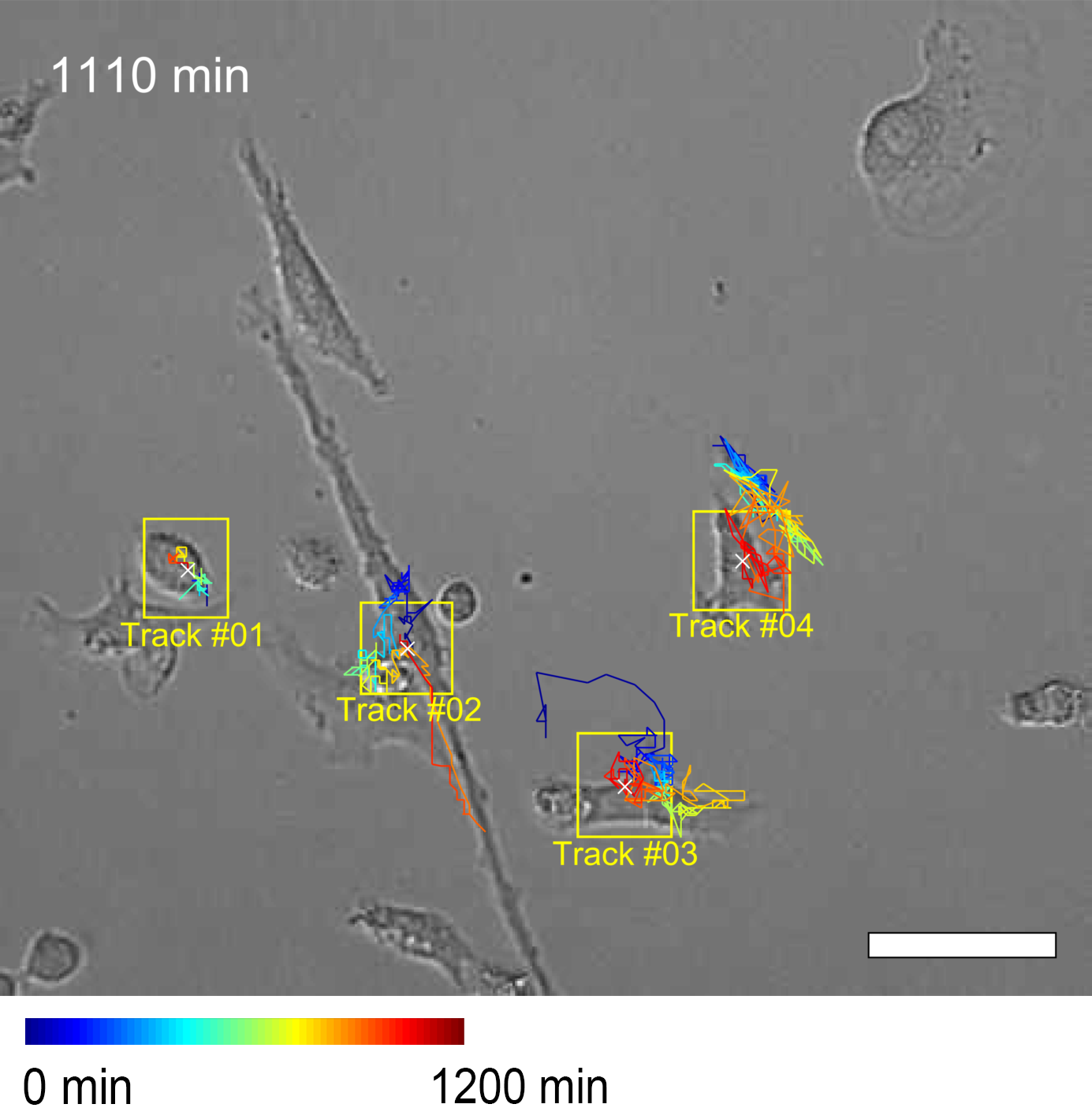


## Supplementary Video 4. Immature dendritic cell tracking demonstration (without lipoplysaccharide (LPS))

Immature dendritic cells were imaged for 20 h (see **Fig. 3** and **Methods**). The cell trajectories were found using TrackNet (**Methods** and **Supplementary Note 3**). Scale bar: 100 µm.


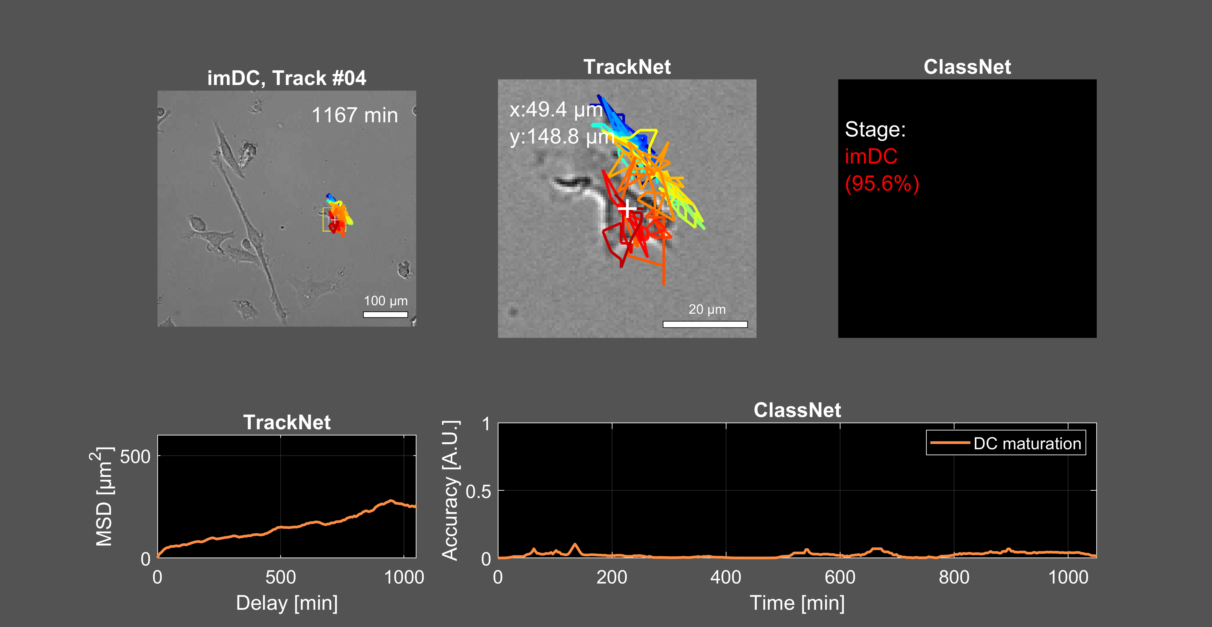


## Supplementary Video 5. Immature dendritic cell tracking demonstration (without lipoplysaccharide (LPS), Track #04)

Track #04 from **Supplementary Video 4** is shown in detail using AIM (**Fig.** **3f**−**3i**).


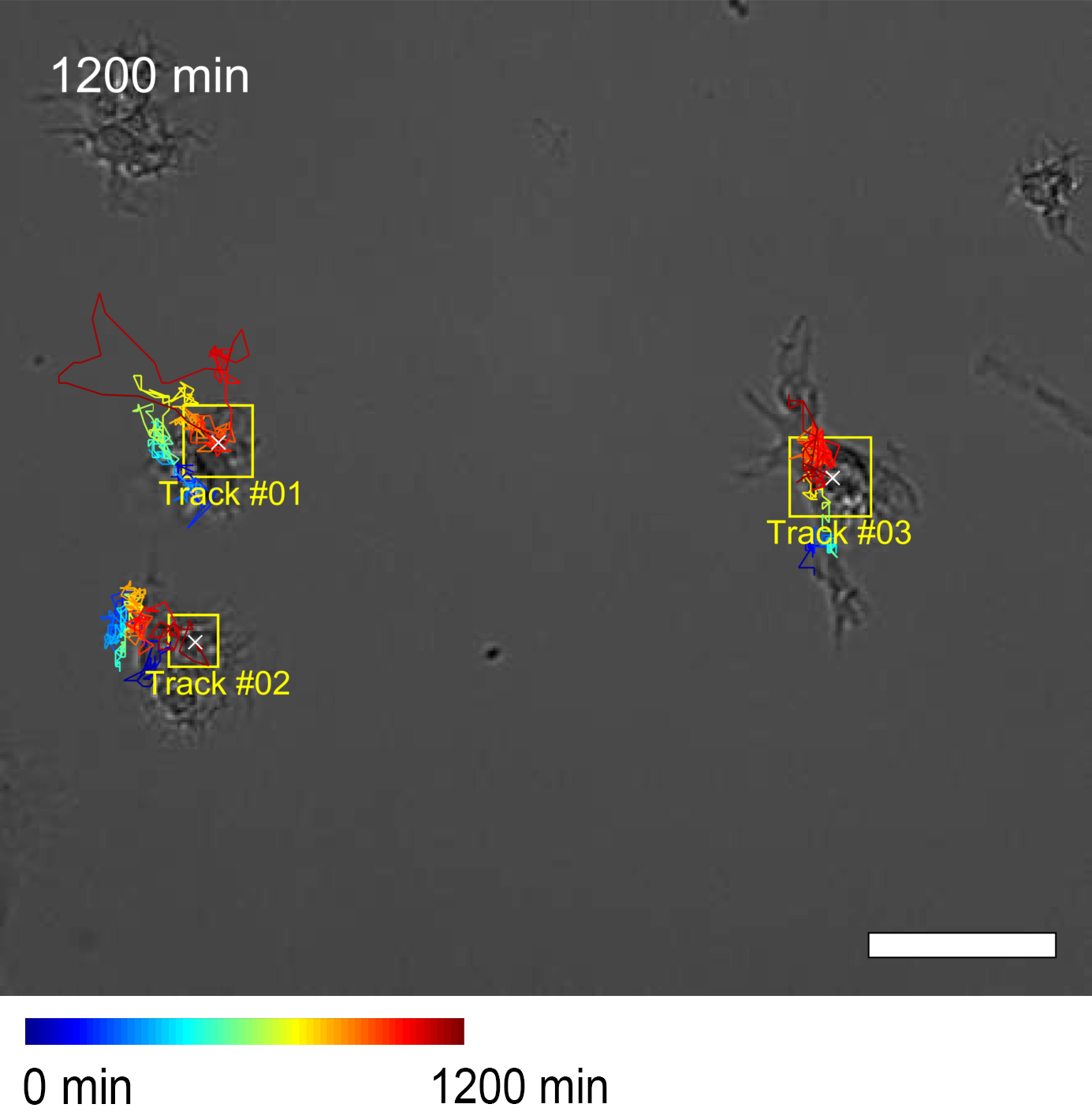


## Supplementary Video 6. Immature dendritic cell tracking demonstration (with lipoplysaccharide (LPS))

Immature dendritic cells were imaged for 20 h (see **Fig. 3** and **Methods**). LPS was added to stimulate dendritic cell maturation. The cell trajectories were found using TrackNet (**Methods** and **Supplementary Note 3**). Scale bar: 100 µm.


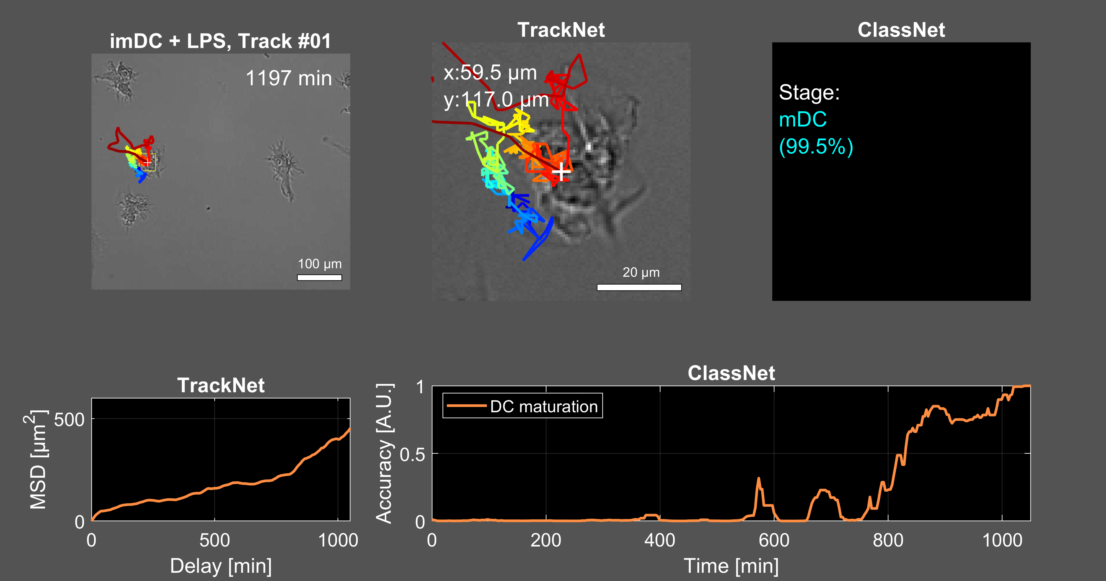


## Supplementary Video 7. Immature dendritic cell tracking demonstration (with lipoplysaccharide (LPS) , Track #01)

Track #01 from **Supplementary Video 6** is shown in detail using AIM (**Fig.** **3f**−**3i**).

# Supplementary Data

## Supplementary Data 1. CellNet^Nuc^ network details.

Please find **Supplementary Figure 7; Encoder depth = 3** for graphic visualization of layer connections.

|  | Name | Type | Activations | Learnables |  | TotalLearnables |
| --- | --- | --- | --- | --- | --- | --- |
| 1 | inputImage | Image Input | 101×101×1 | - |  | 0 |
|  | 101x101x1 images with 'zerocenter' normalization |  |  |  |  |  |
| 2 | encoder1_conv1 | Convolution | 101×101×64 | Weights | 3×3×1×64 | 640 |
|  | 64 3x3x1 convolutions with stride [1 1] and padding [1 1 1 1] |  |  | 1×1×64 |  |  |
| 3 | encoder1_bn_1 | Batch Normalization | 101×101×64 | Offset | 1×1×64 | 128 |
|  | Batch normalization with 64 channels |  |  | 1×1×64 |  |  |
| 4 | encoder1_relu_1 | ReLU | 101×101×64 | - |  | 0 |
|  | ReLU |  |  |  |  |  |
| 5 | encoder1_conv2 | Convolution | 101×101×64 | Weights | 3×3×64×64 | 36928 |
|  | 64 3x3x64 convolutions with stride [1 1] and padding [1 1 1 1] |  |  | 1×1×64 |  |  |
| 6 | encoder1_bn_2 | Batch Normalization | 101×101×64 | Offset | 1×1×64 | 128 |
|  | Batch normalization with 64 channels |  |  | 1×1×64 |  |  |
| 7 | encoder1_relu_2 | ReLU | 101×101×64 | - |  | 0 |
|  | ReLU |  |  |  |  |  |
| 8 | encoder1_maxpool | Max Pooling | out | - |  | 0 |
|  | 2x2 max pooling with stride [2 2] and padding [0 0 0 0] |  | 50×50×64 |  |  |  |
|  |  |  | 160000×1×1 |  |  |  |
|  |  |  | 1×4×1×101×101 |  |  |  |
| 9 | encoder2_conv1 | Convolution | 50×50×64 | Weights | 3×3×64×64 | 36928 |
|  | 64 3x3x64 convolutions with stride [1 1] and padding [1 1 1 1] |  |  | 1×1×64 |  |  |
| 10 | encoder2_bn_1 | Batch Normalization | 50×50×64 | Offset | 1×1×64 | 128 |
|  | Batch normalization with 64 channels |  |  | 1×1×64 |  |  |
| 11 | encoder2_relu_1 | ReLU | 50×50×64 | - |  | 0 |
|  | ReLU |  |  |  |  |  |
| 12 | encoder2_conv2 | Convolution | 50×50×64 | Weights | 3×3×64×64 | 36928 |
|  | 64 3x3x64 convolutions with stride [1 1] and padding [1 1 1 1] |  |  | 1×1×64 |  |  |
| 13 | encoder2_bn_2 | Batch Normalization | 50×50×64 | Offset | 1×1×64 | 128 |
|  | Batch normalization with 64 channels |  |  | 1×1×64 |  |  |
| 14 | encoder2_relu_2 | ReLU | 50×50×64 | - |  | 0 |
|  | ReLU |  |  |  |  |  |
| 15 | encoder2_maxpool | Max Pooling | out | - |  | 0 |
|  | 2x2 max pooling with stride [2 2] and padding [0 0 0 0] |  | 25×25×64 |  |  |  |
|  |  |  | 40000×1×1 |  | Weights |  |
|  |  |  | 1×4×1×50×50 |  | Bias |  |
| 16 | encoder3_conv1 | Convolution | 25×25×64 | Weights |  | 36928 |
|  | 64 3x3x64 convolutions with stride [1 1] and padding [1 1 1 1] |  |  | 1×1×64 | Scale |  |
| 17 | encoder3_bn_1 | Batch Normalization | 25×25×64 | Offset |  | 128 |
|  | Batch normalization with 64 channels |  |  | 1×1×64 |  |  |
| 18 | encoder3_relu_1 | ReLU | 25×25×64 | - |  | 0 |
|  | ReLU |  |  |  | Bias |  |
| 19 | encoder3_conv2 | Convolution | 25×25×64 | Weights |  | 36928 |
|  | 64 3x3x64 convolutions with stride [1 1] and padding [1 1 1 1] |  |  | 1×1×64 | Scale |  |
| 20 | encoder3_bn_2 | Batch Normalization | 25×25×64 | Offset |  | 128 |
|  | Batch normalization with 64 channels |  |  | 1×1×64 |  |  |
| 21 | encoder3_relu_2 | ReLU | 25×25×64 | - |  | 0 |
|  | ReLU |  |  |  |  |  |
| 22 | encoder3_maxpool | Max Pooling | out | - |  | 0 |
|  | 2x2 max pooling with stride [2 2] and padding [0 0 0 0] |  | 12×12×64 |  |  |  |
|  |  |  | 9216×1×1 |  |  |  |
|  |  |  | 1×4×1×25×25 |  |  |  |
| 23 | encoder4_conv1 | Convolution | 12×12×64 | Weights | 3×3×64×64 | 36928 |
|  | 64 3x3x64 convolutions with stride [1 1] and padding [1 1 1 1] |  |  | 1×1×64 |  |  |
| 24 | encoder4_bn_1 | Batch Normalization | 12×12×64 | Offset | 1×1×64 | 128 |
|  | Batch normalization with 64 channels |  |  | 1×1×64 |  |  |
| 25 | encoder4_relu_1 | ReLU | 12×12×64 | - |  | 0 |
|  | ReLU |  |  |  |  |  |
| 26 | encoder4_conv2 | Convolution | 12×12×64 | Weights | 3×3×64×64 | 36928 |
|  | 64 3x3x64 convolutions with stride [1 1] and padding [1 1 1 1] |  |  | 1×1×64 |  |  |
| 27 | encoder4_bn_2 | Batch Normalization | 12×12×64 | Offset | 1×1×64 | 128 |
|  | Batch normalization with 64 channels |  |  | 1×1×64 |  |  |
| 28 | encoder4_relu_2 | ReLU | 12×12×64 | - |  | 0 |
|  | ReLU |  |  |  |  |  |
| 29 | encoder4_maxpool | Max Pooling | out | - |  | 0 |
|  | 2x2 max pooling with stride [2 2] and padding [0 0 0 0] |  | 6×6×64 |  |  |  |
|  |  |  | 2304×1×1 |  | - |  |
|  |  |  | 1×4×1×12×12 |  |  |  |
| 30 | decoder4_unpool | Max Unpooling | 12×12×64 | - |  | 0 |
|  | Max Unpooling |  |  |  |  |  |
| 31 | decoder4_conv2 | Convolution | 12×12×64 | Weights | 3×3×64×64 | 36928 |
|  | 64 3x3x64 convolutions with stride [1 1] and padding [1 1 1 1] |  |  | 1×1×64 |  |  |
| 32 | decoder4_bn_2 | Batch Normalization | 12×12×64 | Offset | 1×1×64 | 128 |
|  | Batch normalization with 64 channels |  |  | 1×1×64 |  |  |
| 33 | decoder4_relu_2 | ReLU | 12×12×64 | - |  | 0 |
|  | ReLU |  |  |  |  |  |
| 34 | decoder4_conv1 | Convolution | 12×12×64 | Weights | 3×3×64×64 | 36928 |
|  | 64 3x3x64 convolutions with stride [1 1] and padding [1 1 1 1] |  |  | 1×1×64 |  |  |
| 35 | decoder4_bn_1 | Batch Normalization | 12×12×64 | Offset | 1×1×64 | 128 |
|  | Batch normalization with 64 channels |  |  | 1×1×64 |  |  |
| 36 | decoder4_relu_1 | ReLU | 12×12×64 | - |  | 0 |
|  | ReLU |  |  |  |  |  |
| 37 | decoder3_unpool | Max Unpooling | 25×25×64 | - |  | 0 |
|  | Max Unpooling |  |  |  |  |  |
| 38 | decoder3_conv2 | Convolution | 25×25×64 | Weights | 3×3×64×64 | 36928 |
|  | 64 3x3x64 convolutions with stride [1 1] and padding [1 1 1 1] |  |  | 1×1×64 |  |  |
| 39 | decoder3_bn_2 | Batch Normalization | 25×25×64 | Offset | 1×1×64 | 128 |
|  | Batch normalization with 64 channels |  |  | 1×1×64 |  |  |
| 40 | decoder3_relu_2 | ReLU | 25×25×64 | - |  | 0 |
|  | ReLU |  |  |  |  |  |
| 41 | decoder3_conv1 | Convolution | 25×25×64 | Weights | 3×3×64×64 | 36928 |
|  | 64 3x3x64 convolutions with stride [1 1] and padding [1 1 1 1] |  |  | 1×1×64 |  |  |
| 42 | decoder3_bn_1 | Batch Normalization | 25×25×64 | Offset | 1×1×64 | 128 |
|  | Batch normalization with 64 channels |  |  | 1×1×64 |  |  |
| 43 | decoder3_relu_1 | ReLU | 25×25×64 | - |  | 0 |
|  | ReLU |  |  |  |  |  |
| 44 | decoder2_unpool | Max Unpooling | 50×50×64 | - |  | 0 |
|  | Max Unpooling |  |  |  |  |  |
| 45 | decoder2_conv2 | Convolution | 50×50×64 | Weights | 3×3×64×64 | 36928 |
|  | 64 3x3x64 convolutions with stride [1 1] and padding [1 1 1 1] |  |  | 1×1×64 |  |  |
| 46 | decoder2_bn_2 | Batch Normalization | 50×50×64 | Offset | 1×1×64 | 128 |
|  | Batch normalization with 64 channels |  |  | 1×1×64 |  |  |
| 47 | decoder2_relu_2 | ReLU | 50×50×64 | - |  | 0 |
|  | ReLU |  |  |  |  |  |
| 48 | decoder2_conv1 | Convolution | 50×50×64 | Weights | 3×3×64×64 | 36928 |
|  | 64 3x3x64 convolutions with stride [1 1] and padding [1 1 1 1] |  |  | 1×1×64 |  |  |
| 49 | decoder2_bn_1 | Batch Normalization | 50×50×64 | Offset | 1×1×64 | 128 |
|  | Batch normalization with 64 channels |  |  | 1×1×64 |  |  |
| 50 | decoder2_relu_1 | ReLU | 50×50×64 | - |  | 0 |
|  | ReLU |  |  |  |  |  |
| 51 | decoder1_unpool | Max Unpooling | 101×101×64 | - |  | 0 |
|  | Max Unpooling |  |  |  |  |  |
| 52 | decoder1_conv2 | Convolution | 101×101×64 | Weights | 3×3×64×64 | 36928 |
|  | 64 3x3x64 convolutions with stride [1 1] and padding [1 1 1 1] |  |  | 1×1×64 |  |  |
| 53 | decoder1_bn_2 | Batch Normalization | 101×101×64 | Offset | 1×1×64 | 128 |
|  | Batch normalization with 64 channels |  |  | 1×1×64 |  |  |
| 54 | decoder1_relu_2 | ReLU | 101×101×64 | - |  | 0 |
|  | ReLU |  |  |  |  |  |
| 55 | decoder1_conv1 | Convolution | 101×101×2 | Weights | 3×3×64×2 | 1154 |
|  | 2 3x3x64 convolutions with stride [1 1] and padding [1 1 1 1] |  |  | 1×1×2 |  |  |
| 56 | decoder1_bn_1 | Batch Normalization | 101×101×2 | Offset | 1×1×2 | 4 |
|  | Batch normalization with 2 channels |  |  | 1×1×2 |  |  |
| 57 | decoder1_relu_1 | ReLU | 101×101×2 | - |  | 0 |
|  | ReLU |  |  |  |  |  |
| 58 | softmax | Softmax | 101×101×2 | - |  | 0 |
|  | softmax |  |  |  |  |  |
| 59 | pixelLabels | Pixel Classification Layer | - | - |  | 0 |
|  | Cross-entropy loss with classes 'nucleus' and 'background' |  |  |  |  |  |

## Supplementary Data 2. CellNet^Mito^ network details.

Please find **Supplementary Figure 7; Encoder depth = 2** for graphic visualization of layer connections.

|  | Name | Type | Activations | Learnables |  | TotalLearnables |
| --- | --- | --- | --- | --- | --- | --- |
| 1 | inputImage | Image Input | 101×101×1 | - |  | 0 |
|  | 101x101x1 images with 'zerocenter' normalization |  |  |  |  |  |
| 2 | encoder1_conv1 | Convolution | 101×101×64 | Weights | 3×3×1×64 | 640 |
|  | 64 3x3x1 convolutions with stride [1 1] and padding [1 1 1 1] |  |  | 1×1×64 |  |  |
| 3 | encoder1_bn_1 | Batch Normalization | 101×101×64 | Offset | 1×1×64 | 128 |
|  | Batch normalization with 64 channels |  |  | 1×1×64 |  |  |
| 4 | encoder1_relu_1 | ReLU | 101×101×64 | - |  | 0 |
|  | ReLU |  |  |  |  |  |
| 5 | encoder1_conv2 | Convolution | 101×101×64 | Weights | 3×3×64×64 | 36928 |
|  | 64 3x3x64 convolutions with stride [1 1] and padding [1 1 1 1] |  |  | 1×1×64 |  |  |
| 6 | encoder1_bn_2 | Batch Normalization | 101×101×64 | Offset | 1×1×64 | 128 |
|  | Batch normalization with 64 channels |  |  | 1×1×64 |  |  |
| 7 | encoder1_relu_2 | ReLU | 101×101×64 | - |  | 0 |
|  | ReLU |  |  |  |  |  |
| 8 | encoder1_maxpool | Max Pooling | out | - |  | 0 |
|  | 2x2 max pooling with stride [2 2] and padding [0 0 0 0] |  | 50×50×64 |  |  |  |
|  |  |  | 160000×1×1 |  |  |  |
|  |  |  | 1×4×1×101×101 |  |  |  |
| 9 | encoder2_conv1 | Convolution | 50×50×64 | Weights | 3×3×64×64 | 36928 |
|  | 64 3x3x64 convolutions with stride [1 1] and padding [1 1 1 1] |  |  | 1×1×64 |  |  |
| 10 | encoder2_bn_1 | Batch Normalization | 50×50×64 | Offset | 1×1×64 | 128 |
|  | Batch normalization with 64 channels |  |  | 1×1×64 |  |  |
| 11 | encoder2_relu_1 | ReLU | 50×50×64 | - |  | 0 |
|  | ReLU |  |  |  |  |  |
| 12 | encoder2_conv2 | Convolution | 50×50×64 | Weights | 3×3×64×64 | 36928 |
|  | 64 3x3x64 convolutions with stride [1 1] and padding [1 1 1 1] |  |  | 1×1×64 |  |  |
| 13 | encoder2_bn_2 | Batch Normalization | 50×50×64 | Offset | 1×1×64 | 128 |
|  | Batch normalization with 64 channels |  |  | 1×1×64 |  |  |
| 14 | encoder2_relu_2 | ReLU | 50×50×64 | - |  | 0 |
|  | ReLU |  |  |  |  |  |
| 15 | encoder2_maxpool | Max Pooling | out | - |  | 0 |
|  | 2x2 max pooling with stride [2 2] and padding [0 0 0 0] |  | 25×25×64 |  |  |  |
|  |  |  | 40000×1×1 |  |  |  |
|  |  |  | 1×4×1×50×50 |  |  |  |
| 16 | decoder2_unpool | Max Unpooling | 50×50×64 | - |  | 0 |
|  | Max Unpooling |  |  |  |  |  |
| 17 | decoder2_conv2 | Convolution | 50×50×64 | Weights | 3×3×64×64 | 36928 |
|  | 64 3x3x64 convolutions with stride [1 1] and padding [1 1 1 1] |  |  | 1×1×64 |  |  |
| 18 | decoder2_bn_2 | Batch Normalization | 50×50×64 | Offset | 1×1×64 | 128 |
|  | Batch normalization with 64 channels |  |  | 1×1×64 |  |  |
| 19 | decoder2_relu_2 | ReLU | 50×50×64 | - |  | 0 |
|  | ReLU |  |  |  |  |  |
| 20 | decoder2_conv1 | Convolution | 50×50×64 | Weights | 3×3×64×64 | 36928 |
|  | 64 3x3x64 convolutions with stride [1 1] and padding [1 1 1 1] |  |  | 1×1×64 |  |  |
| 21 | decoder2_bn_1 | Batch Normalization | 50×50×64 | Offset | 1×1×64 | 128 |
|  | Batch normalization with 64 channels |  |  | 1×1×64 |  |  |
| 22 | decoder2_relu_1 | ReLU | 50×50×64 | - |  | 0 |
|  | ReLU |  |  |  |  |  |
| 23 | decoder1_unpool | Max Unpooling | 101×101×64 | - |  | 0 |
|  | Max Unpooling |  |  |  |  |  |
| 24 | decoder1_conv2 | Convolution | 101×101×64 | Weights | 3×3×64×64 | 36928 |
|  | 64 3x3x64 convolutions with stride [1 1] and padding [1 1 1 1] |  |  | 1×1×64 |  |  |
| 25 | decoder1_bn_2 | Batch Normalization | 101×101×64 | Offset | 1×1×64 | 128 |
|  | Batch normalization with 64 channels |  |  | 1×1×64 |  |  |
| 26 | decoder1_relu_2 | ReLU | 101×101×64 | - |  | 0 |
|  | ReLU |  |  |  |  |  |
| 27 | decoder1_conv1 | Convolution | 101×101×2 | Weights | 3×3×64×2 | 1154 |
|  | 2 3x3x64 convolutions with stride [1 1] and padding [1 1 1 1] |  |  | 1×1×2 |  |  |
| 28 | decoder1_bn_1 | Batch Normalization | 101×101×2 | Offset | 1×1×2 | 4 |
|  | Batch normalization with 2 channels |  |  | 1×1×2 |  |  |
| 29 | decoder1_relu_1 | ReLU | 101×101×2 | - |  | 0 |
|  | ReLU |  |  |  |  |  |
| 30 | softmax | Softmax | 101×101×2 | - |  | 0 |
|  | softmax |  |  |  |  |  |
| 31 | pixelLabels | Pixel Classification Layer | - | - |  | 0 |
|  | Class weighted cross-entropy loss with classes '0' and '1' |  |  |  |  |  |

## Supplementary Data 3. CellNet^Actin^ network details.

Please find **Supplementary Figure 7; Encoder depth = 2** for graphic visualization of layer connections.

|  | Name | Type | Activations | Learnables |  | TotalLearnables |
| --- | --- | --- | --- | --- | --- | --- |
| 1 | inputImage | Image Input | 101×101×1 | - |  | 0 |
|  | 101x101x1 images with 'zerocenter' normalization |  |  |  |  |  |
| 2 | encoder1_conv1 | Convolution | 101×101×64 | Weights | 3×3×1×64 | 640 |
|  | 64 3x3x1 convolutions with stride [1 1] and padding [1 1 1 1] |  |  | 1×1×64 |  |  |
| 3 | encoder1_bn_1 | Batch Normalization | 101×101×64 | Offset | 1×1×64 | 128 |
|  | Batch normalization with 64 channels |  |  | 1×1×64 |  |  |
| 4 | encoder1_relu_1 | ReLU | 101×101×64 | - |  | 0 |
|  | ReLU |  |  |  |  |  |
| 5 | encoder1_conv2 | Convolution | 101×101×64 | Weights | 3×3×64×64 | 36928 |
|  | 64 3x3x64 convolutions with stride [1 1] and padding [1 1 1 1] |  |  | 1×1×64 |  |  |
| 6 | encoder1_bn_2 | Batch Normalization | 101×101×64 | Offset | 1×1×64 | 128 |
|  | Batch normalization with 64 channels |  |  | 1×1×64 |  |  |
| 7 | encoder1_relu_2 | ReLU | 101×101×64 | - |  | 0 |
|  | ReLU |  |  |  |  |  |
| 8 | encoder1_maxpool | Max Pooling | out | - |  | 0 |
|  | 2x2 max pooling with stride [2 2] and padding [0 0 0 0] |  | 50×50×64 |  |  |  |
|  |  |  | 160000×1×1 |  |  |  |
|  |  |  | 1×4×1×101×101 |  |  |  |
| 9 | encoder2_conv1 | Convolution | 50×50×64 | Weights | 3×3×64×64 | 36928 |
|  | 64 3x3x64 convolutions with stride [1 1] and padding [1 1 1 1] |  |  | 1×1×64 |  |  |
| 10 | encoder2_bn_1 | Batch Normalization | 50×50×64 | Offset | 1×1×64 | 128 |
|  | Batch normalization with 64 channels |  |  | 1×1×64 |  |  |
| 11 | encoder2_relu_1 | ReLU | 50×50×64 | - |  | 0 |
|  | ReLU |  |  |  |  |  |
| 12 | encoder2_conv2 | Convolution | 50×50×64 | Weights | 3×3×64×64 | 36928 |
|  | 64 3x3x64 convolutions with stride [1 1] and padding [1 1 1 1] |  |  | 1×1×64 |  |  |
| 13 | encoder2_bn_2 | Batch Normalization | 50×50×64 | Offset | 1×1×64 | 128 |
|  | Batch normalization with 64 channels |  |  | 1×1×64 |  |  |
| 14 | encoder2_relu_2 | ReLU | 50×50×64 | - |  | 0 |
|  | ReLU |  |  |  |  |  |
| 15 | encoder2_maxpool | Max Pooling | out | - |  | 0 |
|  | 2x2 max pooling with stride [2 2] and padding [0 0 0 0] |  | 25×25×64 |  |  |  |
|  |  |  | 40000×1×1 |  |  |  |
|  |  |  | 1×4×1×50×50 |  |  |  |
| 16 | decoder2_unpool | Max Unpooling | 50×50×64 | - |  | 0 |
|  | Max Unpooling |  |  |  |  |  |
| 17 | decoder2_conv2 | Convolution | 50×50×64 | Weights | 3×3×64×64 | 36928 |
|  | 64 3x3x64 convolutions with stride [1 1] and padding [1 1 1 1] |  |  | 1×1×64 |  |  |
| 18 | decoder2_bn_2 | Batch Normalization | 50×50×64 | Offset | 1×1×64 | 128 |
|  | Batch normalization with 64 channels |  |  | 1×1×64 |  |  |
| 19 | decoder2_relu_2 | ReLU | 50×50×64 | - |  | 0 |
|  | ReLU |  |  |  |  |  |
| 20 | decoder2_conv1 | Convolution | 50×50×64 | Weights | 3×3×64×64 | 36928 |
|  | 64 3x3x64 convolutions with stride [1 1] and padding [1 1 1 1] |  |  | 1×1×64 |  |  |
| 21 | decoder2_bn_1 | Batch Normalization | 50×50×64 | Offset | 1×1×64 | 128 |
|  | Batch normalization with 64 channels |  |  | 1×1×64 |  |  |
| 22 | decoder2_relu_1 | ReLU | 50×50×64 | - |  | 0 |
|  | ReLU |  |  |  |  |  |
| 23 | decoder1_unpool | Max Unpooling | 101×101×64 | - |  | 0 |
|  | Max Unpooling |  |  |  |  |  |
| 24 | decoder1_conv2 | Convolution | 101×101×64 | Weights | 3×3×64×64 | 36928 |
|  | 64 3x3x64 convolutions with stride [1 1] and padding [1 1 1 1] |  |  | 1×1×64 |  |  |
| 25 | decoder1_bn_2 | Batch Normalization | 101×101×64 | Offset | 1×1×64 | 128 |
|  | Batch normalization with 64 channels |  |  | 1×1×64 |  |  |
| 26 | decoder1_relu_2 | ReLU | 101×101×64 | - |  | 0 |
|  | ReLU |  |  |  |  |  |
| 27 | decoder1_conv1 | Convolution | 101×101×2 | Weights | 3×3×64×2 | 1154 |
|  | 2 3x3x64 convolutions with stride [1 1] and padding [1 1 1 1] |  |  | 1×1×2 |  |  |
| 28 | decoder1_bn_1 | Batch Normalization | 101×101×2 | Offset | 1×1×2 | 4 |
|  | Batch normalization with 2 channels |  |  | 1×1×2 |  |  |
| 29 | decoder1_relu_1 | ReLU | 101×101×2 | - |  | 0 |
|  | ReLU |  |  |  |  |  |
| 30 | softmax | Softmax | 101×101×2 | - |  | 0 |
|  | softmax |  |  |  |  |  |
| 31 | pixelLabels | Pixel Classification Layer | - | - |  | 0 |
|  | Class weighted cross-entropy loss with classes '0' and '1' |  |  |  |  |  |

## Supplementary Data 4. ClassNet^Viability^ network details.

Please find **Supplementary Figure 5; GoogleNet** for graphic visualization of layer connections.

|  | Name | Type | Activations | Learnables |  | TotalLearnables |
| --- | --- | --- | --- | --- | --- | --- |
| 1 | data | Image Input | 101×101×3 | - |  | 0 |
|  | 101x101x3 images with 'zerocenter' normalization |  |  |  |  |  |
| 2 | conv1-7x7_s2 | Convolution | 51×51×64 | Weights | 7×7×3×64 | 9472 |
|  | 64 7x7x3 convolutions with stride [2 2] and padding [3 3 3 3] |  |  | Bias | 1×1×64 |  |
| 3 | conv1-relu_7x7 | ReLU | 51×51×64 | - |  | 0 |
|  | ReLU |  |  |  |  |  |
| 4 | pool1-3x3_s2 | Max Pooling | 25×25×64 | - |  | 0 |
|  | 3x3 max pooling with stride [2 2] and padding [0 1 0 1] |  |  |  |  |  |
| 5 | pool1-norm1 | Cross Channel Normalization | 25×25×64 | - |  | 0 |
|  | cross channel normalization with 5 channels per element |  |  |  |  |  |
| 6 | conv2-3x3_reduce | Convolution | 25×25×64 | Weights | 1×1×64×64 | 4160 |
|  | 64 1x1x64 convolutions with stride [1 1] and padding [0 0 0 0] |  |  | Bias | 1×1×64 |  |
| 7 | conv2-relu_3x3_reduce | ReLU | 25×25×64 | - |  | 0 |
|  | ReLU |  |  |  |  |  |
| 8 | conv2-3x3 | Convolution | 25×25×192 | Weights | 3×3×64×192 | 110784 |
|  | 192 3x3x64 convolutions with stride [1 1] and padding [1 1 1 1] |  |  | Bias | 1×1×192 |  |
| 9 | conv2-relu_3x3 | ReLU | 25×25×192 | - |  | 0 |
|  | ReLU |  |  |  |  |  |
| 10 | conv2-norm2 | Cross Channel Normalization | 25×25×192 | - |  | 0 |
|  | cross channel normalization with 5 channels per element |  |  |  |  |  |
| 11 | pool2-3x3_s2 | Max Pooling | 12×12×192 | - |  | 0 |
|  | 3x3 max pooling with stride [2 2] and padding [0 1 0 1] |  |  |  |  |  |
| 12 | inception_3a-1x1 | Convolution | 12×12×64 | Weights | 1×1×192×64 | 12352 |
|  | 64 1x1x192 convolutions with stride [1 1] and padding [0 0 0 0] |  |  | Bias | 1×1×64 |  |
| 13 | inception_3a-relu_1x1 | ReLU | 12×12×64 | - |  | 0 |
|  | ReLU |  |  |  |  |  |
| 14 | inception_3a-3x3_reduce | Convolution | 12×12×96 | Weights | 1×1×192×96 | 18528 |
|  | 96 1x1x192 convolutions with stride [1 1] and padding [0 0 0 0] |  |  | Bias | 1×1×96 |  |
| 15 | inception_3a-relu_3x3_reduce | ReLU | 12×12×96 | - |  | 0 |
|  | ReLU |  |  |  |  |  |
| 16 | inception_3a-3x3 | Convolution | 12×12×128 | Weights | 3×3×96×128 | 110720 |
|  | 128 3x3x96 convolutions with stride [1 1] and padding [1 1 1 1] |  |  | Bias | 1×1×128 |  |
| 17 | inception_3a-relu_3x3 | ReLU | 12×12×128 | - |  | 0 |
|  | ReLU |  |  |  |  |  |
| 18 | inception_3a-5x5_reduce | Convolution | 12×12×16 | Weights | 1×1×192×16 | 3088 |
|  | 16 1x1x192 convolutions with stride [1 1] and padding [0 0 0 0] |  |  | Bias | 1×1×16 |  |
| 19 | inception_3a-relu_5x5_reduce | ReLU | 12×12×16 | - |  | 0 |
|  | ReLU |  |  |  |  |  |
| 20 | inception_3a-5x5 | Convolution | 12×12×32 | Weights | 5×5×16×32 | 12832 |
|  | 32 5x5x16 convolutions with stride [1 1] and padding [2 2 2 2] |  |  | Bias | 1×1×32 |  |
| 21 | inception_3a-relu_5x5 | ReLU | 12×12×32 | - |  | 0 |
|  | ReLU |  |  |  |  |  |
| 22 | inception_3a-pool | Max Pooling | 12×12×192 | - |  | 0 |
|  | 3x3 max pooling with stride [1 1] and padding [1 1 1 1] |  |  |  |  |  |
| 23 | inception_3a-pool_proj | Convolution | 12×12×32 | Weights | 1×1×192×32 | 6176 |
|  | 32 1x1x192 convolutions with stride [1 1] and padding [0 0 0 0] |  |  | Bias | 1×1×32 |  |
| 24 | inception_3a-relu_pool_proj | ReLU | 12×12×32 | - |  | 0 |
|  | ReLU |  |  |  |  |  |
| 25 | inception_3a-output | Depth concatenation | 12×12×256 | - |  | 0 |
|  | Depth concatenation of 4 inputs |  |  |  |  |  |
| 26 | inception_3b-1x1 | Convolution | 12×12×128 | Weights | 1×1×256×128 | 32896 |
|  | 128 1x1x256 convolutions with stride [1 1] and padding [0 0 0 0] |  |  | Bias | 1×1×128 |  |
| 27 | inception_3b-relu_1x1 | ReLU | 12×12×128 | - |  | 0 |
|  | ReLU |  |  |  |  |  |
| 28 | inception_3b-3x3_reduce | Convolution | 12×12×128 | Weights | 1×1×256×128 | 32896 |
|  | 128 1x1x256 convolutions with stride [1 1] and padding [0 0 0 0] |  |  | Bias | 1×1×128 |  |
| 29 | inception_3b-relu_3x3_reduce | ReLU | 12×12×128 | - |  | 0 |
|  | ReLU |  |  |  |  |  |
| 30 | inception_3b-3x3 | Convolution | 12×12×192 | Weights | 3×3×128×192 | 221376 |
|  | 192 3x3x128 convolutions with stride [1 1] and padding [1 1 1 1] |  |  | Bias | 1×1×192 |  |
| 31 | inception_3b-relu_3x3 | ReLU | 12×12×192 | - |  | 0 |
|  | ReLU |  |  |  |  |  |
| 32 | inception_3b-5x5_reduce | Convolution | 12×12×32 | Weights | 1×1×256×32 | 8224 |
|  | 32 1x1x256 convolutions with stride [1 1] and padding [0 0 0 0] |  |  | Bias | 1×1×32 |  |
| 33 | inception_3b-relu_5x5_reduce | ReLU | 12×12×32 | - |  | 0 |
|  | ReLU |  |  |  |  |  |
| 34 | inception_3b-5x5 | Convolution | 12×12×96 | Weights | 5×5×32×96 | 76896 |
|  | 96 5x5x32 convolutions with stride [1 1] and padding [2 2 2 2] |  |  | Bias | 1×1×96 |  |
| 35 | inception_3b-relu_5x5 | ReLU | 12×12×96 | - |  | 0 |
|  | ReLU |  |  |  |  |  |
| 36 | inception_3b-pool | Max Pooling | 12×12×256 | - |  | 0 |
|  | 3x3 max pooling with stride [1 1] and padding [1 1 1 1] |  |  |  |  |  |
| 37 | inception_3b-pool_proj | Convolution | 12×12×64 | Weights | 1×1×256×64 | 16448 |
|  | 64 1x1x256 convolutions with stride [1 1] and padding [0 0 0 0] |  |  | Bias | 1×1×64 |  |
| 38 | inception_3b-relu_pool_proj | ReLU | 12×12×64 | - |  | 0 |
|  | ReLU |  |  |  |  |  |
| 39 | inception_3b-output | Depth concatenation | 12×12×480 | - |  | 0 |
|  | Depth concatenation of 4 inputs |  |  |  |  |  |
| 40 | pool3-3x3_s2 | Max Pooling | 6×6×480 | - |  | 0 |
|  | 3x3 max pooling with stride [2 2] and padding [0 1 0 1] |  |  |  |  |  |
| 41 | inception_4a-1x1 | Convolution | 6×6×192 | Weights | 1×1×480×192 | 92352 |
|  | 192 1x1x480 convolutions with stride [1 1] and padding [0 0 0 0] |  |  | Bias | 1×1×192 |  |
| 42 | inception_4a-relu_1x1 | ReLU | 6×6×192 | - |  | 0 |
|  | ReLU |  |  |  |  |  |
| 43 | inception_4a-3x3_reduce | Convolution | 6×6×96 | Weights | 1×1×480×96 | 46176 |
|  | 96 1x1x480 convolutions with stride [1 1] and padding [0 0 0 0] |  |  | Bias | 1×1×96 |  |
| 44 | inception_4a-relu_3x3_reduce | ReLU | 6×6×96 | - |  | 0 |
|  | ReLU |  |  |  |  |  |
| 45 | inception_4a-3x3 | Convolution | 6×6×208 | Weights | 3×3×96×208 | 179920 |
|  | 208 3x3x96 convolutions with stride [1 1] and padding [1 1 1 1] |  |  | Bias | 1×1×208 |  |
| 46 | inception_4a-relu_3x3 | ReLU | 6×6×208 | - |  | 0 |
|  | ReLU |  |  |  |  |  |
| 47 | inception_4a-5x5_reduce | Convolution | 6×6×16 | Weights | 1×1×480×16 | 7696 |
|  | 16 1x1x480 convolutions with stride [1 1] and padding [0 0 0 0] |  |  | Bias | 1×1×16 |  |
| 48 | inception_4a-relu_5x5_reduce | ReLU | 6×6×16 | - |  | 0 |
|  | ReLU |  |  |  |  |  |
| 49 | inception_4a-5x5 | Convolution | 6×6×48 | Weights | 5×5×16×48 | 19248 |
|  | 48 5x5x16 convolutions with stride [1 1] and padding [2 2 2 2] |  |  | Bias | 1×1×48 |  |
| 50 | inception_4a-relu_5x5 | ReLU | 6×6×48 | - |  | 0 |
|  | ReLU |  |  |  |  |  |
| 51 | inception_4a-pool | Max Pooling | 6×6×480 | - |  | 0 |
|  | 3x3 max pooling with stride [1 1] and padding [1 1 1 1] |  |  |  |  |  |
| 52 | inception_4a-pool_proj | Convolution | 6×6×64 | Weights | 1×1×480×64 | 30784 |
|  | 64 1x1x480 convolutions with stride [1 1] and padding [0 0 0 0] |  |  | Bias | 1×1×64 |  |
| 53 | inception_4a-relu_pool_proj | ReLU | 6×6×64 | - |  | 0 |
|  | ReLU |  |  |  |  |  |
| 54 | inception_4a-output | Depth concatenation | 6×6×512 | - |  | 0 |
|  | Depth concatenation of 4 inputs |  |  |  |  |  |
| 55 | inception_4b-1x1 | Convolution | 6×6×160 | Weights | 1×1×512×160 | 82080 |
|  | 160 1x1x512 convolutions with stride [1 1] and padding [0 0 0 0] |  |  | Bias | 1×1×160 |  |
| 56 | inception_4b-relu_1x1 | ReLU | 6×6×160 | - |  | 0 |
|  | ReLU |  |  |  |  |  |
| 57 | inception_4b-3x3_reduce | Convolution | 6×6×112 | Weights | 1×1×512×112 | 57456 |
|  | 112 1x1x512 convolutions with stride [1 1] and padding [0 0 0 0] |  |  | Bias | 1×1×112 |  |
| 58 | inception_4b-relu_3x3_reduce | ReLU | 6×6×112 | - |  | 0 |
|  | ReLU |  |  |  |  |  |
| 59 | inception_4b-3x3 | Convolution | 6×6×224 | Weights | 3×3×112×224 | 226016 |
|  | 224 3x3x112 convolutions with stride [1 1] and padding [1 1 1 1] |  |  | Bias | 1×1×224 |  |
| 60 | inception_4b-relu_3x3 | ReLU | 6×6×224 | - |  | 0 |
|  | ReLU |  |  |  |  |  |
| 61 | inception_4b-5x5_reduce | Convolution | 6×6×24 | Weights | 1×1×512×24 | 12312 |
|  | 24 1x1x512 convolutions with stride [1 1] and padding [0 0 0 0] |  |  | Bias | 1×1×24 |  |
| 62 | inception_4b-relu_5x5_reduce | ReLU | 6×6×24 | - |  | 0 |
|  | ReLU |  |  |  |  |  |
| 63 | inception_4b-5x5 | Convolution | 6×6×64 | Weights | 5×5×24×64 | 38464 |
|  | 64 5x5x24 convolutions with stride [1 1] and padding [2 2 2 2] |  |  | Bias | 1×1×64 |  |
| 64 | inception_4b-relu_5x5 | ReLU | 6×6×64 | - |  | 0 |
|  | ReLU |  |  |  |  |  |
| 65 | inception_4b-pool | Max Pooling | 6×6×512 | - |  | 0 |
|  | 3x3 max pooling with stride [1 1] and padding [1 1 1 1] |  |  |  |  |  |
| 66 | inception_4b-pool_proj | Convolution | 6×6×64 | Weights | 1×1×512×64 | 32832 |
|  | 64 1x1x512 convolutions with stride [1 1] and padding [0 0 0 0] |  |  | Bias | 1×1×64 |  |
| 67 | inception_4b-relu_pool_proj | ReLU | 6×6×64 | - |  | 0 |
|  | ReLU |  |  |  |  |  |
| 68 | inception_4b-output | Depth concatenation | 6×6×512 | - |  | 0 |
|  | Depth concatenation of 4 inputs |  |  |  |  |  |
| 69 | inception_4c-1x1 | Convolution | 6×6×128 | Weights | 1×1×512×128 | 65664 |
|  | 128 1x1x512 convolutions with stride [1 1] and padding [0 0 0 0] |  |  | Bias | 1×1×128 |  |
| 70 | inception_4c-relu_1x1 | ReLU | 6×6×128 | - |  | 0 |
|  | ReLU |  |  |  |  |  |
| 71 | inception_4c-3x3_reduce | Convolution | 6×6×128 | Weights | 1×1×512×128 | 65664 |
|  | 128 1x1x512 convolutions with stride [1 1] and padding [0 0 0 0] |  |  | Bias | 1×1×128 |  |
| 72 | inception_4c-relu_3x3_reduce | ReLU | 6×6×128 | - |  | 0 |
|  | ReLU |  |  |  |  |  |
| 73 | inception_4c-3x3 | Convolution | 6×6×256 | Weights | 3×3×128×256 | 295168 |
|  | 256 3x3x128 convolutions with stride [1 1] and padding [1 1 1 1] |  |  | Bias | 1×1×256 |  |
| 74 | inception_4c-relu_3x3 | ReLU | 6×6×256 | - |  | 0 |
|  | ReLU |  |  |  |  |  |
| 75 | inception_4c-5x5_reduce | Convolution | 6×6×24 | Weights | 1×1×512×24 | 12312 |
|  | 24 1x1x512 convolutions with stride [1 1] and padding [0 0 0 0] |  |  | Bias | 1×1×24 |  |
| 76 | inception_4c-relu_5x5_reduce | ReLU | 6×6×24 | - |  | 0 |
|  | ReLU |  |  |  |  |  |
| 77 | inception_4c-5x5 | Convolution | 6×6×64 | Weights | 5×5×24×64 | 38464 |
|  | 64 5x5x24 convolutions with stride [1 1] and padding [2 2 2 2] |  |  | Bias | 1×1×64 |  |
| 78 | inception_4c-relu_5x5 | ReLU | 6×6×64 | - |  | 0 |
|  | ReLU |  |  |  |  |  |
| 79 | inception_4c-pool | Max Pooling | 6×6×512 | - |  | 0 |
|  | 3x3 max pooling with stride [1 1] and padding [1 1 1 1] |  |  |  |  |  |
| 80 | inception_4c-pool_proj | Convolution | 6×6×64 | Weights | 1×1×512×64 | 32832 |
|  | 64 1x1x512 convolutions with stride [1 1] and padding [0 0 0 0] |  |  | Bias | 1×1×64 |  |
| 81 | inception_4c-relu_pool_proj | ReLU | 6×6×64 | - |  | 0 |
|  | ReLU |  |  |  |  |  |
| 82 | inception_4c-output | Depth concatenation | 6×6×512 | - |  | 0 |
|  | Depth concatenation of 4 inputs |  |  |  |  |  |
| 83 | inception_4d-1x1 | Convolution | 6×6×112 | Weights | 1×1×512×112 | 57456 |
|  | 112 1x1x512 convolutions with stride [1 1] and padding [0 0 0 0] |  |  | Bias | 1×1×112 |  |
| 84 | inception_4d-relu_1x1 | ReLU | 6×6×112 | - |  | 0 |
|  | ReLU |  |  |  |  |  |
| 85 | inception_4d-3x3_reduce | Convolution | 6×6×144 | Weights | 1×1×512×144 | 73872 |
|  | 144 1x1x512 convolutions with stride [1 1] and padding [0 0 0 0] |  |  | Bias | 1×1×144 |  |
| 86 | inception_4d-relu_3x3_reduce | ReLU | 6×6×144 | - |  | 0 |
|  | ReLU |  |  |  |  |  |
| 87 | inception_4d-3x3 | Convolution | 6×6×288 | Weights | 3×3×144×288 | 373536 |
|  | 288 3x3x144 convolutions with stride [1 1] and padding [1 1 1 1] |  |  | Bias | 1×1×288 |  |
| 88 | inception_4d-relu_3x3 | ReLU | 6×6×288 | - |  | 0 |
|  | ReLU |  |  |  |  |  |
| 89 | inception_4d-5x5_reduce | Convolution | 6×6×32 | Weights | 1×1×512×32 | 16416 |
|  | 32 1x1x512 convolutions with stride [1 1] and padding [0 0 0 0] |  |  | Bias | 1×1×32 |  |
| 90 | inception_4d-relu_5x5_reduce | ReLU | 6×6×32 | - |  | 0 |
|  | ReLU |  |  |  |  |  |
| 91 | inception_4d-5x5 | Convolution | 6×6×64 | Weights | 5×5×32×64 | 51264 |
|  | 64 5x5x32 convolutions with stride [1 1] and padding [2 2 2 2] |  |  | Bias | 1×1×64 |  |
| 92 | inception_4d-relu_5x5 | ReLU | 6×6×64 | - |  | 0 |
|  | ReLU |  |  |  |  |  |
| 93 | inception_4d-pool | Max Pooling | 6×6×512 | - |  | 0 |
|  | 3x3 max pooling with stride [1 1] and padding [1 1 1 1] |  |  |  |  |  |
| 94 | inception_4d-pool_proj | Convolution | 6×6×64 | Weights | 1×1×512×64 | 32832 |
|  | 64 1x1x512 convolutions with stride [1 1] and padding [0 0 0 0] |  |  | Bias | 1×1×64 |  |
| 95 | inception_4d-relu_pool_proj | ReLU | 6×6×64 | - |  | 0 |
|  | ReLU |  |  |  |  |  |
| 96 | inception_4d-output | Depth concatenation | 6×6×528 | - |  | 0 |
|  | Depth concatenation of 4 inputs |  |  |  |  |  |
| 97 | inception_4e-1x1 | Convolution | 6×6×256 | Weights | 1×1×528×256 | 135424 |
|  | 256 1x1x528 convolutions with stride [1 1] and padding [0 0 0 0] |  |  | Bias | 1×1×256 |  |
| 98 | inception_4e-relu_1x1 | ReLU | 6×6×256 | - |  | 0 |
|  | ReLU |  |  |  |  |  |
| 99 | inception_4e-3x3_reduce | Convolution | 6×6×160 | Weights | 1×1×528×160 | 84640 |
|  | 160 1x1x528 convolutions with stride [1 1] and padding [0 0 0 0] |  |  | Bias | 1×1×160 |  |
| 100 | inception_4e-relu_3x3_reduce | ReLU | 6×6×160 | - |  | 0 |
|  | ReLU |  |  |  |  |  |
| 101 | inception_4e-3x3 | Convolution | 6×6×320 | Weights | 3×3×160×320 | 461120 |
|  | 320 3x3x160 convolutions with stride [1 1] and padding [1 1 1 1] |  |  | Bias | 1×1×320 |  |
| 102 | inception_4e-relu_3x3 | ReLU | 6×6×320 | - |  | 0 |
|  | ReLU |  |  |  |  |  |
| 103 | inception_4e-5x5_reduce | Convolution | 6×6×32 | Weights | 1×1×528×32 | 16928 |
|  | 32 1x1x528 convolutions with stride [1 1] and padding [0 0 0 0] |  |  | Bias | 1×1×32 |  |
| 104 | inception_4e-relu_5x5_reduce | ReLU | 6×6×32 | - |  | 0 |
|  | ReLU |  |  |  |  |  |
| 105 | inception_4e-5x5 | Convolution | 6×6×128 | Weights | 5×5×32×128 | 102528 |
|  | 128 5x5x32 convolutions with stride [1 1] and padding [2 2 2 2] |  |  | Bias | 1×1×128 |  |
| 106 | inception_4e-relu_5x5 | ReLU | 6×6×128 | - |  | 0 |
|  | ReLU |  |  |  |  |  |
| 107 | inception_4e-pool | Max Pooling | 6×6×528 | - |  | 0 |
|  | 3x3 max pooling with stride [1 1] and padding [1 1 1 1] |  |  |  |  |  |
| 108 | inception_4e-pool_proj | Convolution | 6×6×128 | Weights | 1×1×528×128 | 67712 |
|  | 128 1x1x528 convolutions with stride [1 1] and padding [0 0 0 0] |  |  | Bias | 1×1×128 |  |
| 109 | inception_4e-relu_pool_proj | ReLU | 6×6×128 | - |  | 0 |
|  | ReLU |  |  |  |  |  |
| 110 | inception_4e-output | Depth concatenation | 6×6×832 | - |  | 0 |
|  | Depth concatenation of 4 inputs |  |  |  |  |  |
| 111 | pool4-3x3_s2 | Max Pooling | 3×3×832 | - |  | 0 |
|  | 3x3 max pooling with stride [2 2] and padding [0 1 0 1] |  |  |  |  |  |
| 112 | inception_5a-1x1 | Convolution | 3×3×256 | Weights | 1×1×832×256 | 213248 |
|  | 256 1x1x832 convolutions with stride [1 1] and padding [0 0 0 0] |  |  | Bias | 1×1×256 |  |
| 113 | inception_5a-relu_1x1 | ReLU | 3×3×256 | - |  | 0 |
|  | ReLU |  |  |  |  |  |
| 114 | inception_5a-3x3_reduce | Convolution | 3×3×160 | Weights | 1×1×832×160 | 133280 |
|  | 160 1x1x832 convolutions with stride [1 1] and padding [0 0 0 0] |  |  | Bias | 1×1×160 |  |
| 115 | inception_5a-relu_3x3_reduce | ReLU | 3×3×160 | - |  | 0 |
|  | ReLU |  |  |  |  |  |
| 116 | inception_5a-3x3 | Convolution | 3×3×320 | Weights | 3×3×160×320 | 461120 |
|  | 320 3x3x160 convolutions with stride [1 1] and padding [1 1 1 1] |  |  | Bias | 1×1×320 |  |
| 117 | inception_5a-relu_3x3 | ReLU | 3×3×320 | - |  | 0 |
|  | ReLU |  |  |  |  |  |
| 118 | inception_5a-5x5_reduce | Convolution | 3×3×32 | Weights | 1×1×832×32 | 26656 |
|  | 32 1x1x832 convolutions with stride [1 1] and padding [0 0 0 0] |  |  | Bias | 1×1×32 |  |
| 119 | inception_5a-relu_5x5_reduce | ReLU | 3×3×32 | - |  | 0 |
|  | ReLU |  |  |  |  |  |
| 120 | inception_5a-5x5 | Convolution | 3×3×128 | Weights | 5×5×32×128 | 102528 |
|  | 128 5x5x32 convolutions with stride [1 1] and padding [2 2 2 2] |  |  | Bias | 1×1×128 |  |
| 121 | inception_5a-relu_5x5 | ReLU | 3×3×128 | - |  | 0 |
|  | ReLU |  |  |  |  |  |
| 122 | inception_5a-pool | Max Pooling | 3×3×832 | - |  | 0 |
|  | 3x3 max pooling with stride [1 1] and padding [1 1 1 1] |  |  |  |  |  |
| 123 | inception_5a-pool_proj | Convolution | 3×3×128 | Weights | 1×1×832×128 | 106624 |
|  | 128 1x1x832 convolutions with stride [1 1] and padding [0 0 0 0] |  |  | Bias | 1×1×128 |  |
| 124 | inception_5a-relu_pool_proj | ReLU | 3×3×128 | - |  | 0 |
|  | ReLU |  |  |  |  |  |
| 125 | inception_5a-output | Depth concatenation | 3×3×832 | - |  | 0 |
|  | Depth concatenation of 4 inputs |  |  |  |  |  |
| 126 | inception_5b-1x1 | Convolution | 3×3×384 | Weights | 1×1×832×384 | 319872 |
|  | 384 1x1x832 convolutions with stride [1 1] and padding [0 0 0 0] |  |  | Bias | 1×1×384 |  |
| 127 | inception_5b-relu_1x1 | ReLU | 3×3×384 | - |  | 0 |
|  | ReLU |  |  |  |  |  |
| 128 | inception_5b-3x3_reduce | Convolution | 3×3×192 | Weights | 1×1×832×192 | 159936 |
|  | 192 1x1x832 convolutions with stride [1 1] and padding [0 0 0 0] |  |  | Bias | 1×1×192 |  |
| 129 | inception_5b-relu_3x3_reduce | ReLU | 3×3×192 | - |  | 0 |
|  | ReLU |  |  |  |  |  |
| 130 | inception_5b-3x3 | Convolution | 3×3×384 | Weights | 3×3×192×384 | 663936 |
|  | 384 3x3x192 convolutions with stride [1 1] and padding [1 1 1 1] |  |  | Bias | 1×1×384 |  |
| 131 | inception_5b-relu_3x3 | ReLU | 3×3×384 | - |  | 0 |
|  | ReLU |  |  |  |  |  |
| 132 | inception_5b-5x5_reduce | Convolution | 3×3×48 | Weights | 1×1×832×48 | 39984 |
|  | 48 1x1x832 convolutions with stride [1 1] and padding [0 0 0 0] |  |  | Bias | 1×1×48 |  |
| 133 | inception_5b-relu_5x5_reduce | ReLU | 3×3×48 | - |  | 0 |
|  | ReLU |  |  |  |  |  |
| 134 | inception_5b-5x5 | Convolution | 3×3×128 | Weights | 5×5×48×128 | 153728 |
|  | 128 5x5x48 convolutions with stride [1 1] and padding [2 2 2 2] |  |  | Bias | 1×1×128 |  |
| 135 | inception_5b-relu_5x5 | ReLU | 3×3×128 | - |  | 0 |
|  | ReLU |  |  |  |  |  |
| 136 | inception_5b-pool | Max Pooling | 3×3×832 | - |  | 0 |
|  | 3x3 max pooling with stride [1 1] and padding [1 1 1 1] |  |  |  |  |  |
| 137 | inception_5b-pool_proj | Convolution | 3×3×128 | Weights | 1×1×832×128 | 106624 |
|  | 128 1x1x832 convolutions with stride [1 1] and padding [0 0 0 0] |  |  | Bias | 1×1×128 |  |
| 138 | inception_5b-relu_pool_proj | ReLU | 3×3×128 | - |  | 0 |
|  | ReLU |  |  |  |  |  |
| 139 | inception_5b-output | Depth concatenation | 3×3×1024 | - |  | 0 |
|  | Depth concatenation of 4 inputs |  |  |  |  |  |
| 140 | pool5-7x7_s1 | Average Pooling | 3×3×1024 | - |  | 0 |
|  | 1x1 average pooling with stride [1 1] and padding [0 0 0 0] |  |  |  |  |  |
| 141 | pool5-drop_7x7_s1 | Dropout | 3×3×1024 | - |  | 0 |
|  | 40% dropout |  |  |  |  |  |
| 142 | loss3-classifier | Fully Connected | 1×1×4 | Weights | 4×9216 | 36868 |
|  | 4 fully connected layer |  |  | Bias | 4×1 |  |
| 143 | prob | Softmax | 1×1×4 | - |  | 0 |
|  | softmax |  |  |  |  |  |
| 144 | output | Classification Output | - | - |  | 0 |
|  | crossentropyex with 'live' and 3 other classes |  |  |  |  |  |

## Supplementary Data 5. ClassNet^Cancer^ network details.

Please find **Supplementary Figure 5; GoogleNet** for graphic visualization of layer connections.

|  | Name | Type | Activations | Learnables |  | TotalLearnables |
| --- | --- | --- | --- | --- | --- | --- |
| 1 | data | Image Input | 101×101×3 | - |  | 0 |
|  | 101x101x3 images with 'zerocenter' normalization |  |  |  |  |  |
| 2 | conv1-7x7_s2 | Convolution | 51×51×64 | Weights | 7×7×3×64 | 9472 |
|  | 64 7x7x3 convolutions with stride [2 2] and padding [3 3 3 3] |  |  | Bias | 1×1×64 |  |
| 3 | conv1-relu_7x7 | ReLU | 51×51×64 | - |  | 0 |
|  | ReLU |  |  |  |  |  |
| 4 | pool1-3x3_s2 | Max Pooling | 25×25×64 | - |  | 0 |
|  | 3x3 max pooling with stride [2 2] and padding [0 1 0 1] |  |  |  |  |  |
| 5 | pool1-norm1 | Cross Channel Normalization | 25×25×64 | - | - | 0 |
|  | cross channel normalization with 5 channels per element |  |  |  |  |  |
| 6 | conv2-3x3_reduce | Convolution | 25×25×64 | Weights | 1×1×64×64 | 4160 |
|  | 64 1x1x64 convolutions with stride [1 1] and padding [0 0 0 0] |  |  | Bias | 1×1×64 |  |
| 7 | conv2-relu_3x3_reduce | ReLU | 25×25×64 | - |  | 0 |
|  | ReLU |  |  |  |  |  |
| 8 | conv2-3x3 | Convolution | 25×25×192 | Weights | 3×3×64×192 | 110784 |
|  | 192 3x3x64 convolutions with stride [1 1] and padding [1 1 1 1] |  |  | Bias | 1×1×192 |  |
| 9 | conv2-relu_3x3 | ReLU | 25×25×192 | - |  | 0 |
|  | ReLU |  |  |  |  |  |
| 10 | conv2-norm2 | Cross Channel Normalization | 25×25×192 | - |  | 0 |
|  | cross channel normalization with 5 channels per element |  |  |  |  |  |
| 11 | pool2-3x3_s2 | Max Pooling | 12×12×192 | - |  | 0 |
|  | 3x3 max pooling with stride [2 2] and padding [0 1 0 1] |  |  |  |  |  |
| 12 | inception_3a-1x1 | Convolution | 12×12×64 | Weights | 1×1×192×64 | 12352 |
|  | 64 1x1x192 convolutions with stride [1 1] and padding [0 0 0 0] |  |  | Bias | 1×1×64 |  |
| 13 | inception_3a-relu_1x1 | ReLU | 12×12×64 | - |  | 0 |
|  | ReLU |  |  |  |  |  |
| 14 | inception_3a-3x3_reduce | Convolution | 12×12×96 | Weights | 1×1×192×96 | 18528 |
|  | 96 1x1x192 convolutions with stride [1 1] and padding [0 0 0 0] |  |  | Bias | 1×1×96 |  |
| 15 | inception_3a-relu_3x3_reduce | ReLU | 12×12×96 | - | Weights | 0 |
|  | ReLU |  |  |  |  |  |
| 16 | inception_3a-3x3 | Convolution | 12×12×128 | Weights | 3×3×96×128 | 110720 |
|  | 128 3x3x96 convolutions with stride [1 1] and padding [1 1 1 1] |  |  | Bias | 1×1×128 |  |
| 17 | inception_3a-relu_3x3 | ReLU | 12×12×128 | - |  | 0 |
|  | ReLU |  |  |  |  |  |
| 18 | inception_3a-5x5_reduce | Convolution | 12×12×16 | Weights | 1×1×192×16 | 3088 |
|  | 16 1x1x192 convolutions with stride [1 1] and padding [0 0 0 0] |  |  | Bias | 1×1×16 |  |
| 19 | inception_3a-relu_5x5_reduce | ReLU | 12×12×16 | - |  | 0 |
|  | ReLU |  |  |  |  |  |
| 20 | inception_3a-5x5 | Convolution | 12×12×32 | Weights | 7×7×3×64 | 12832 |
|  | 32 5x5x16 convolutions with stride [1 1] and padding [2 2 2 2] |  |  | Bias | 1×1×32 |  |
| 21 | inception_3a-relu_5x5 | ReLU | 12×12×32 | - |  | 0 |
|  | ReLU |  |  |  |  |  |
| 22 | inception_3a-pool | Max Pooling | 12×12×192 | - |  | 0 |
|  | 3x3 max pooling with stride [1 1] and padding [1 1 1 1] |  |  |  |  |  |
| 23 | inception_3a-pool_proj | Convolution | 12×12×32 | Weights | 1×1×192×32 | 6176 |
|  | 32 1x1x192 convolutions with stride [1 1] and padding [0 0 0 0] |  |  | Bias | 1×1×32 |  |
| 24 | inception_3a-relu_pool_proj | ReLU | 12×12×32 | - |  | 0 |
|  | ReLU |  |  |  |  |  |
| 25 | inception_3a-output | Depth concatenation | 12×12×256 | - | Learnables | 0 |
|  | Depth concatenation of 4 inputs |  |  |  |  |  |
| 26 | inception_3b-1x1 | Convolution | 12×12×128 | Weights | 1×1×256×128 | 32896 |
|  | 128 1x1x256 convolutions with stride [1 1] and padding [0 0 0 0] |  |  | Bias | 1×1×128 |  |
| 27 | inception_3b-relu_1x1 | ReLU | 12×12×128 | - |  | 0 |
|  | ReLU |  |  |  |  |  |
| 28 | inception_3b-3x3_reduce | Convolution | 12×12×128 | Weights | 1×1×256×128 | 32896 |
|  | 128 1x1x256 convolutions with stride [1 1] and padding [0 0 0 0] |  |  | Bias | 1×1×128 |  |
| 29 | inception_3b-relu_3x3_reduce | ReLU | 12×12×128 | - |  | 0 |
|  | ReLU |  |  |  |  |  |
| 30 | inception_3b-3x3 | Convolution | 12×12×192 | Weights | 3×3×128×192 | 221376 |
|  | 192 3x3x128 convolutions with stride [1 1] and padding [1 1 1 1] |  |  | Bias | 1×1×192 |  |
| 31 | inception_3b-relu_3x3 | ReLU | 12×12×192 | - |  | 0 |
|  | ReLU |  |  |  |  |  |
| 32 | inception_3b-5x5_reduce | Convolution | 12×12×32 | Weights | 1×1×256×32 | 8224 |
|  | 32 1x1x256 convolutions with stride [1 1] and padding [0 0 0 0] |  |  | Bias | 1×1×32 |  |
| 33 | inception_3b-relu_5x5_reduce | ReLU | 12×12×32 | - |  | 0 |
|  | ReLU |  |  |  |  |  |
| 34 | inception_3b-5x5 | Convolution | 12×12×96 | Weights | 5×5×32×96 | 76896 |
|  | 96 5x5x32 convolutions with stride [1 1] and padding [2 2 2 2] |  |  | Bias | 1×1×96 |  |
| 35 | inception_3b-relu_5x5 | ReLU | 12×12×96 | - |  | 0 |
|  | ReLU |  |  |  |  |  |
| 36 | inception_3b-pool | Max Pooling | 12×12×256 | - |  | 0 |
|  | 3x3 max pooling with stride [1 1] and padding [1 1 1 1] |  |  |  |  |  |
| 37 | inception_3b-pool_proj | Convolution | 12×12×64 | Weights | 1×1×256×64 | 16448 |
|  | 64 1x1x256 convolutions with stride [1 1] and padding [0 0 0 0] |  |  | Bias | 1×1×64 |  |
| 38 | inception_3b-relu_pool_proj | ReLU | 12×12×64 | - |  | 0 |
|  | ReLU |  |  |  |  |  |
| 39 | inception_3b-output | Depth concatenation | 12×12×480 | - |  | 0 |
|  | Depth concatenation of 4 inputs |  |  |  |  |  |
| 40 | pool3-3x3_s2 | Max Pooling | 6×6×480 | - |  | 0 |
|  | 3x3 max pooling with stride [2 2] and padding [0 1 0 1] |  |  |  |  |  |
| 41 | inception_4a-1x1 | Convolution | 6×6×192 | Weights | 1×1×48 ×192 | 92352 |
|  | 192 1x1x480 convolutions with stride [1 1] and padding [0 0 0 0] |  |  | Bias | 1×1×192 |  |
| 42 | inception_4a-relu_1x1 | ReLU | 6×6×192 | - |  | 0 |
|  | ReLU |  |  |  |  |  |
| 43 | inception_4a-3x3_reduce | Convolution | 6×6×96 | Weights | 1×1×48 ×96 | 46176 |
|  | 96 1x1x480 convolutions with stride [1 1] and padding [0 0 0 0] |  |  | Bias | 1×1×96 |  |
| 44 | inception_4a-relu_3x3_reduce | ReLU | 6×6×96 | - |  | 0 |
|  | ReLU |  |  |  |  |  |
| 45 | inception_4a-3x3 | Convolution | 6×6×208 | Weights | 3×3×96×2 8 | 179920 |
|  | 208 3x3x96 convolutions with stride [1 1] and padding [1 1 1 1] |  |  | Bias | 1×1×2 8 |  |
| 46 | inception_4a-relu_3x3 | ReLU | 6×6×208 | - |  | 0 |
|  | ReLU |  |  |  |  |  |
| 47 | inception_4a-5x5_reduce | Convolution | 6×6×16 | Weights | 1×1×48 ×16 | 7696 |
|  | 16 1x1x480 convolutions with stride [1 1] and padding [0 0 0 0] |  |  | Bias | 1×1×16 |  |
| 48 | inception_4a-relu_5x5_reduce | ReLU | 6×6×16 | - |  | 0 |
|  | ReLU |  |  |  |  |  |
| 49 | inception_4a-5x5 | Convolution | 6×6×48 | Weights | 5×5×16×48 | 19248 |
|  | 48 5x5x16 convolutions with stride [1 1] and padding [2 2 2 2] |  |  | Bias | 1×1×48 |  |
| 50 | inception_4a-relu_5x5 | ReLU | 6×6×48 | - |  | 0 |
|  | ReLU |  |  |  |  |  |
| 51 | inception_4a-pool | Max Pooling | 6×6×480 | - |  | 0 |
|  | 3x3 max pooling with stride [1 1] and padding [1 1 1 1] |  |  |  |  |  |
| 52 | inception_4a-pool_proj | Convolution | 6×6×64 | Weights | 1×1×48 ×64 | 30784 |
|  | 64 1x1x480 convolutions with stride [1 1] and padding [0 0 0 0] |  |  | Bias | 1×1×64 |  |
| 53 | inception_4a-relu_pool_proj | ReLU | 6×6×64 | - |  | 0 |
|  | ReLU |  |  |  |  |  |
| 54 | inception_4a-output | Depth concatenation | 6×6×512 | - |  | 0 |
|  | Depth concatenation of 4 inputs |  |  |  |  |  |
| 55 | inception_4b-1x1 | Convolution | 6×6×160 | Weights | 1×1×512×16 | 82080 |
|  | 160 1x1x512 convolutions with stride [1 1] and padding [0 0 0 0] |  |  | Bias | 1×1×16 |  |
| 56 | inception_4b-relu_1x1 | ReLU | 6×6×160 | - |  | 0 |
|  | ReLU |  |  |  |  |  |
| 57 | inception_4b-3x3_reduce | Convolution | 6×6×112 | Weights | 1×1×512×112 | 57456 |
|  | 112 1x1x512 convolutions with stride [1 1] and padding [0 0 0 0] |  |  | Bias | 1×1×112 |  |
| 58 | inception_4b-relu_3x3_reduce | ReLU | 6×6×112 | - |  | 0 |
|  | ReLU |  |  |  |  |  |
| 59 | inception_4b-3x3 | Convolution | 6×6×224 | Weights | 3×3×112×224 | 226016 |
|  | 224 3x3x112 convolutions with stride [1 1] and padding [1 1 1 1] |  |  | Bias | 1×1×224 |  |
| 60 | inception_4b-relu_3x3 | ReLU | 6×6×224 | - |  | 0 |
|  | ReLU |  |  |  |  |  |
| 61 | inception_4b-5x5_reduce | Convolution | 6×6×24 | Weights | 1×1×512×24 | 12312 |
|  | 24 1x1x512 convolutions with stride [1 1] and padding [0 0 0 0] |  |  | Bias | 1×1×24 |  |
| 62 | inception_4b-relu_5x5_reduce | ReLU | 6×6×24 | - |  | 0 |
|  | ReLU |  |  |  |  |  |
| 63 | inception_4b-5x5 | Convolution | 6×6×64 | Weights | 5×5×24×64 | 38464 |
|  | 64 5x5x24 convolutions with stride [1 1] and padding [2 2 2 2] |  |  | Bias | 1×1×64 |  |
| 64 | inception_4b-relu_5x5 | ReLU | 6×6×64 | - |  | 0 |
|  | ReLU |  |  |  |  |  |
| 65 | inception_4b-pool | Max Pooling | 6×6×512 | - |  | 0 |
|  | 3x3 max pooling with stride [1 1] and padding [1 1 1 1] |  |  |  |  |  |
| 66 | inception_4b-pool_proj | Convolution | 6×6×64 | Weights | 1×1×512×64 | 32832 |
|  | 64 1x1x512 convolutions with stride [1 1] and padding [0 0 0 0] |  |  | Bias | 1×1×64 |  |
| 67 | inception_4b-relu_pool_proj | ReLU | 6×6×64 | - |  | 0 |
|  | ReLU |  |  |  |  |  |
| 68 | inception_4b-output | Depth concatenation | 6×6×512 | - |  | 0 |
|  | Depth concatenation of 4 inputs |  |  |  |  |  |
| 69 | inception_4c-1x1 | Convolution | 6×6×128 | Weights | 1×1×512×128 | 65664 |
|  | 128 1x1x512 convolutions with stride [1 1] and padding [0 0 0 0] |  |  | Bias | 1×1×128 |  |
| 70 | inception_4c-relu_1x1 | ReLU | 6×6×128 | - |  | 0 |
|  | ReLU |  |  |  |  |  |
| 71 | inception_4c-3x3_reduce | Convolution | 6×6×128 | Weights | 1×1×512×128 | 65664 |
|  | 128 1x1x512 convolutions with stride [1 1] and padding [0 0 0 0] |  |  | Bias | 1×1×128 |  |
| 72 | inception_4c-relu_3x3_reduce | ReLU | 6×6×128 | - |  | 0 |
|  | ReLU |  |  |  |  |  |
| 73 | inception_4c-3x3 | Convolution | 6×6×256 | Weights | 3×3×128×256 | 295168 |
|  | 256 3x3x128 convolutions with stride [1 1] and padding [1 1 1 1] |  |  | Bias | 1×1×256 |  |
| 74 | inception_4c-relu_3x3 | ReLU | 6×6×256 | - |  | 0 |
|  | ReLU |  |  |  |  |  |
| 75 | inception_4c-5x5_reduce | Convolution | 6×6×24 | Weights | 1×1×512×24 | 12312 |
|  | 24 1x1x512 convolutions with stride [1 1] and padding [0 0 0 0] |  |  | Bias | 1×1×24 |  |
| 76 | inception_4c-relu_5x5_reduce | ReLU | 6×6×24 | - |  | 0 |
|  | ReLU |  |  |  |  |  |
| 77 | inception_4c-5x5 | Convolution | 6×6×64 | Weights | 5×5×24×64 | 38464 |
|  | 64 5x5x24 convolutions with stride [1 1] and padding [2 2 2 2] |  |  | Bias | 1×1×64 |  |
| 78 | inception_4c-relu_5x5 | ReLU | 6×6×64 | - |  | 0 |
|  | ReLU |  |  |  |  |  |
| 79 | inception_4c-pool | Max Pooling | 6×6×512 | - |  | 0 |
|  | 3x3 max pooling with stride [1 1] and padding [1 1 1 1] |  |  |  |  |  |
| 80 | inception_4c-pool_proj | Convolution | 6×6×64 | Weights | 1×1×512×64 | 32832 |
|  | 64 1x1x512 convolutions with stride [1 1] and padding [0 0 0 0] |  |  | Bias | 1×1×64 |  |
| 81 | inception_4c-relu_pool_proj | ReLU | 6×6×64 | - |  | 0 |
|  | ReLU |  |  |  |  |  |
| 82 | inception_4c-output | Depth concatenation | 6×6×512 | - |  | 0 |
|  | Depth concatenation of 4 inputs |  |  |  |  |  |
| 83 | inception_4d-1x1 | Convolution | 6×6×112 | Weights | 1×1×512×112 | 57456 |
|  | 112 1x1x512 convolutions with stride [1 1] and padding [0 0 0 0] |  |  | Bias | 1×1×112 |  |
| 84 | inception_4d-relu_1x1 | ReLU | 6×6×112 | - |  | 0 |
|  | ReLU |  |  |  |  |  |
| 85 | inception_4d-3x3_reduce | Convolution | 6×6×144 | Weights | 1×1×512×144 | 73872 |
|  | 144 1x1x512 convolutions with stride [1 1] and padding [0 0 0 0] |  |  | Bias | 1×1×144 |  |
| 86 | inception_4d-relu_3x3_reduce | ReLU | 6×6×144 | - |  | 0 |
|  | ReLU |  |  |  |  |  |
| 87 | inception_4d-3x3 | Convolution | 6×6×288 | Weights | 3×3×144×288 | 373536 |
|  | 288 3x3x144 convolutions with stride [1 1] and padding [1 1 1 1] |  |  | Bias | 1×1×288 |  |
| 88 | inception_4d-relu_3x3 | ReLU | 6×6×288 | - |  | 0 |
|  | ReLU |  |  |  |  |  |
| 89 | inception_4d-5x5_reduce | Convolution | 6×6×32 | Weights | 1×1×512×32 | 16416 |
|  | 32 1x1x512 convolutions with stride [1 1] and padding [0 0 0 0] |  |  | Bias | 1×1×32 |  |
| 90 | inception_4d-relu_5x5_reduce | ReLU | 6×6×32 | - |  | 0 |
|  | ReLU |  |  |  |  |  |
| 91 | inception_4d-5x5 | Convolution | 6×6×64 | Weights | 5×5×32×64 | 51264 |
|  | 64 5x5x32 convolutions with stride [1 1] and padding [2 2 2 2] |  |  | Bias | 1×1×64 |  |
| 92 | inception_4d-relu_5x5 | ReLU | 6×6×64 | - |  | 0 |
|  | ReLU |  |  |  |  |  |
| 93 | inception_4d-pool | Max Pooling | 6×6×512 | - |  | 0 |
|  | 3x3 max pooling with stride [1 1] and padding [1 1 1 1] |  |  |  |  |  |
| 94 | inception_4d-pool_proj | Convolution | 6×6×64 | Weights | 1×1×512×64 | 32832 |
|  | 64 1x1x512 convolutions with stride [1 1] and padding [0 0 0 0] |  |  | Bias | 1×1×64 |  |
| 95 | inception_4d-relu_pool_proj | ReLU | 6×6×64 | - |  | 0 |
|  | ReLU |  |  |  |  |  |
| 96 | inception_4d-output | Depth concatenation | 6×6×528 | - |  | 0 |
|  | Depth concatenation of 4 inputs |  |  |  |  |  |
| 97 | inception_4e-1x1 | Convolution | 6×6×256 | Weights | 1×1×528×256 | 135424 |
|  | 256 1x1x528 convolutions with stride [1 1] and padding [0 0 0 0] |  |  | Bias | 1×1×256 |  |
| 98 | inception_4e-relu_1x1 | ReLU | 6×6×256 | - |  | 0 |
|  | ReLU |  |  |  |  |  |
| 99 | inception_4e-3x3_reduce | Convolution | 6×6×160 | Weights | 1×1×528×16 | 84640 |
|  | 160 1x1x528 convolutions with stride [1 1] and padding [0 0 0 0] |  |  | Bias | 1×1×16 |  |
| 100 | inception_4e-relu_3x3_reduce | ReLU | 6×6×160 | - |  | 0 |
|  | ReLU |  |  |  |  |  |
| 101 | inception_4e-3x3 | Convolution | 6×6×320 | Weights | 3×3×16 ×32 | 461120 |
|  | 320 3x3x160 convolutions with stride [1 1] and padding [1 1 1 1] |  |  | Bias | 1×1×32 |  |
| 102 | inception_4e-relu_3x3 | ReLU | 6×6×320 | - |  | 0 |
|  | ReLU |  |  |  |  |  |
| 103 | inception_4e-5x5_reduce | Convolution | 6×6×32 | Weights | 1×1×528×32 | 16928 |
|  | 32 1x1x528 convolutions with stride [1 1] and padding [0 0 0 0] |  |  | Bias | 1×1×32 |  |
| 104 | inception_4e-relu_5x5_reduce | ReLU | 6×6×32 | - |  | 0 |
|  | ReLU |  |  |  |  |  |
| 105 | inception_4e-5x5 | Convolution | 6×6×128 | Weights | 5×5×32×128 | 102528 |
|  | 128 5x5x32 convolutions with stride [1 1] and padding [2 2 2 2] |  |  | Bias | 1×1×128 |  |
| 106 | inception_4e-relu_5x5 | ReLU | 6×6×128 | - |  | 0 |
|  | ReLU |  |  |  |  |  |
| 107 | inception_4e-pool | Max Pooling | 6×6×528 | - |  | 0 |
|  | 3x3 max pooling with stride [1 1] and padding [1 1 1 1] |  |  |  |  |  |
| 108 | inception_4e-pool_proj | Convolution | 6×6×128 | Weights | 1×1×528×128 | 67712 |
|  | 128 1x1x528 convolutions with stride [1 1] and padding [0 0 0 0] |  |  | Bias | 1×1×128 |  |
| 109 | inception_4e-relu_pool_proj | ReLU | 6×6×128 | - |  | 0 |
|  | ReLU |  |  |  |  |  |
| 110 | inception_4e-output | Depth concatenation | 6×6×832 | - |  | 0 |
|  | Depth concatenation of 4 inputs |  |  |  |  |  |
| 111 | pool4-3x3_s2 | Max Pooling | 3×3×832 | - |  | 0 |
|  | 3x3 max pooling with stride [2 2] and padding [0 1 0 1] |  |  |  |  |  |
| 112 | inception_5a-1x1 | Convolution | 3×3×256 | Weights | 1×1×832×256 | 213248 |
|  | 256 1x1x832 convolutions with stride [1 1] and padding [0 0 0 0] |  |  | Bias | 1×1×256 |  |
| 113 | inception_5a-relu_1x1 | ReLU | 3×3×256 | - |  | 0 |
|  | ReLU |  |  |  |  |  |
| 114 | inception_5a-3x3_reduce | Convolution | 3×3×160 | Weights | 1×1×832×16 | 133280 |
|  | 160 1x1x832 convolutions with stride [1 1] and padding [0 0 0 0] |  |  | Bias | 1×1×16 |  |
| 115 | inception_5a-relu_3x3_reduce | ReLU | 3×3×160 | - |  | 0 |
|  | ReLU |  |  |  |  |  |
| 116 | inception_5a-3x3 | Convolution | 3×3×320 | Weights | 3×3×16 ×32 | 461120 |
|  | 320 3x3x160 convolutions with stride [1 1] and padding [1 1 1 1] |  |  | Bias | 1×1×32 |  |
| 117 | inception_5a-relu_3x3 | ReLU | 3×3×320 | - |  | 0 |
|  | ReLU |  |  |  |  |  |
| 118 | inception_5a-5x5_reduce | Convolution | 3×3×32 | Weights | 1×1×832×32 | 26656 |
|  | 32 1x1x832 convolutions with stride [1 1] and padding [0 0 0 0] |  |  | Bias | 1×1×32 |  |
| 119 | inception_5a-relu_5x5_reduce | ReLU | 3×3×32 | - |  | 0 |
|  | ReLU |  |  |  |  |  |
| 120 | inception_5a-5x5 | Convolution | 3×3×128 | Weights | 5×5×32×128 | 102528 |
|  | 128 5x5x32 convolutions with stride [1 1] and padding [2 2 2 2] |  |  | Bias | 1×1×128 |  |
| 121 | inception_5a-relu_5x5 | ReLU | 3×3×128 | - |  | 0 |
|  | ReLU |  |  |  |  |  |
| 122 | inception_5a-pool | Max Pooling | 3×3×832 | - |  | 0 |
|  | 3x3 max pooling with stride [1 1] and padding [1 1 1 1] |  |  |  |  |  |
| 123 | inception_5a-pool_proj | Convolution | 3×3×128 | Weights | 1×1×832×128 | 106624 |
|  | 128 1x1x832 convolutions with stride [1 1] and padding [0 0 0 0] |  |  | Bias | 1×1×128 |  |
| 124 | inception_5a-relu_pool_proj | ReLU | 3×3×128 | - |  | 0 |
|  | ReLU |  |  |  |  |  |
| 125 | inception_5a-output | Depth concatenation | 3×3×832 | - |  | 0 |
|  | Depth concatenation of 4 inputs |  |  |  |  |  |
| 126 | inception_5b-1x1 | Convolution | 3×3×384 | Weights | 1×1×832×384 | 319872 |
|  | 384 1x1x832 convolutions with stride [1 1] and padding [0 0 0 0] |  |  | Bias | 1×1×384 |  |
| 127 | inception_5b-relu_1x1 | ReLU | 3×3×384 | - |  | 0 |
|  | ReLU |  |  |  |  |  |
| 128 | inception_5b-3x3_reduce | Convolution | 3×3×192 | Weights | 1×1×832×192 | 159936 |
|  | 192 1x1x832 convolutions with stride [1 1] and padding [0 0 0 0] |  |  | Bias | 1×1×192 |  |
| 129 | inception_5b-relu_3x3_reduce | ReLU | 3×3×192 | - |  | 0 |
|  | ReLU |  |  |  |  |  |
| 130 | inception_5b-3x3 | Convolution | 3×3×384 | Weights | 3×3×192×384 | 663936 |
|  | 384 3x3x192 convolutions with stride [1 1] and padding [1 1 1 1] |  |  | Bias | 1×1×384 |  |
| 131 | inception_5b-relu_3x3 | ReLU | 3×3×384 | - |  | 0 |
|  | ReLU |  |  |  |  |  |
| 132 | inception_5b-5x5_reduce | Convolution | 3×3×48 | Weights | 1×1×832×48 | 39984 |
|  | 48 1x1x832 convolutions with stride [1 1] and padding [0 0 0 0] |  |  | Bias | 1×1×48 |  |
| 133 | inception_5b-relu_5x5_reduce | ReLU | 3×3×48 | - |  | 0 |
|  | ReLU |  |  |  |  |  |
| 134 | inception_5b-5x5 | Convolution | 3×3×128 | Weights | 5×5×48×128 | 153728 |
|  | 128 5x5x48 convolutions with stride [1 1] and padding [2 2 2 2] |  |  | Bias | 1×1×128 |  |
| 135 | inception_5b-relu_5x5 | ReLU | 3×3×128 | - |  | 0 |
|  | ReLU |  |  |  |  |  |
| 136 | inception_5b-pool | Max Pooling | 3×3×832 | - |  | 0 |
|  | 3x3 max pooling with stride [1 1] and padding [1 1 1 1] |  |  |  |  |  |
| 137 | inception_5b-pool_proj | Convolution | 3×3×128 | Weights | 1×1×832×128 | 106624 |
|  | 128 1x1x832 convolutions with stride [1 1] and padding [0 0 0 0] |  |  | Bias | 1×1×128 |  |
| 138 | inception_5b-relu_pool_proj | ReLU | 3×3×128 | - |  | 0 |
|  | ReLU |  |  |  |  |  |
| 139 | inception_5b-output | Depth concatenation | 3×3×1024 | - |  | 0 |
|  | Depth concatenation of 4 inputs |  |  |  |  |  |
| 140 | pool5-7x7_s1 | Average Pooling | 3×3×1024 | - |  | 0 |
|  | 1x1 average pooling with stride [1 1] and padding [0 0 0 0] |  |  |  |  |  |
| 141 | pool5-drop_7x7_s1 | Dropout | 3×3×1024 | - |  | 0 |
|  | 40% dropout |  |  |  |  |  |
| 142 | loss3-classifier | Fully Connected | 1×1×5 | Weights | 5×9216 | 46085 |
|  | 5 fully connected layer |  |  | Bias | 5×1 |  |
| 143 | prob | Softmax | 1×1×5 | - |  | 0 |
|  | softmax |  |  |  |  |  |
| 144 | output | Classification Output | - | - |  | 0 |
|  | crossentropyex with 'bt474' and 4 other classes |  |  |  |  |  |

## Supplementary Data 6. ClassNet^DC^ network details.

Please find **Supplementary Figure 5; GoogleNet** for graphic visualization of layer connections.

|  | Name | Type | Activations | Learnables |  | TotalLearnables |
| --- | --- | --- | --- | --- | --- | --- |
| 1 | data | Image Input | 101×101×3 | - |  | 0 |
|  | 101x101x3 images with 'zerocenter' normalization |  |  |  |  |  |
| 2 | conv1-7x7_s2 | Convolution | 51×51×64 | Weights | 7×7×3×64 | 9472 |
|  | 64 7x7x3 convolutions with stride [2 2] and padding [3 3 3 3] |  |  | Bias | 1×1×64 |  |
| 3 | conv1-relu_7x7 | ReLU | 51×51×64 | - |  | 0 |
|  | ReLU |  |  |  |  |  |
| 4 | pool1-3x3_s2 | Max Pooling | 25×25×64 | - |  | 0 |
|  | 3x3 max pooling with stride [2 2] and padding [0 1 0 1] |  |  |  |  |  |
| 5 | pool1-norm1 | Cross Channel Normalization | 25×25×64 | - |  | 0 |
|  | cross channel normalization with 5 channels per element |  |  |  |  |  |
| 6 | conv2-3x3_reduce | Convolution | 25×25×64 | Weights | 1×1×64×64 | 4160 |
|  | 64 1x1x64 convolutions with stride [1 1] and padding [0 0 0 0] |  |  | Bias | 1×1×64 |  |
| 7 | conv2-relu_3x3_reduce | ReLU | 25×25×64 | - |  | 0 |
|  | ReLU |  |  |  |  |  |
| 8 | conv2-3x3 | Convolution | 25×25×192 | Weights | 3×3×64×192 | 110784 |
|  | 192 3x3x64 convolutions with stride [1 1] and padding [1 1 1 1] |  |  | Bias | 1×1×192 |  |
| 9 | conv2-relu_3x3 | ReLU | 25×25×192 | - |  | 0 |
|  | ReLU |  |  |  |  |  |
| 10 | conv2-norm2 | Cross Channel Normalization | 25×25×192 | - |  | 0 |
|  | cross channel normalization with 5 channels per element |  |  |  |  |  |
| 11 | pool2-3x3_s2 | Max Pooling | 12×12×192 | - |  | 0 |
|  | 3x3 max pooling with stride [2 2] and padding [0 1 0 1] |  |  |  |  |  |
| 12 | inception_3a-1x1 | Convolution | 12×12×64 | Weights | 1×1×192×64 | 12352 |
|  | 64 1x1x192 convolutions with stride [1 1] and padding [0 0 0 0] |  |  | Bias | 1×1×64 |  |
| 13 | inception_3a-relu_1x1 | ReLU | 12×12×64 | - |  | 0 |
|  | ReLU |  |  |  |  |  |
| 14 | inception_3a-3x3_reduce | Convolution | 12×12×96 | Weights | 1×1×192×96 | 18528 |
|  | 96 1x1x192 convolutions with stride [1 1] and padding [0 0 0 0] |  |  | Bias | 1×1×96 |  |
| 15 | inception_3a-relu_3x3_reduce | ReLU | 12×12×96 | - |  | 0 |
|  | ReLU |  |  |  |  |  |
| 16 | inception_3a-3x3 | Convolution | 12×12×128 | Weights | 3×3×96×128 | 110720 |
|  | 128 3x3x96 convolutions with stride [1 1] and padding [1 1 1 1] |  |  | Bias | 1×1×128 |  |
| 17 | inception_3a-relu_3x3 | ReLU | 12×12×128 | - |  | 0 |
|  | ReLU |  |  |  |  |  |
| 18 | inception_3a-5x5_reduce | Convolution | 12×12×16 | Weights | 1×1×192×16 | 3088 |
|  | 16 1x1x192 convolutions with stride [1 1] and padding [0 0 0 0] |  |  | Bias | 1×1×16 |  |
| 19 | inception_3a-relu_5x5_reduce | ReLU | 12×12×16 | - |  | 0 |
|  | ReLU |  |  |  |  |  |
| 20 | inception_3a-5x5 | Convolution | 12×12×32 | Weights | 5×5×16×32 | 12832 |
|  | 32 5x5x16 convolutions with stride [1 1] and padding [2 2 2 2] |  |  | Bias | 1×1×32 |  |
| 21 | inception_3a-relu_5x5 | ReLU | 12×12×32 | - |  | 0 |
|  | ReLU |  |  |  |  |  |
| 22 | inception_3a-pool | Max Pooling | 12×12×192 | - |  | 0 |
|  | 3x3 max pooling with stride [1 1] and padding [1 1 1 1] |  |  |  |  |  |
| 23 | inception_3a-pool_proj | Convolution | 12×12×32 | Weights | 1×1×192×32 | 6176 |
|  | 32 1x1x192 convolutions with stride [1 1] and padding [0 0 0 0] |  |  | Bias | 1×1×32 |  |
| 24 | inception_3a-relu_pool_proj | ReLU | 12×12×32 | - |  | 0 |
|  | ReLU |  |  |  |  |  |
| 25 | inception_3a-output | Depth concatenation | 12×12×256 | - |  | 0 |
|  | Depth concatenation of 4 inputs |  |  |  |  |  |
| 26 | inception_3b-1x1 | Convolution | 12×12×128 | Weights | 1×1×256×128 | 32896 |
|  | 128 1x1x256 convolutions with stride [1 1] and padding [0 0 0 0] |  |  | Bias | 1×1×128 |  |
| 27 | inception_3b-relu_1x1 | ReLU | 12×12×128 | - |  | 0 |
|  | ReLU |  |  |  |  |  |
| 28 | inception_3b-3x3_reduce | Convolution | 12×12×128 | Weights | 1×1×256×128 | 32896 |
|  | 128 1x1x256 convolutions with stride [1 1] and padding [0 0 0 0] |  |  | Bias | 1×1×128 |  |
| 29 | inception_3b-relu_3x3_reduce | ReLU | 12×12×128 | - |  | 0 |
|  | ReLU |  |  |  |  |  |
| 30 | inception_3b-3x3 | Convolution | 12×12×192 | Weights | 3×3×128×192 | 221376 |
|  | 192 3x3x128 convolutions with stride [1 1] and padding [1 1 1 1] |  |  | Bias | 1×1×192 |  |
| 31 | inception_3b-relu_3x3 | ReLU | 12×12×192 | - |  | 0 |
|  | ReLU |  |  |  |  |  |
| 32 | inception_3b-5x5_reduce | Convolution | 12×12×32 | Weights | 1×1×256×32 | 8224 |
|  | 32 1x1x256 convolutions with stride [1 1] and padding [0 0 0 0] |  |  | Bias | 1×1×32 |  |
| 33 | inception_3b-relu_5x5_reduce | ReLU | 12×12×32 | - |  | 0 |
|  | ReLU |  |  |  |  |  |
| 34 | inception_3b-5x5 | Convolution | 12×12×96 | Weights | 5×5×32×96 | 76896 |
|  | 96 5x5x32 convolutions with stride [1 1] and padding [2 2 2 2] |  |  | Bias | 1×1×96 |  |
| 35 | inception_3b-relu_5x5 | ReLU | 12×12×96 | - |  | 0 |
|  | ReLU |  |  |  |  |  |
| 36 | inception_3b-pool | Max Pooling | 12×12×256 | - |  | 0 |
|  | 3x3 max pooling with stride [1 1] and padding [1 1 1 1] |  |  |  |  |  |
| 37 | inception_3b-pool_proj | Convolution | 12×12×64 | Weights | 1×1×256×64 | 16448 |
|  | 64 1x1x256 convolutions with stride [1 1] and padding [0 0 0 0] |  |  | Bias | 1×1×64 |  |
| 38 | inception_3b-relu_pool_proj | ReLU | 12×12×64 | - |  | 0 |
|  | ReLU |  |  |  |  |  |
| 39 | inception_3b-output | Depth concatenation | 12×12×480 | - |  | 0 |
|  | Depth concatenation of 4 inputs |  |  |  |  |  |
| 40 | pool3-3x3_s2 | Max Pooling | 6×6×480 | - |  | 0 |
|  | 3x3 max pooling with stride [2 2] and padding [0 1 0 1] |  |  |  |  |  |
| 41 | inception_4a-1x1 | Convolution | 6×6×192 | Weights | 1×1×48 ×192 | 92352 |
|  | 192 1x1x480 convolutions with stride [1 1] and padding [0 0 0 0] |  |  | Bias | 1×1×192 |  |
| 42 | inception_4a-relu_1x1 | ReLU | 6×6×192 | - |  | 0 |
|  | ReLU |  |  |  |  |  |
| 43 | inception_4a-3x3_reduce | Convolution | 6×6×96 | Weights | 1×1×48 ×96 | 46176 |
|  | 96 1x1x480 convolutions with stride [1 1] and padding [0 0 0 0] |  |  | Bias | 1×1×96 |  |
| 44 | inception_4a-relu_3x3_reduce | ReLU | 6×6×96 | - |  | 0 |
|  | ReLU |  |  |  |  |  |
| 45 | inception_4a-3x3 | Convolution | 6×6×208 | Weights | 3×3×96×2 8 | 179920 |
|  | 208 3x3x96 convolutions with stride [1 1] and padding [1 1 1 1] |  |  | Bias | 1×1×2 8 |  |
| 46 | inception_4a-relu_3x3 | ReLU | 6×6×208 | - |  | 0 |
|  | ReLU |  |  |  |  |  |
| 47 | inception_4a-5x5_reduce | Convolution | 6×6×16 | Weights | 1×1×48 ×16 | 7696 |
|  | 16 1x1x480 convolutions with stride [1 1] and padding [0 0 0 0] |  |  | Bias | 1×1×16 |  |
| 48 | inception_4a-relu_5x5_reduce | ReLU | 6×6×16 | - |  | 0 |
|  | ReLU |  |  |  |  |  |
| 49 | inception_4a-5x5 | Convolution | 6×6×48 | Weights | 5×5×16×48 | 19248 |
|  | 48 5x5x16 convolutions with stride [1 1] and padding [2 2 2 2] |  |  | Bias | 1×1×48 |  |
| 50 | inception_4a-relu_5x5 | ReLU | 6×6×48 | - |  | 0 |
|  | ReLU |  |  |  |  |  |
| 51 | inception_4a-pool | Max Pooling | 6×6×480 | - |  | 0 |
|  | 3x3 max pooling with stride [1 1] and padding [1 1 1 1] |  |  |  |  |  |
| 52 | inception_4a-pool_proj | Convolution | 6×6×64 | Weights | 1×1×48 ×64 | 30784 |
|  | 64 1x1x480 convolutions with stride [1 1] and padding [0 0 0 0] |  |  | Bias | 1×1×64 |  |
| 53 | inception_4a-relu_pool_proj | ReLU | 6×6×64 | - |  | 0 |
|  | ReLU |  |  |  |  |  |
| 54 | inception_4a-output | Depth concatenation | 6×6×512 | - |  | 0 |
|  | Depth concatenation of 4 inputs |  |  |  |  |  |
| 55 | inception_4b-1x1 | Convolution | 6×6×160 | Weights | 1×1×512×16 | 82080 |
|  | 160 1x1x512 convolutions with stride [1 1] and padding [0 0 0 0] |  |  | Bias | 1×1×16 |  |
| 56 | inception_4b-relu_1x1 | ReLU | 6×6×160 | - |  | 0 |
|  | ReLU |  |  |  |  |  |
| 57 | inception_4b-3x3_reduce | Convolution | 6×6×112 | Weights | 1×1×512×112 | 57456 |
|  | 112 1x1x512 convolutions with stride [1 1] and padding [0 0 0 0] |  |  | Bias | 1×1×112 |  |
| 58 | inception_4b-relu_3x3_reduce | ReLU | 6×6×112 | - |  | 0 |
|  | ReLU |  |  |  |  |  |
| 59 | inception_4b-3x3 | Convolution | 6×6×224 | Weights | 3×3×112×224 | 226016 |
|  | 224 3x3x112 convolutions with stride [1 1] and padding [1 1 1 1] |  |  | Bias | 1×1×224 |  |
| 60 | inception_4b-relu_3x3 | ReLU | 6×6×224 | - |  | 0 |
|  | ReLU |  |  |  |  |  |
| 61 | inception_4b-5x5_reduce | Convolution | 6×6×24 | Weights | 1×1×512×24 | 12312 |
|  | 24 1x1x512 convolutions with stride [1 1] and padding [0 0 0 0] |  |  | Bias | 1×1×24 |  |
| 62 | inception_4b-relu_5x5_reduce | ReLU | 6×6×24 | - |  | 0 |
|  | ReLU |  |  |  |  |  |
| 63 | inception_4b-5x5 | Convolution | 6×6×64 | Weights | 5×5×24×64 | 38464 |
|  | 64 5x5x24 convolutions with stride [1 1] and padding [2 2 2 2] |  |  | Bias | 1×1×64 |  |
| 64 | inception_4b-relu_5x5 | ReLU | 6×6×64 | - |  | 0 |
|  | ReLU |  |  |  |  |  |
| 65 | inception_4b-pool | Max Pooling | 6×6×512 | - |  | 0 |
|  | 3x3 max pooling with stride [1 1] and padding [1 1 1 1] |  |  |  |  |  |
| 66 | inception_4b-pool_proj | Convolution | 6×6×64 | Weights | 1×1×512×64 | 32832 |
|  | 64 1x1x512 convolutions with stride [1 1] and padding [0 0 0 0] |  |  | Bias | 1×1×64 |  |
| 67 | inception_4b-relu_pool_proj | ReLU | 6×6×64 | - |  | 0 |
|  | ReLU |  |  |  |  |  |
| 68 | inception_4b-output | Depth concatenation | 6×6×512 | - |  | 0 |
|  | Depth concatenation of 4 inputs |  |  |  |  |  |
| 69 | inception_4c-1x1 | Convolution | 6×6×128 | Weights | 1×1×512×128 | 65664 |
|  | 128 1x1x512 convolutions with stride [1 1] and padding [0 0 0 0] |  |  | Bias | 1×1×128 |  |
| 70 | inception_4c-relu_1x1 | ReLU | 6×6×128 | - |  | 0 |
|  | ReLU |  |  |  |  |  |
| 71 | inception_4c-3x3_reduce | Convolution | 6×6×128 | Weights | 1×1×512×128 | 65664 |
|  | 128 1x1x512 convolutions with stride [1 1] and padding [0 0 0 0] |  |  | Bias | 1×1×128 |  |
| 72 | inception_4c-relu_3x3_reduce | ReLU | 6×6×128 | - |  | 0 |
|  | ReLU |  |  |  |  |  |
| 73 | inception_4c-3x3 | Convolution | 6×6×256 | Weights | 3×3×128×256 | 295168 |
|  | 256 3x3x128 convolutions with stride [1 1] and padding [1 1 1 1] |  |  | Bias | 1×1×256 |  |
| 74 | inception_4c-relu_3x3 | ReLU | 6×6×256 | - |  | 0 |
|  | ReLU |  |  |  |  |  |
| 75 | inception_4c-5x5_reduce | Convolution | 6×6×24 | Weights | 1×1×512×24 | 12312 |
|  | 24 1x1x512 convolutions with stride [1 1] and padding [0 0 0 0] |  |  | Bias | 1×1×24 |  |
| 76 | inception_4c-relu_5x5_reduce | ReLU | 6×6×24 | - |  | 0 |
|  | ReLU |  |  |  |  |  |
| 77 | inception_4c-5x5 | Convolution | 6×6×64 | Weights | 5×5×24×64 | 38464 |
|  | 64 5x5x24 convolutions with stride [1 1] and padding [2 2 2 2] |  |  | Bias | 1×1×64 |  |
| 78 | inception_4c-relu_5x5 | ReLU | 6×6×64 | - |  | 0 |
|  | ReLU |  |  |  |  |  |
| 79 | inception_4c-pool | Max Pooling | 6×6×512 | - |  | 0 |
|  | 3x3 max pooling with stride [1 1] and padding [1 1 1 1] |  |  |  |  |  |
| 80 | inception_4c-pool_proj | Convolution | 6×6×64 | Weights | 1×1×512×64 | 32832 |
|  | 64 1x1x512 convolutions with stride [1 1] and padding [0 0 0 0] |  |  | Bias | 1×1×64 |  |
| 81 | inception_4c-relu_pool_proj | ReLU | 6×6×64 | - |  | 0 |
|  | ReLU |  |  |  |  |  |
| 82 | inception_4c-output | Depth concatenation | 6×6×512 | - |  | 0 |
|  | Depth concatenation of 4 inputs |  |  |  |  |  |
| 83 | inception_4d-1x1 | Convolution | 6×6×112 | Weights | 1×1×512×112 | 57456 |
|  | 112 1x1x512 convolutions with stride [1 1] and padding [0 0 0 0] |  |  | Bias | 1×1×112 |  |
| 84 | inception_4d-relu_1x1 | ReLU | 6×6×112 | - |  | 0 |
|  | ReLU |  |  |  |  |  |
| 85 | inception_4d-3x3_reduce | Convolution | 6×6×144 | Weights | 1×1×512×144 | 73872 |
|  | 144 1x1x512 convolutions with stride [1 1] and padding [0 0 0 0] |  |  | Bias | 1×1×144 |  |
| 86 | inception_4d-relu_3x3_reduce | ReLU | 6×6×144 | - |  | 0 |
|  | ReLU |  |  |  |  |  |
| 87 | inception_4d-3x3 | Convolution | 6×6×288 | Weights | 3×3×144×288 | 373536 |
|  | 288 3x3x144 convolutions with stride [1 1] and padding [1 1 1 1] |  |  | Bias | 1×1×288 |  |
| 88 | inception_4d-relu_3x3 | ReLU | 6×6×288 | - |  | 0 |
|  | ReLU |  |  |  |  |  |
| 89 | inception_4d-5x5_reduce | Convolution | 6×6×32 | Weights | 1×1×512×32 | 16416 |
|  | 32 1x1x512 convolutions with stride [1 1] and padding [0 0 0 0] |  |  | Bias | 1×1×32 |  |
| 90 | inception_4d-relu_5x5_reduce | ReLU | 6×6×32 | - |  | 0 |
|  | ReLU |  |  |  |  |  |
| 91 | inception_4d-5x5 | Convolution | 6×6×64 | Weights | 5×5×32×64 | 51264 |
|  | 64 5x5x32 convolutions with stride [1 1] and padding [2 2 2 2] |  |  | Bias | 1×1×64 |  |
| 92 | inception_4d-relu_5x5 | ReLU | 6×6×64 | - |  | 0 |
|  | ReLU |  |  |  |  |  |
| 93 | inception_4d-pool | Max Pooling | 6×6×512 | - |  | 0 |
|  | 3x3 max pooling with stride [1 1] and padding [1 1 1 1] |  |  |  |  |  |
| 94 | inception_4d-pool_proj | Convolution | 6×6×64 | Weights | 1×1×512×64 | 32832 |
|  | 64 1x1x512 convolutions with stride [1 1] and padding [0 0 0 0] |  |  | Bias | 1×1×64 |  |
| 95 | inception_4d-relu_pool_proj | ReLU | 6×6×64 | - |  | 0 |
|  | ReLU |  |  |  |  |  |
| 96 | inception_4d-output | Depth concatenation | 6×6×528 | - |  | 0 |
|  | Depth concatenation of 4 inputs |  |  |  |  |  |
| 97 | inception_4e-1x1 | Convolution | 6×6×256 | Weights | 1×1×528×256 | 135424 |
|  | 256 1x1x528 convolutions with stride [1 1] and padding [0 0 0 0] |  |  | Bias | 1×1×256 |  |
| 98 | inception_4e-relu_1x1 | ReLU | 6×6×256 | - |  | 0 |
|  | ReLU |  |  |  |  |  |
| 99 | inception_4e-3x3_reduce | Convolution | 6×6×160 | Weights | 1×1×528×16 | 84640 |
|  | 160 1x1x528 convolutions with stride [1 1] and padding [0 0 0 0] |  |  | Bias | 1×1×16 |  |
| 100 | inception_4e-relu_3x3_reduce | ReLU | 6×6×160 | - |  | 0 |
|  | ReLU |  |  |  |  |  |
| 101 | inception_4e-3x3 | Convolution | 6×6×320 | Weights | 3×3×16 ×32 | 461120 |
|  | 320 3x3x160 convolutions with stride [1 1] and padding [1 1 1 1] |  |  | Bias | 1×1×32 |  |
| 102 | inception_4e-relu_3x3 | ReLU | 6×6×320 | - |  | 0 |
|  | ReLU |  |  |  |  |  |
| 103 | inception_4e-5x5_reduce | Convolution | 6×6×32 | Weights | 1×1×528×32 | 16928 |
|  | 32 1x1x528 convolutions with stride [1 1] and padding [0 0 0 0] |  |  | Bias | 1×1×32 |  |
| 104 | inception_4e-relu_5x5_reduce | ReLU | 6×6×32 | - |  | 0 |
|  | ReLU |  |  |  |  |  |
| 105 | inception_4e-5x5 | Convolution | 6×6×128 | Weights | 5×5×32×128 | 102528 |
|  | 128 5x5x32 convolutions with stride [1 1] and padding [2 2 2 2] |  |  | Bias | 1×1×128 |  |
| 106 | inception_4e-relu_5x5 | ReLU | 6×6×128 | - |  | 0 |
|  | ReLU |  |  |  |  |  |
| 107 | inception_4e-pool | Max Pooling | 6×6×528 | - |  | 0 |
|  | 3x3 max pooling with stride [1 1] and padding [1 1 1 1] |  |  |  |  |  |
| 108 | inception_4e-pool_proj | Convolution | 6×6×128 | Weights | 1×1×528×128 | 67712 |
|  | 128 1x1x528 convolutions with stride [1 1] and padding [0 0 0 0] |  |  | Bias | 1×1×128 |  |
| 109 | inception_4e-relu_pool_proj | ReLU | 6×6×128 | - |  | 0 |
|  | ReLU |  |  |  |  |  |
| 110 | inception_4e-output | Depth concatenation | 6×6×832 | - |  | 0 |
|  | Depth concatenation of 4 inputs |  |  |  |  |  |
| 111 | pool4-3x3_s2 | Max Pooling | 3×3×832 | - |  | 0 |
|  | 3x3 max pooling with stride [2 2] and padding [0 1 0 1] |  |  |  |  |  |
| 112 | inception_5a-1x1 | Convolution | 3×3×256 | Weights | 1×1×832×256 | 213248 |
|  | 256 1x1x832 convolutions with stride [1 1] and padding [0 0 0 0] |  |  | Bias | 1×1×256 |  |
| 113 | inception_5a-relu_1x1 | ReLU | 3×3×256 | - |  | 0 |
|  | ReLU |  |  |  |  |  |
| 114 | inception_5a-3x3_reduce | Convolution | 3×3×160 | Weights | 1×1×832×16 | 133280 |
|  | 160 1x1x832 convolutions with stride [1 1] and padding [0 0 0 0] |  |  | Bias | 1×1×16 |  |
| 115 | inception_5a-relu_3x3_reduce | ReLU | 3×3×160 | - |  | 0 |
|  | ReLU |  |  |  |  |  |
| 116 | inception_5a-3x3 | Convolution | 3×3×320 | Weights | 3×3×16 ×32 | 461120 |
|  | 320 3x3x160 convolutions with stride [1 1] and padding [1 1 1 1] |  |  | Bias | 1×1×32 |  |
| 117 | inception_5a-relu_3x3 | ReLU | 3×3×320 | - |  | 0 |
|  | ReLU |  |  |  |  |  |
| 118 | inception_5a-5x5_reduce | Convolution | 3×3×32 | Weights | 1×1×832×32 | 26656 |
|  | 32 1x1x832 convolutions with stride [1 1] and padding [0 0 0 0] |  |  | Bias | 1×1×32 |  |
| 119 | inception_5a-relu_5x5_reduce | ReLU | 3×3×32 | - |  | 0 |
|  | ReLU |  |  |  |  |  |
| 120 | inception_5a-5x5 | Convolution | 3×3×128 | Weights | 5×5×32×128 | 102528 |
|  | 128 5x5x32 convolutions with stride [1 1] and padding [2 2 2 2] |  |  | Bias | 1×1×128 |  |
| 121 | inception_5a-relu_5x5 | ReLU | 3×3×128 | - |  | 0 |
|  | ReLU |  |  |  |  |  |
| 122 | inception_5a-pool | Max Pooling | 3×3×832 | - |  | 0 |
|  | 3x3 max pooling with stride [1 1] and padding [1 1 1 1] |  |  |  |  |  |
| 123 | inception_5a-pool_proj | Convolution | 3×3×128 | Weights | 1×1×832×128 | 106624 |
|  | 128 1x1x832 convolutions with stride [1 1] and padding [0 0 0 0] |  |  | Bias | 1×1×128 |  |
| 124 | inception_5a-relu_pool_proj | ReLU | 3×3×128 | - |  | 0 |
|  | ReLU |  |  |  |  |  |
| 125 | inception_5a-output | Depth concatenation | 3×3×832 | - |  | 0 |
|  | Depth concatenation of 4 inputs |  |  |  |  |  |
| 126 | inception_5b-1x1 | Convolution | 3×3×384 | Weights | 1×1×832×384 | 319872 |
|  | 384 1x1x832 convolutions with stride [1 1] and padding [0 0 0 0] |  |  | Bias | 1×1×384 |  |
| 127 | inception_5b-relu_1x1 | ReLU | 3×3×384 | - |  | 0 |
|  | ReLU |  |  |  |  |  |
| 128 | inception_5b-3x3_reduce | Convolution | 3×3×192 | Weights | 1×1×832×192 | 159936 |
|  | 192 1x1x832 convolutions with stride [1 1] and padding [0 0 0 0] |  |  | Bias | 1×1×192 |  |
| 129 | inception_5b-relu_3x3_reduce | ReLU | 3×3×192 | - |  | 0 |
|  | ReLU |  |  |  |  |  |
| 130 | inception_5b-3x3 | Convolution | 3×3×384 | Weights | 3×3×192×384 | 663936 |
|  | 384 3x3x192 convolutions with stride [1 1] and padding [1 1 1 1] |  |  | Bias | 1×1×384 |  |
| 131 | inception_5b-relu_3x3 | ReLU | 3×3×384 | - |  | 0 |
|  | ReLU |  |  |  |  |  |
| 132 | inception_5b-5x5_reduce | Convolution | 3×3×48 | Weights | 1×1×832×48 | 39984 |
|  | 48 1x1x832 convolutions with stride [1 1] and padding [0 0 0 0] |  |  | Bias | 1×1×48 |  |
| 133 | inception_5b-relu_5x5_reduce | ReLU | 3×3×48 | - |  | 0 |
|  | ReLU |  |  |  |  |  |
| 134 | inception_5b-5x5 | Convolution | 3×3×128 | Weights | 5×5×48×128 | 153728 |
|  | 128 5x5x48 convolutions with stride [1 1] and padding [2 2 2 2] |  |  | Bias | 1×1×128 |  |
| 135 | inception_5b-relu_5x5 | ReLU | 3×3×128 | - |  | 0 |
|  | ReLU |  |  |  |  |  |
| 136 | inception_5b-pool | Max Pooling | 3×3×832 | - |  | 0 |
|  | 3x3 max pooling with stride [1 1] and padding [1 1 1 1] |  |  |  |  |  |
| 137 | inception_5b-pool_proj | Convolution | 3×3×128 | Weights | 1×1×832×128 | 106624 |
|  | 128 1x1x832 convolutions with stride [1 1] and padding [0 0 0 0] |  |  | Bias | 1×1×128 |  |
| 138 | inception_5b-relu_pool_proj | ReLU | 3×3×128 | - |  | 0 |
|  | ReLU |  |  |  |  |  |
| 139 | inception_5b-output | Depth concatenation | 3×3×1024 | - |  | 0 |
|  | Depth concatenation of 4 inputs |  |  |  |  |  |
| 140 | pool5-7x7_s1 | Average Pooling | 3×3×1024 | - |  | 0 |
|  | 1x1 average pooling with stride [1 1] and padding [0 0 0 0] |  |  |  |  |  |
| 141 | pool5-drop_7x7_s1 | Dropout | 3×3×1024 | - |  | 0 |
|  | 40% dropout |  |  |  |  |  |
| 142 | loss3-classifier | Fully Connected | 1×1×2 | Weights | 2×9216 | 18434 |
|  | 2 fully connected layer |  |  | Bias | 2×1 |  |
| 143 | prob | Softmax | 1×1×2 | - |  | 0 |
|  | softmax |  |  |  |  |  |
| 144 | output | Classification Output | - | - |  | 0 |
|  | crossentropyex with classes 'imdc' and 'mdc' |  |  |  |  |  |

## Supplementary Data 7. CellNet^Nuc^ convolutional features

Following images are showing convolutional features at given layer (in title) of CellNet^Nuc^. Please find **Supplementary Data 4** for corresponding layer details to the title of each convolution maps.


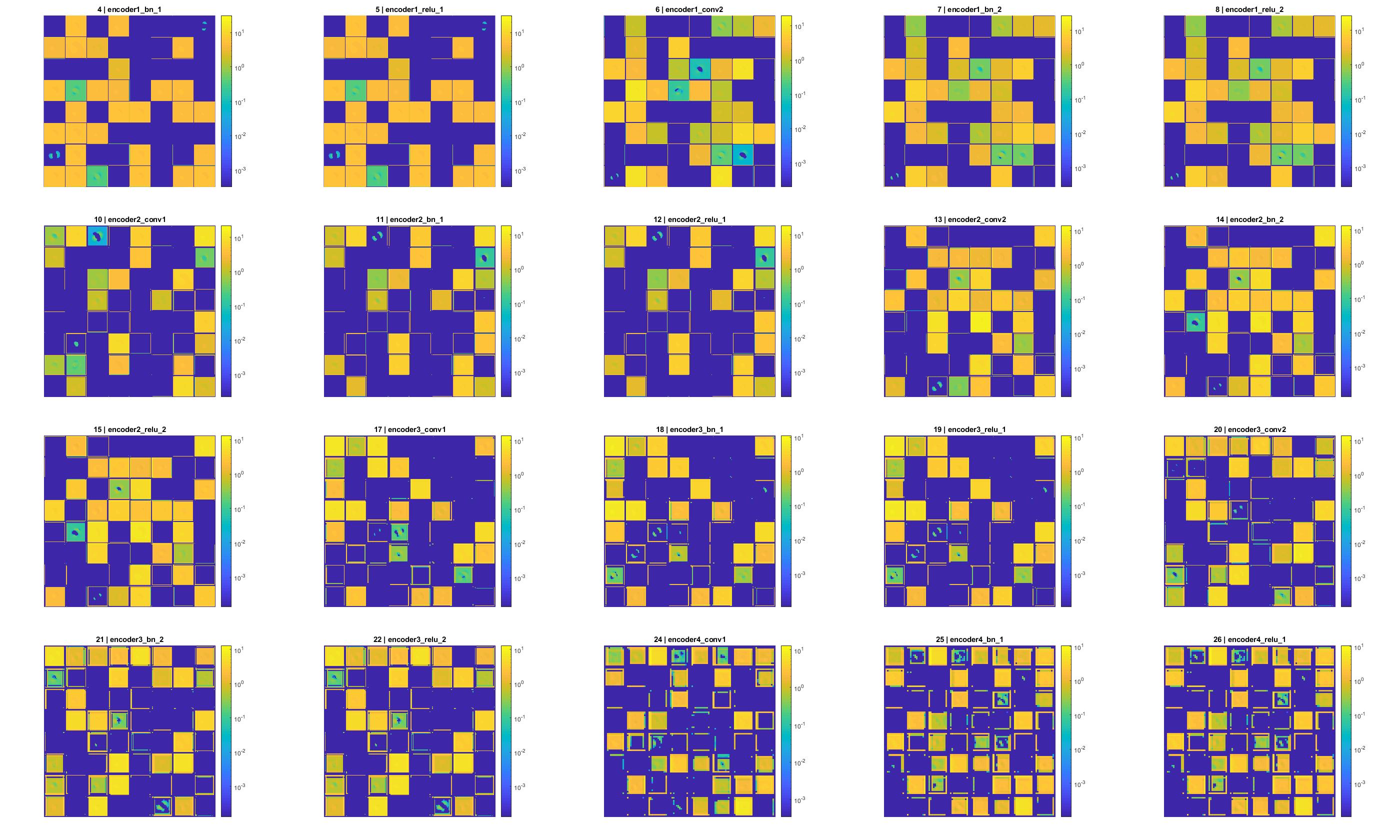


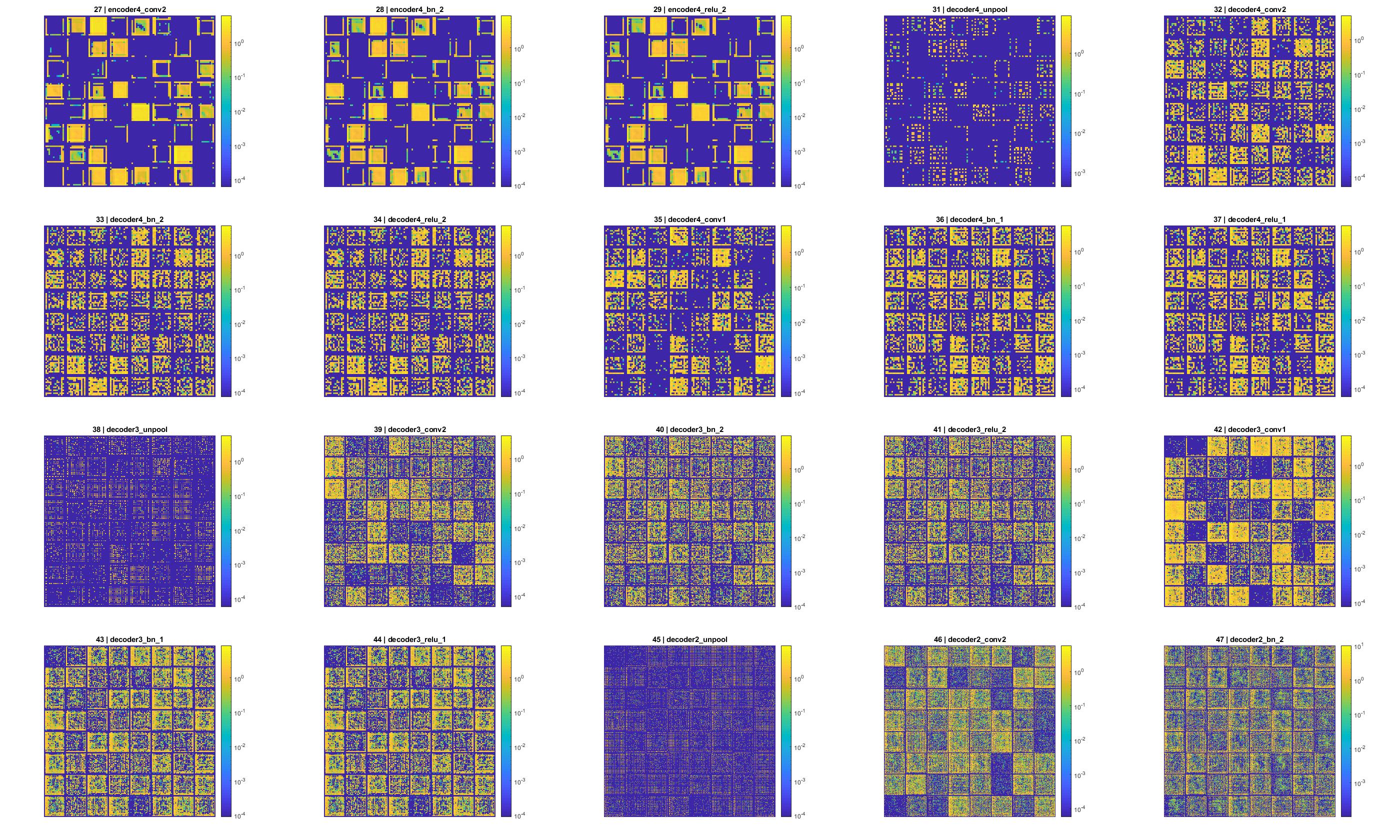


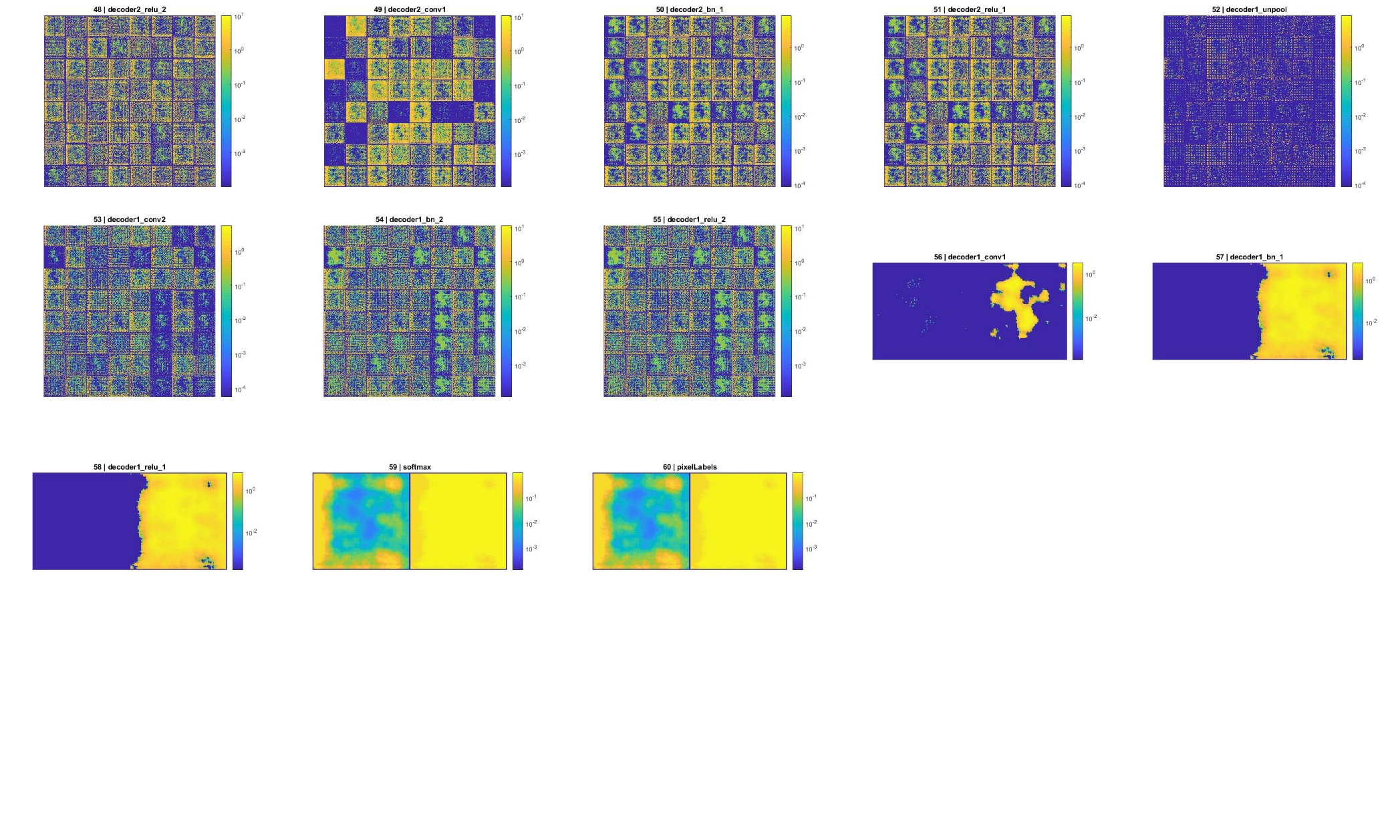


## Supplementary Data 8. CellNet^Mito^ convolutional features

Following images are showing convolutional features at given layer (in title) of CellNet^Mito^. Please find **Supplementary Data 5** for corresponding layer details to the title of each convolution maps.


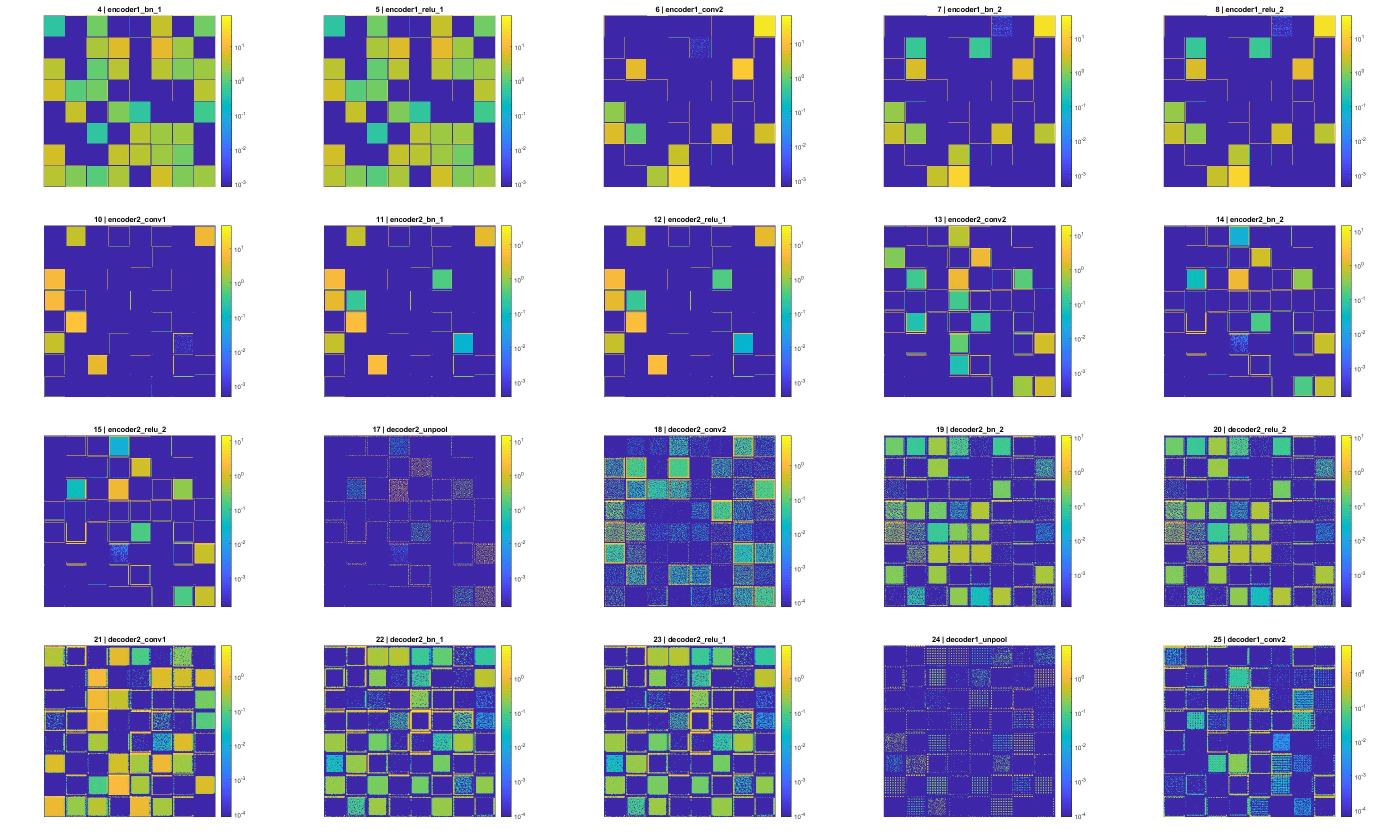


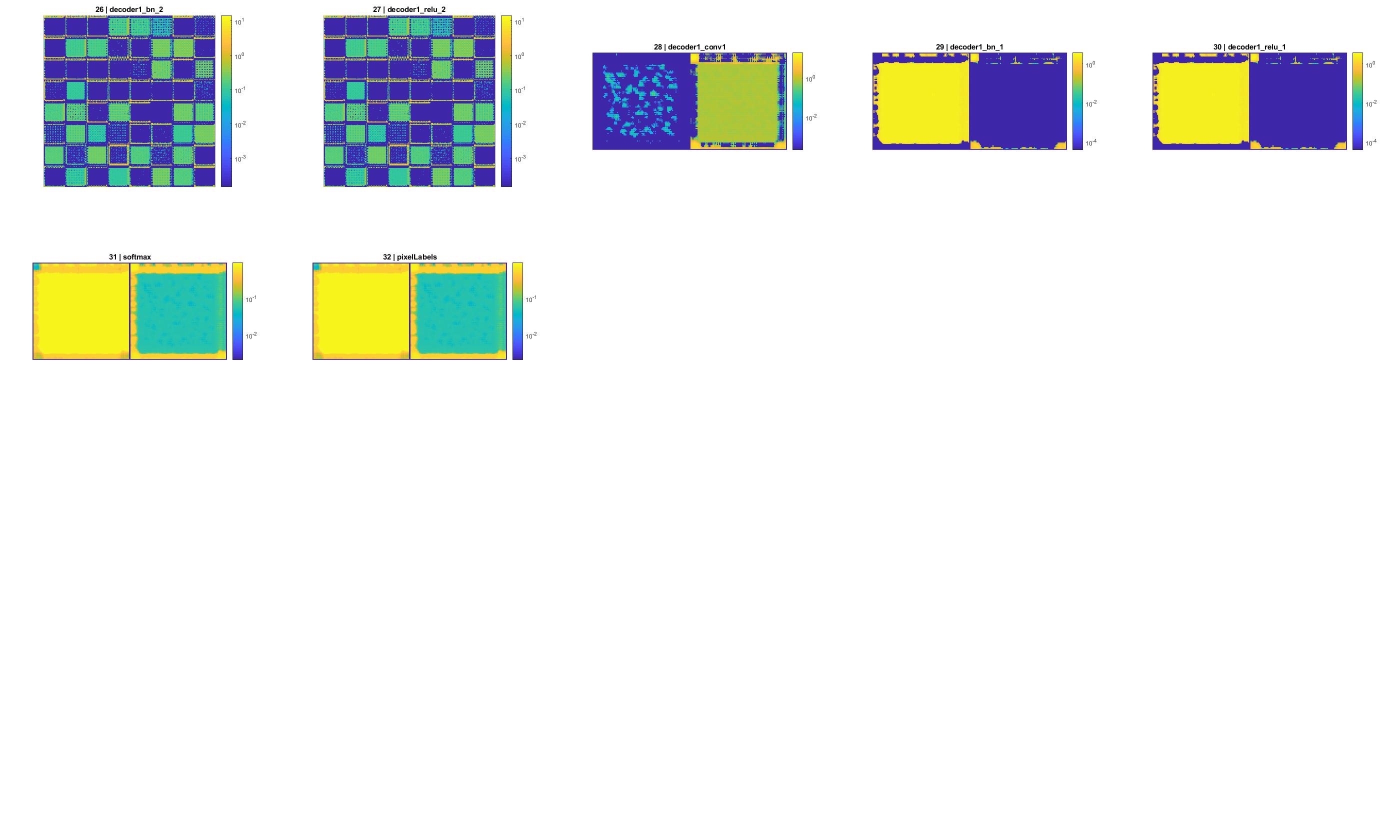


## Supplementary Data 9. CellNet^Actin^ convolutional features

Following images are showing convolutional features at given layer (in title) of CellNet^Actin^. Please find **Supplementary Data 6** for corresponding layer details to the title of each convolution maps.


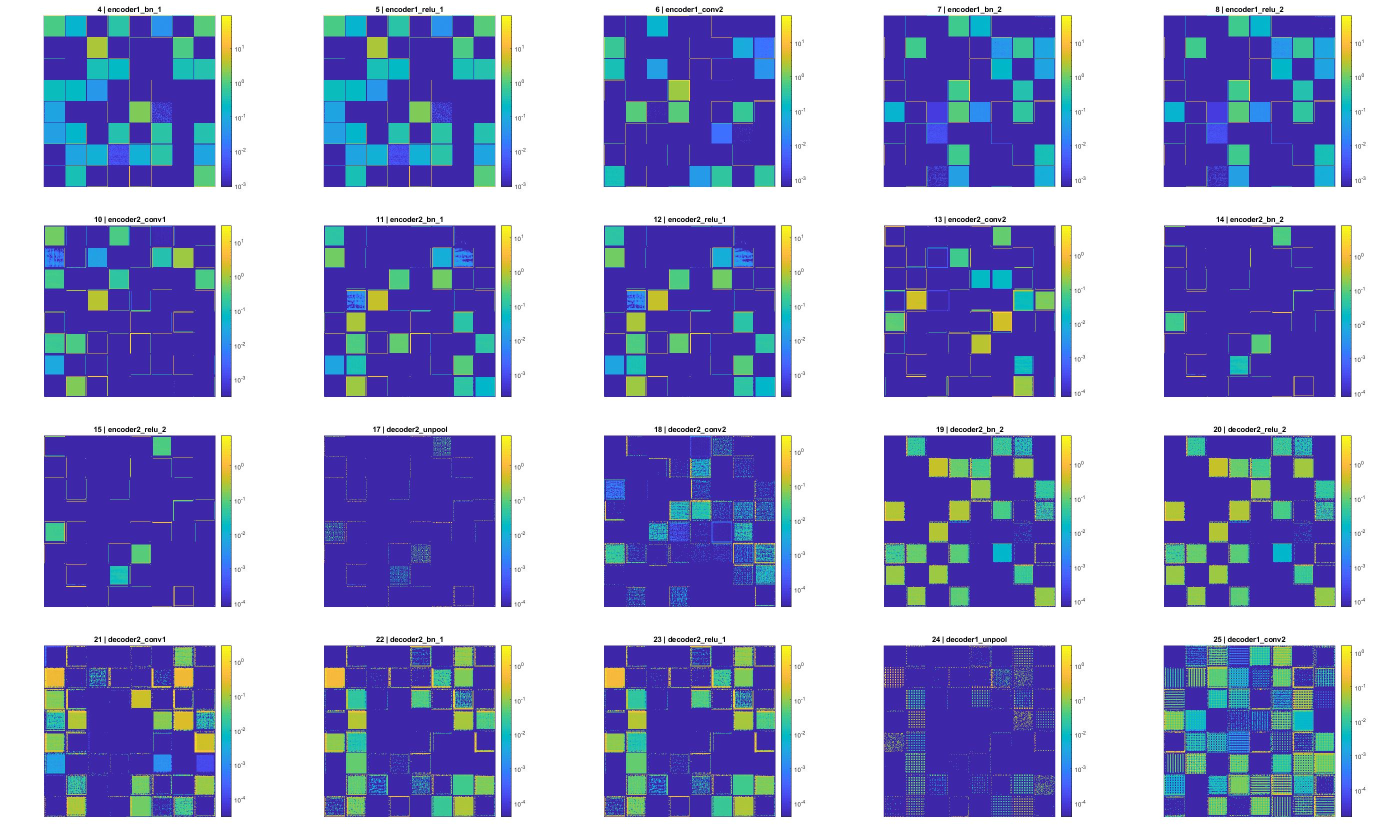


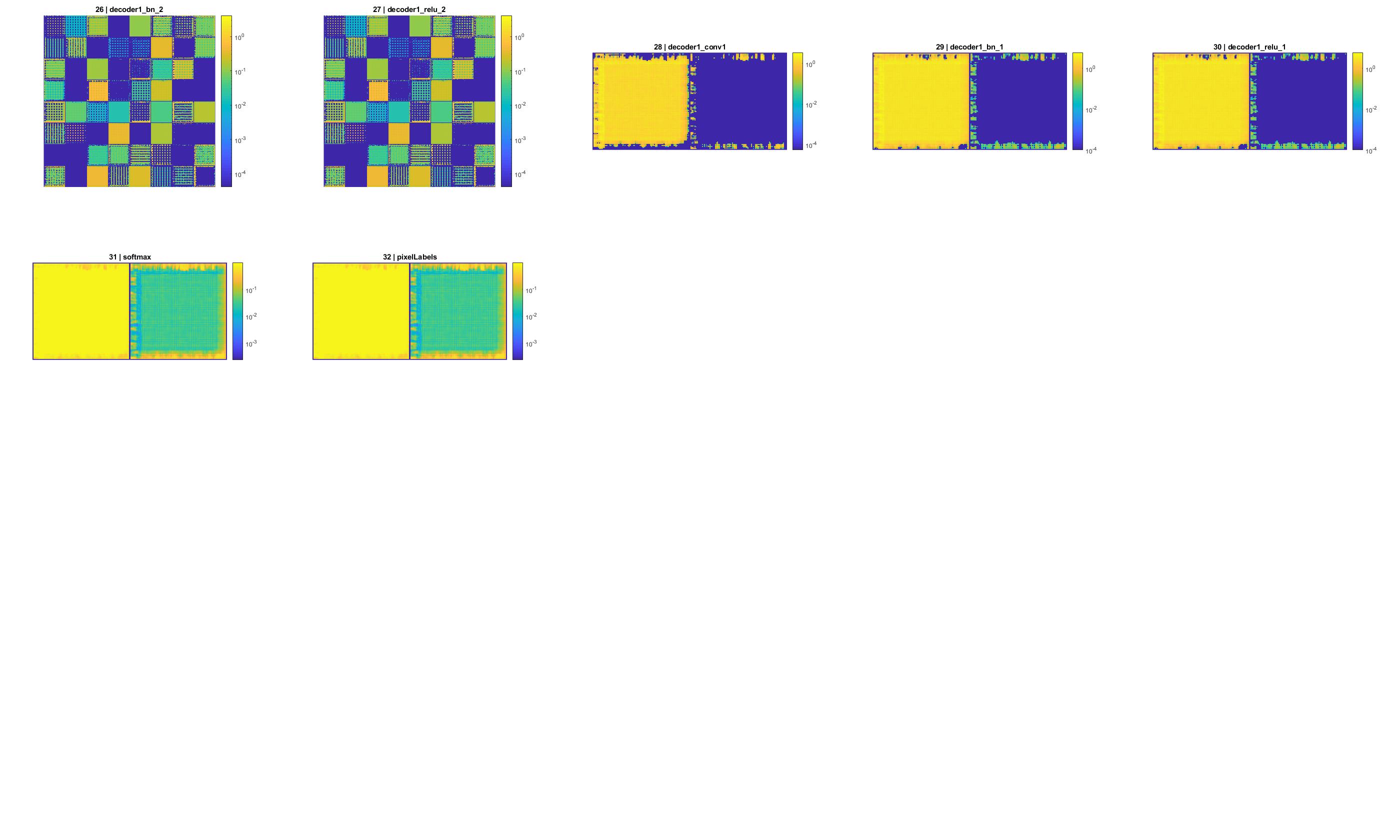


## Supplementary Data 10. ClassNet^Viability^ convolutional features

Following images are showing convolutional features at given layer (in title) of CellNet^Viability^. Please find **Supplementary Data 1** for corresponding layer details to the title of each convolution maps.


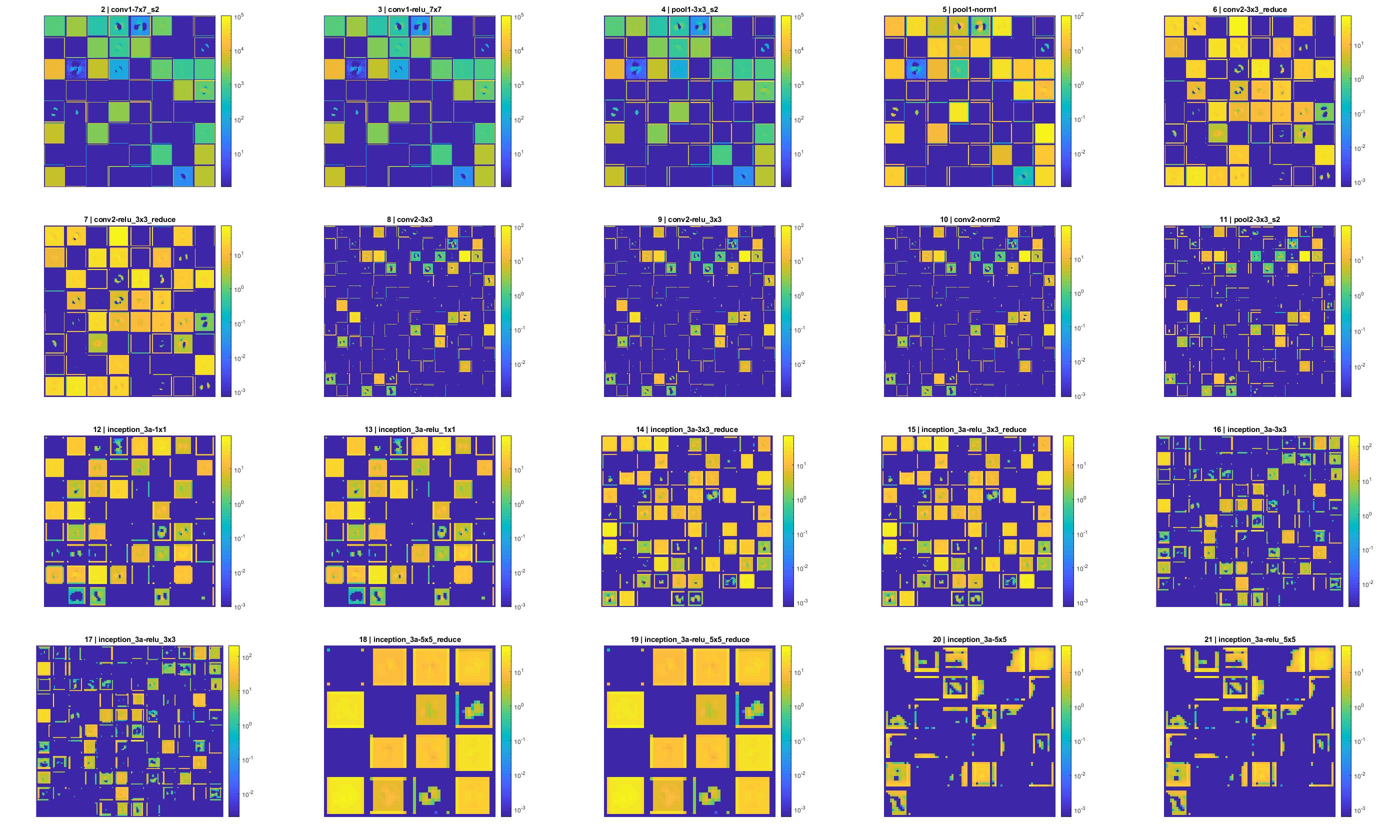


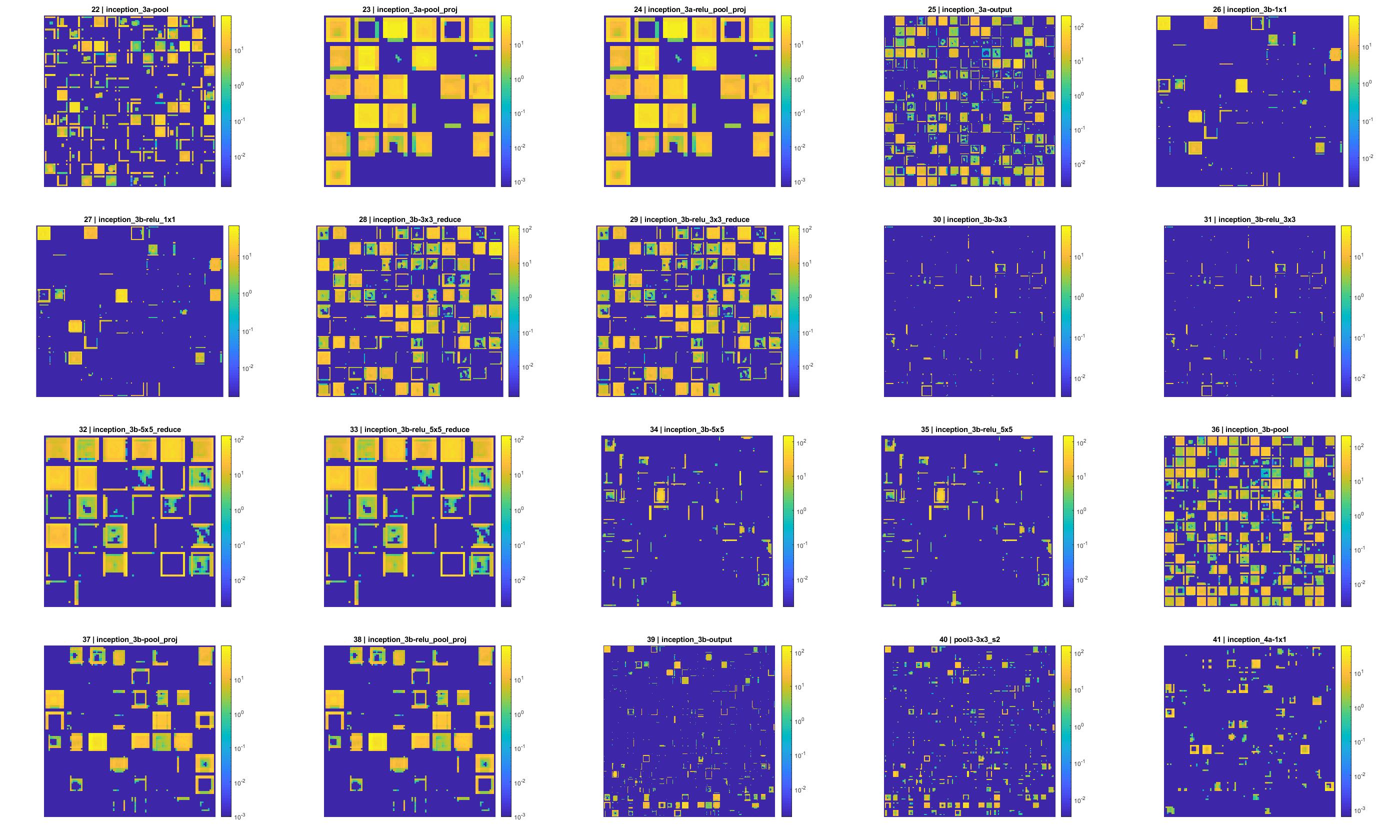

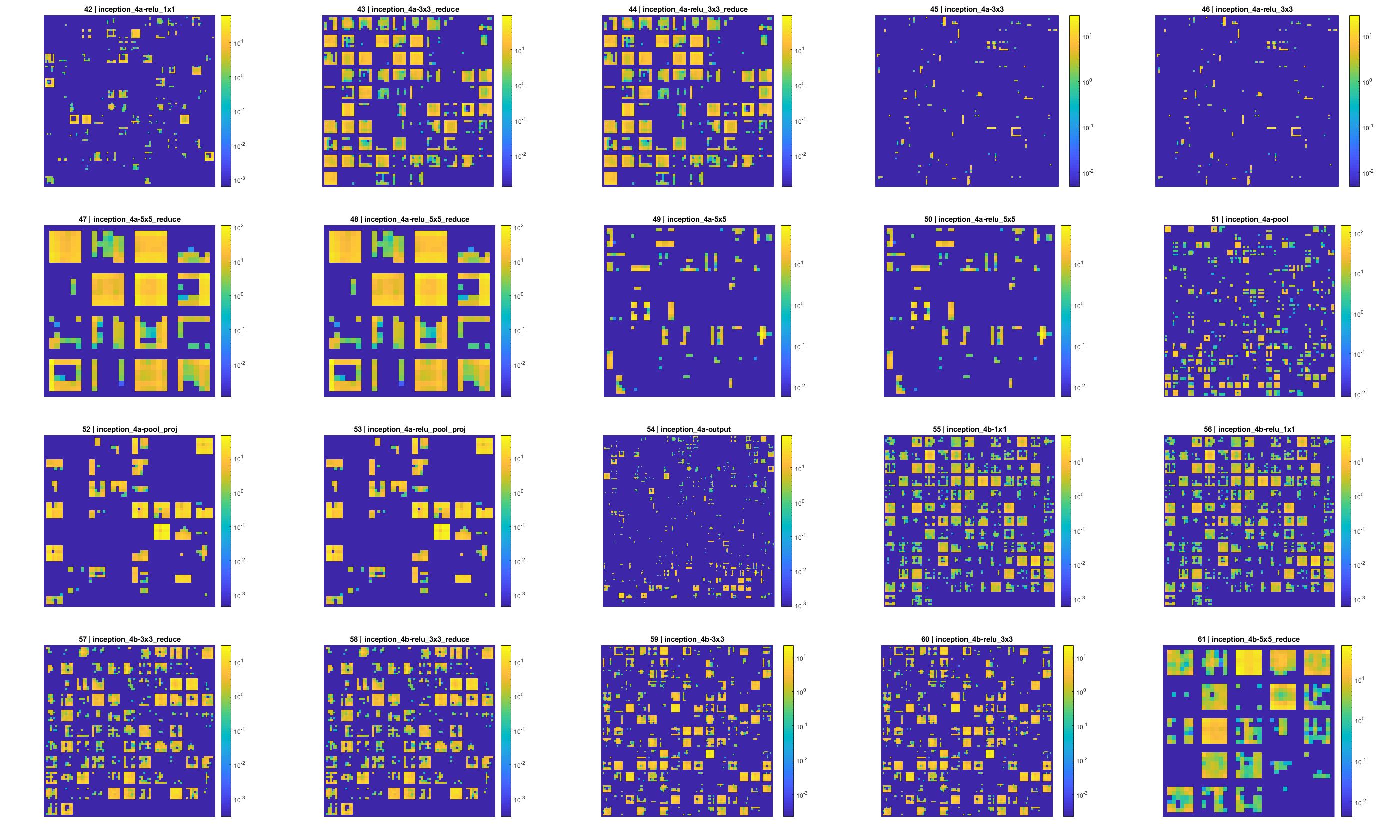


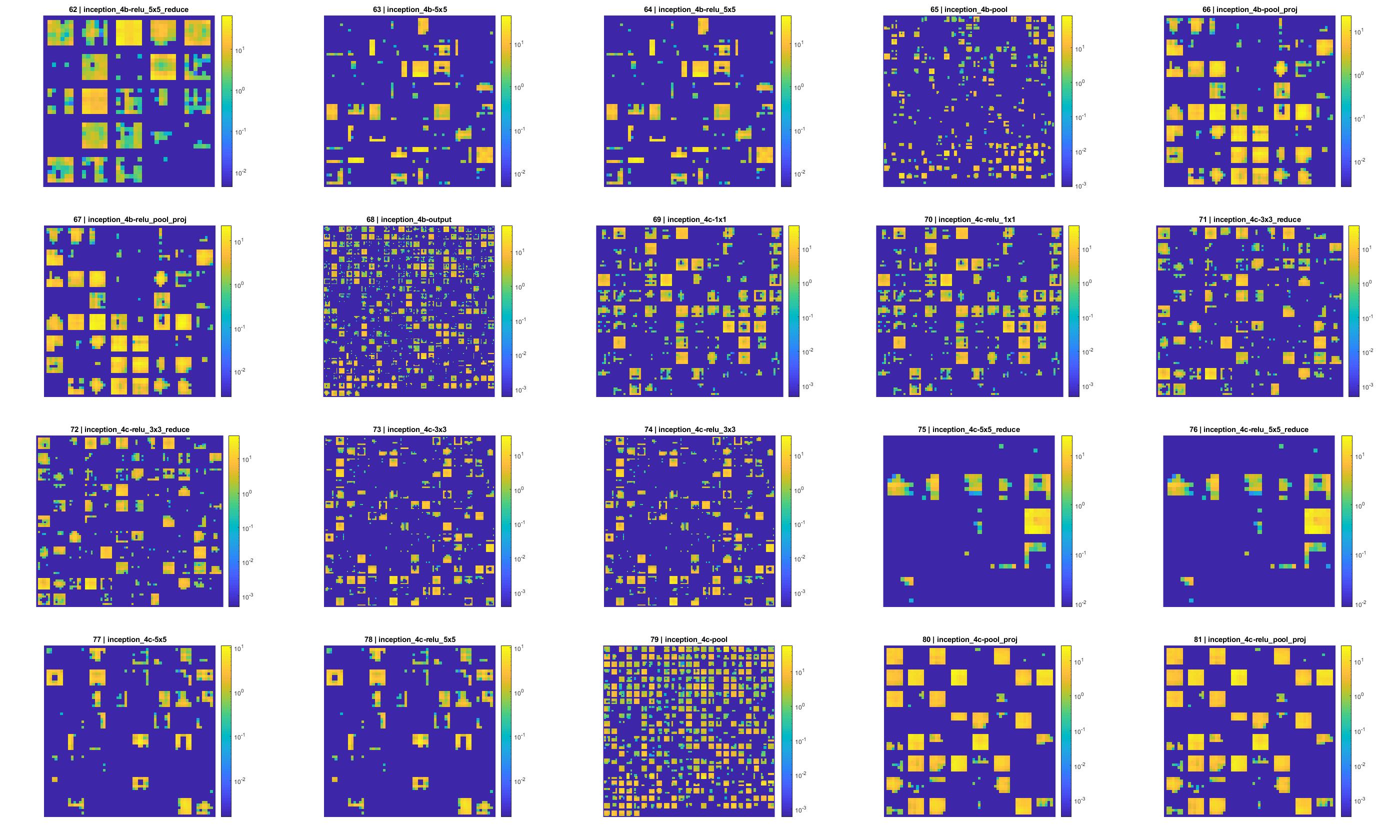


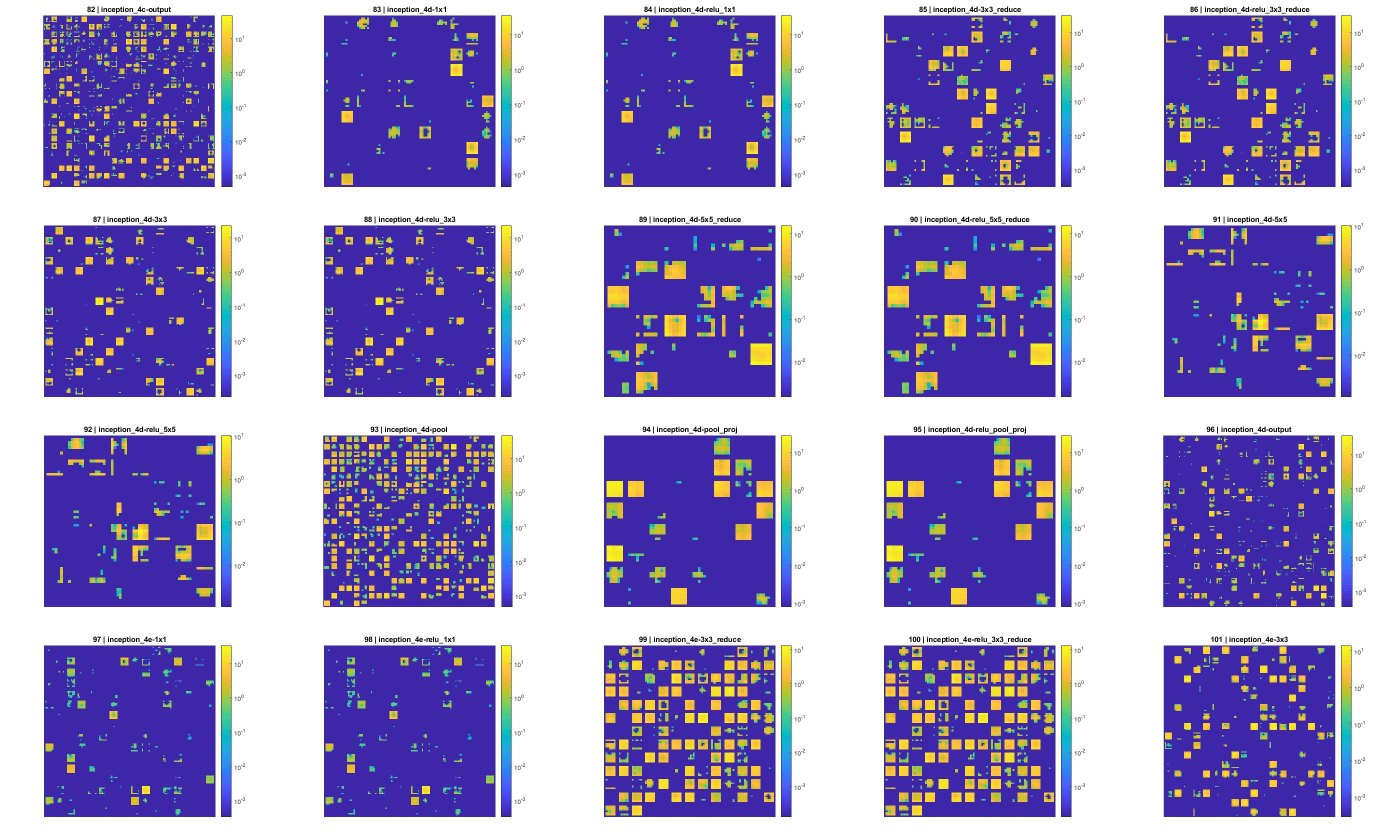


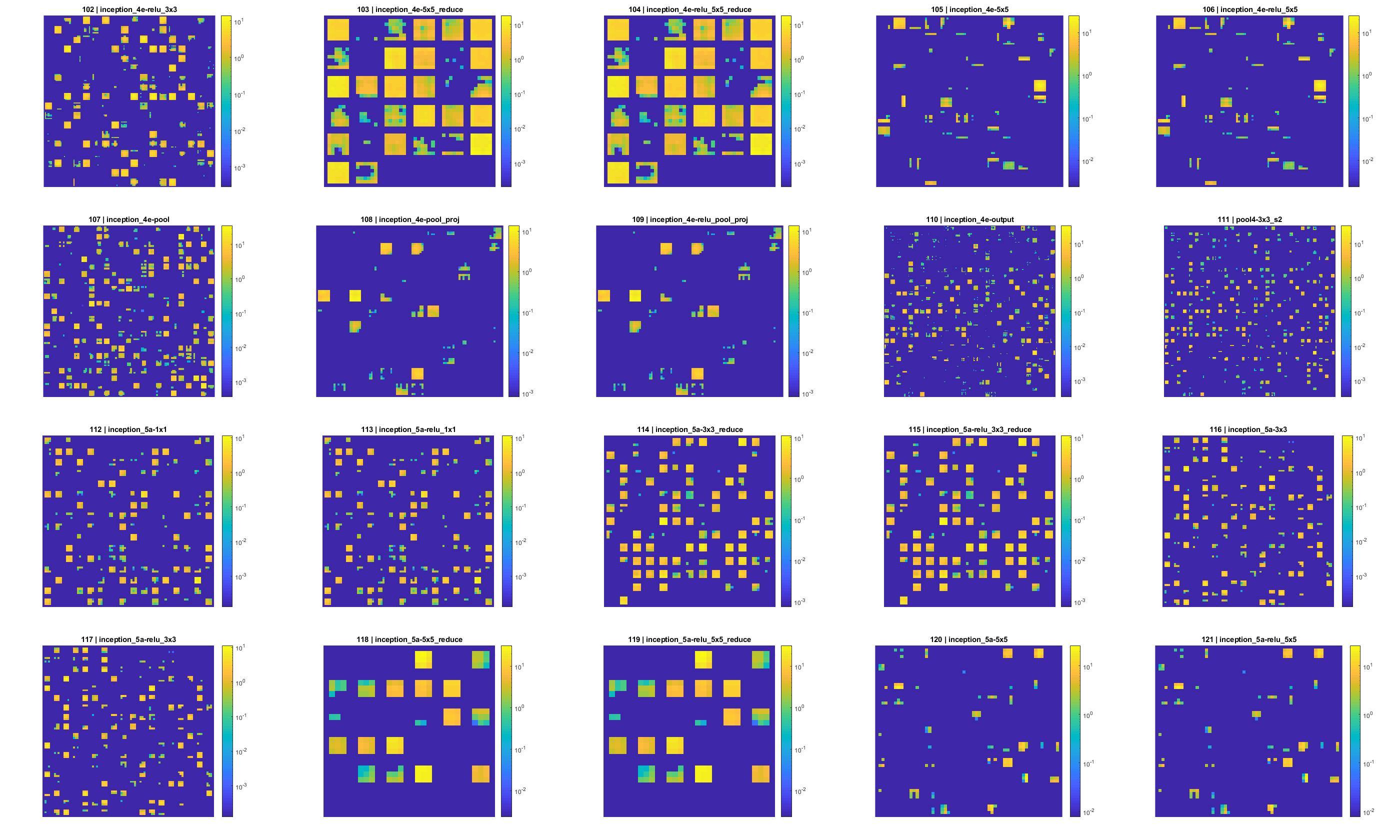


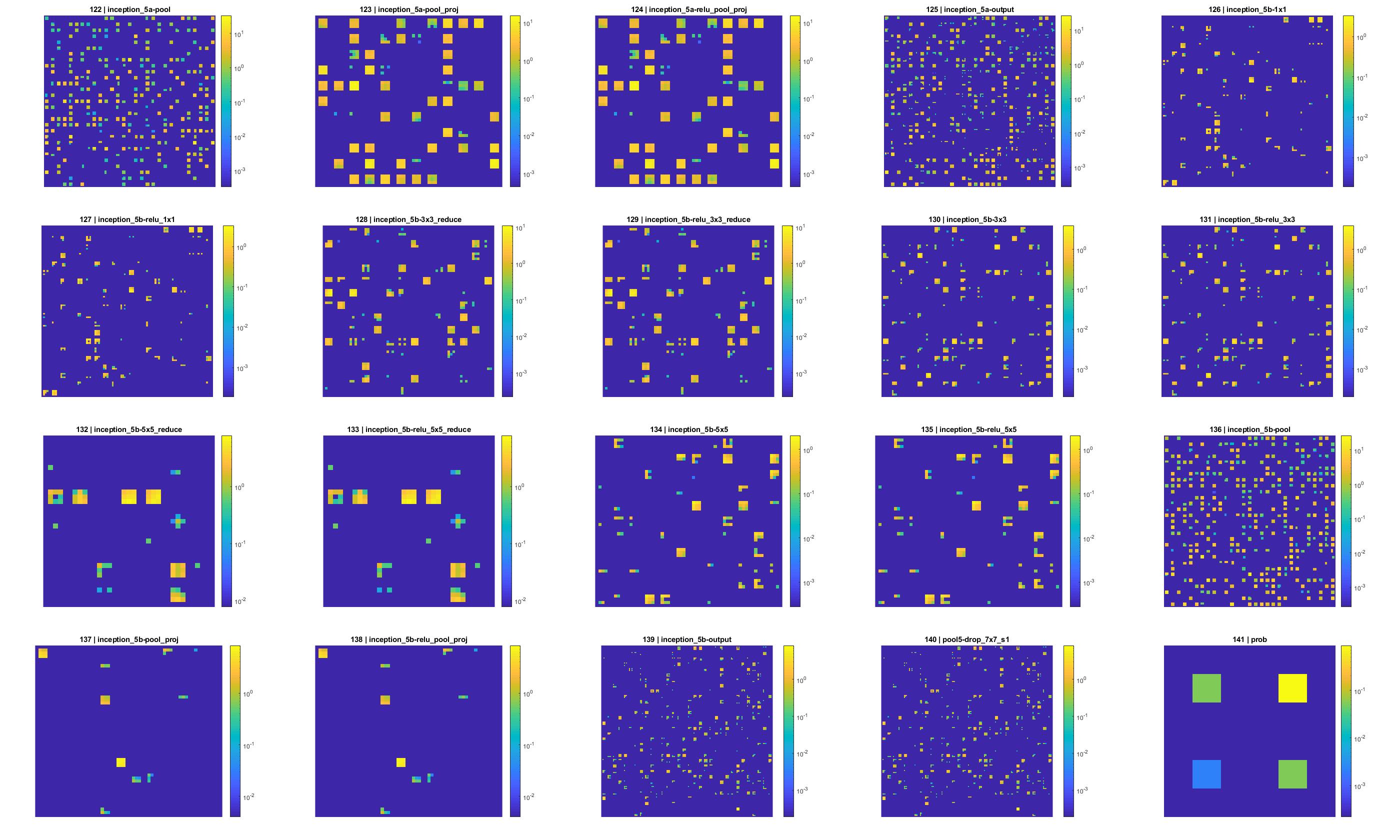


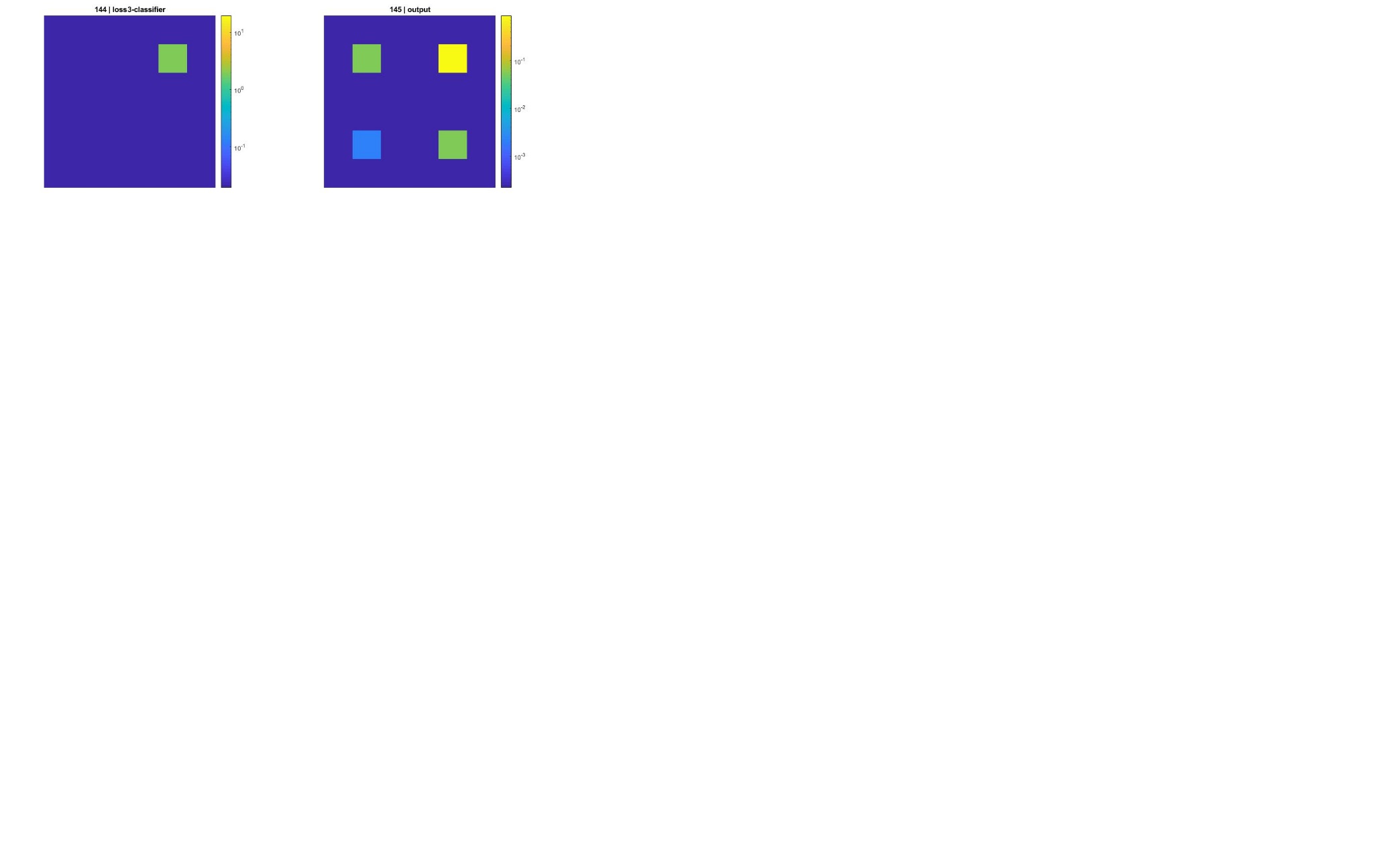


## Supplementary Data 11. ClassNet^Cancer^ convolutional features

Following images are showing convolutional features at given layer (in title) of CellNet^Cancer^. Please find **Supplementary Data 2** for corresponding layer details to the title of each convolution maps.


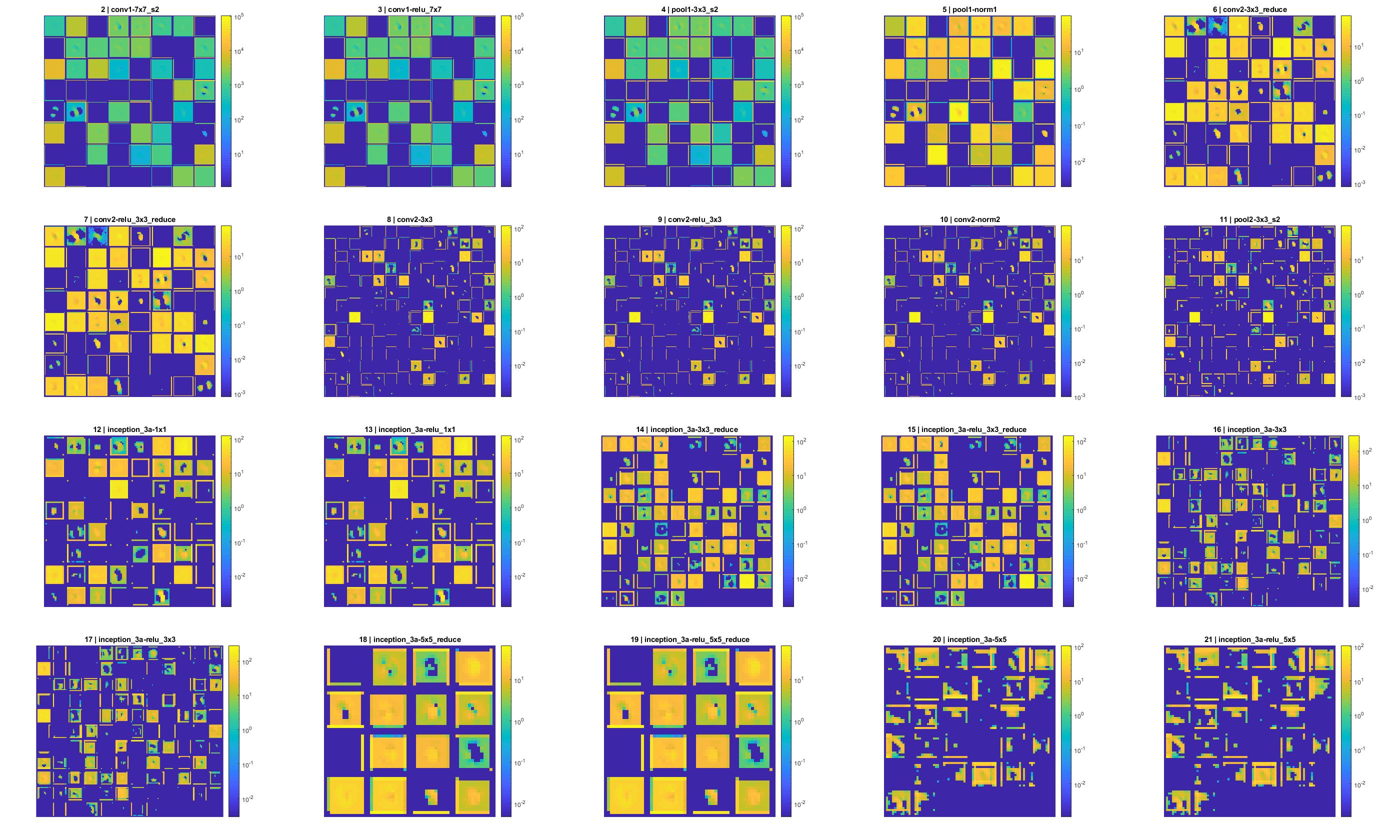


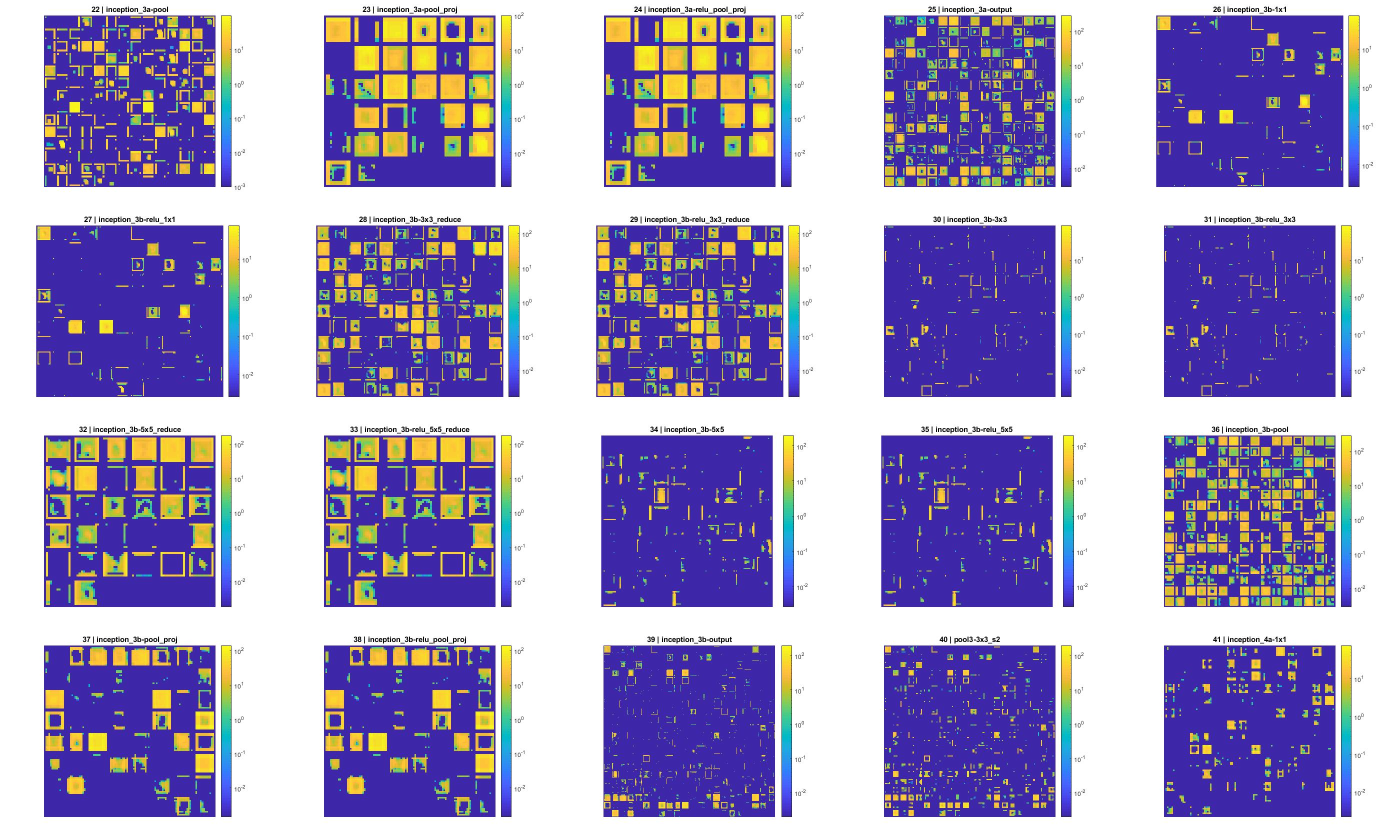


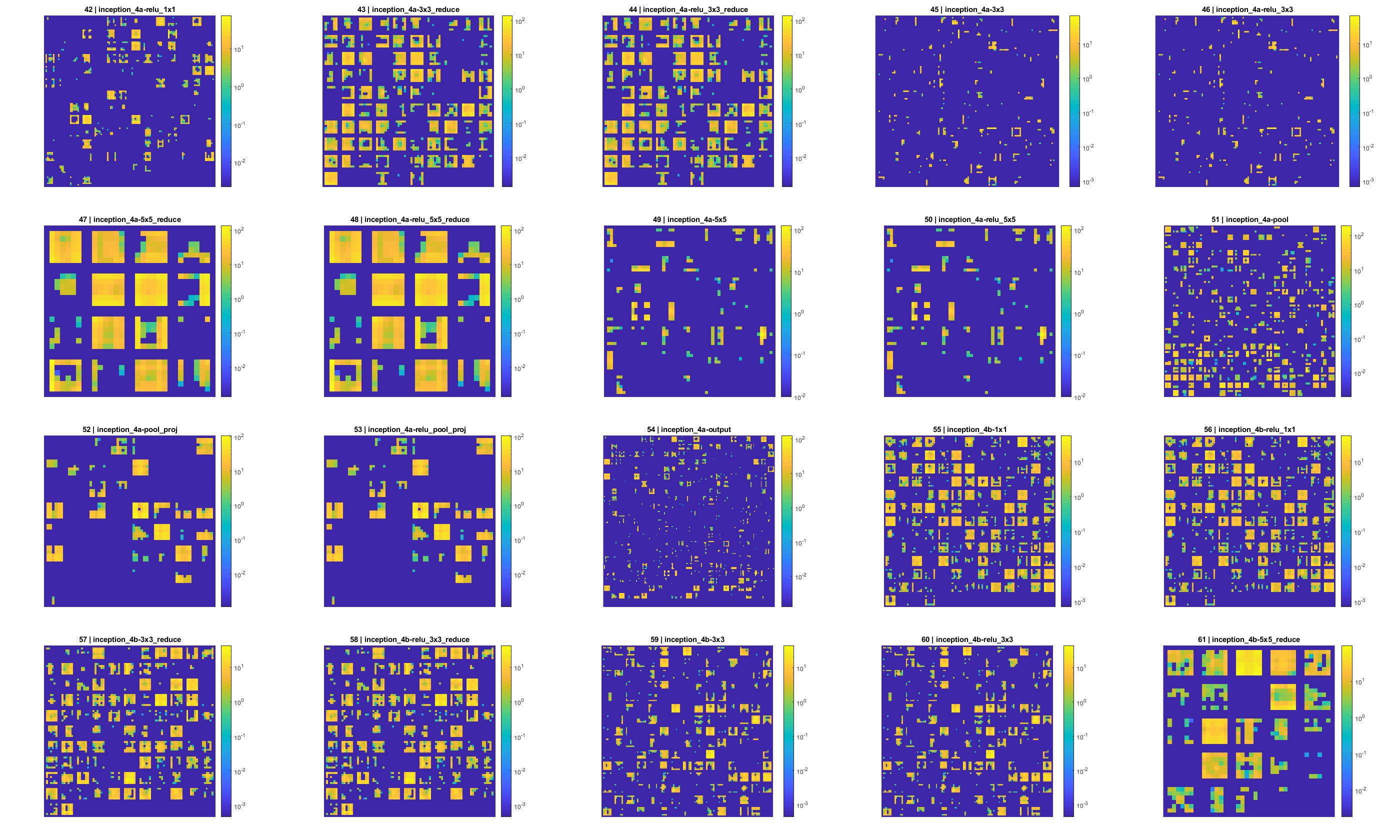


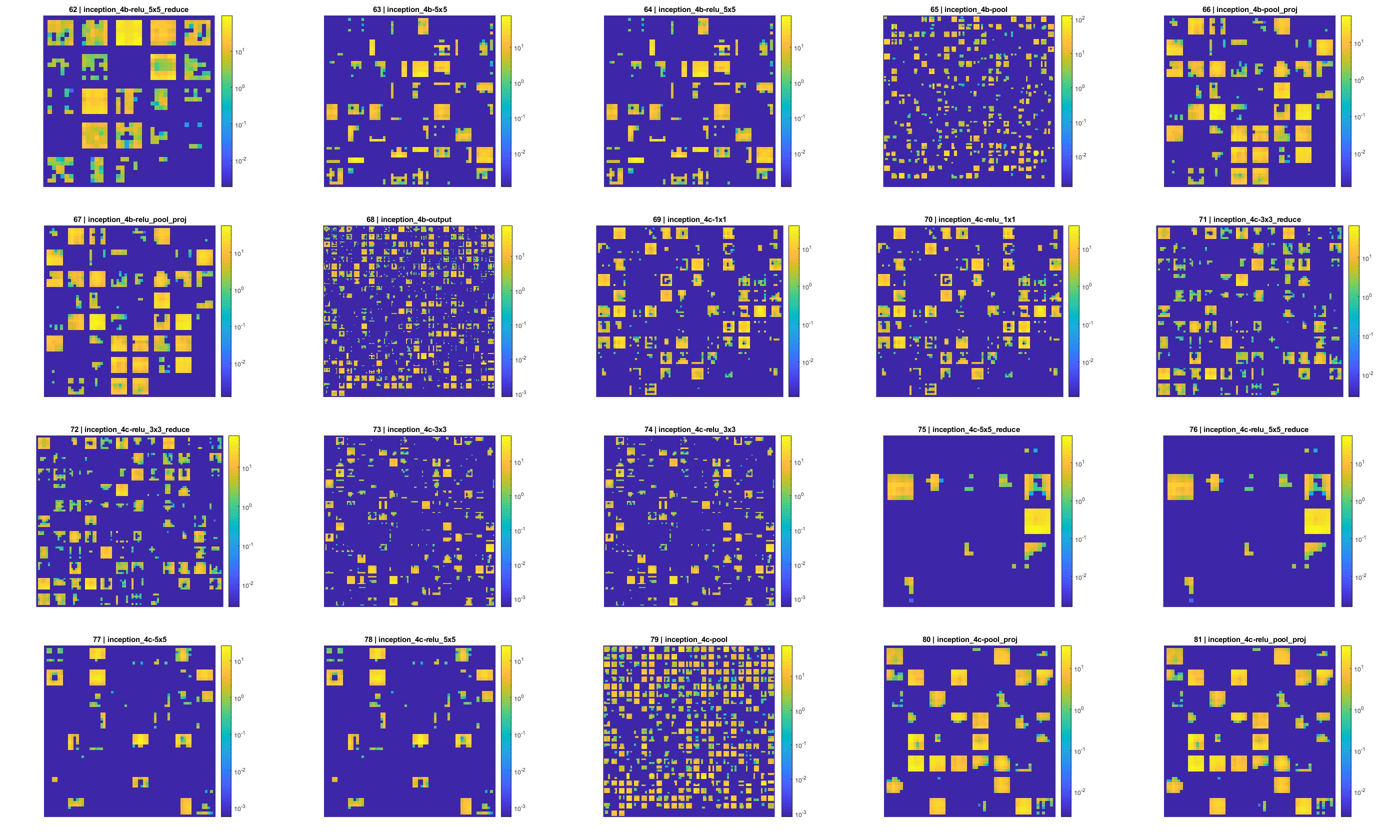


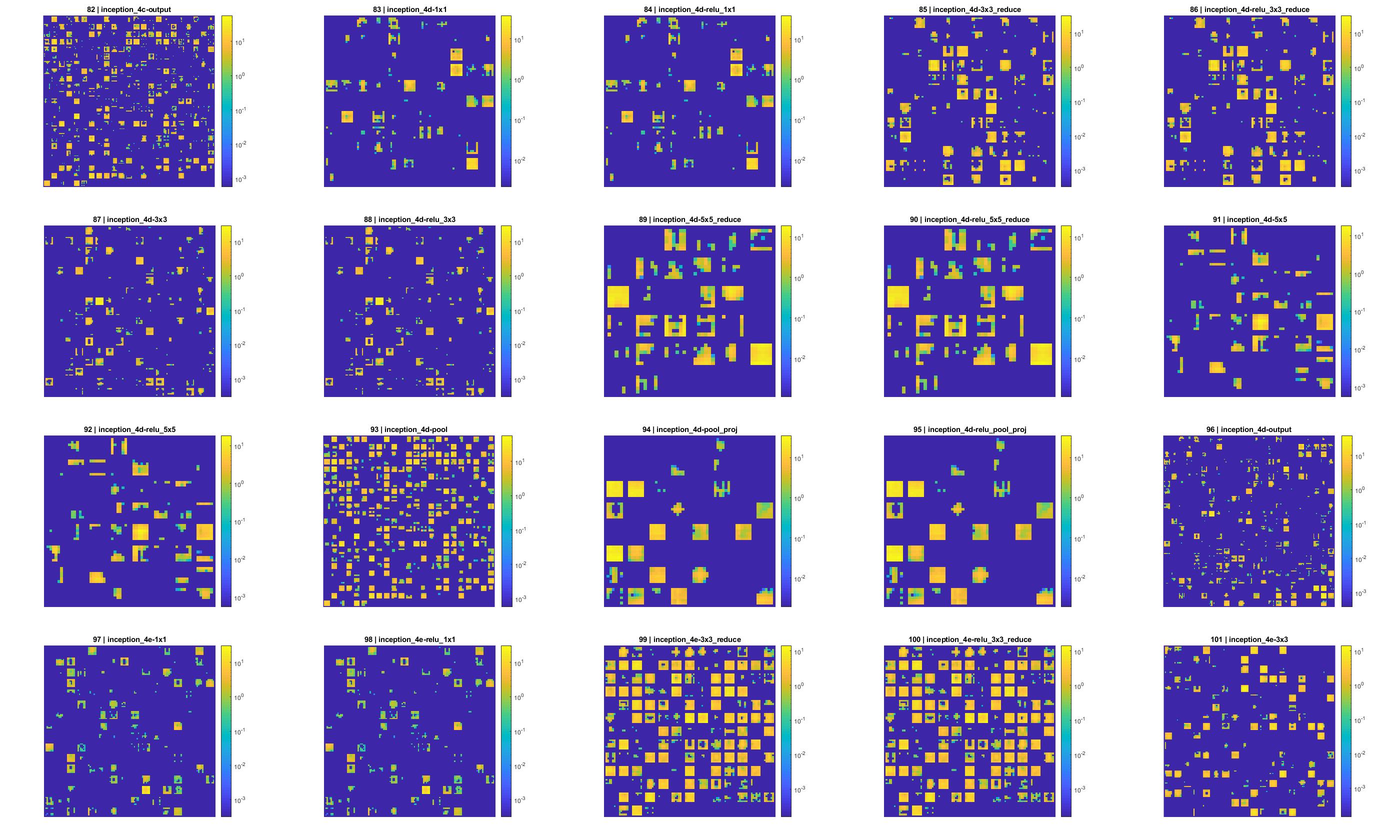


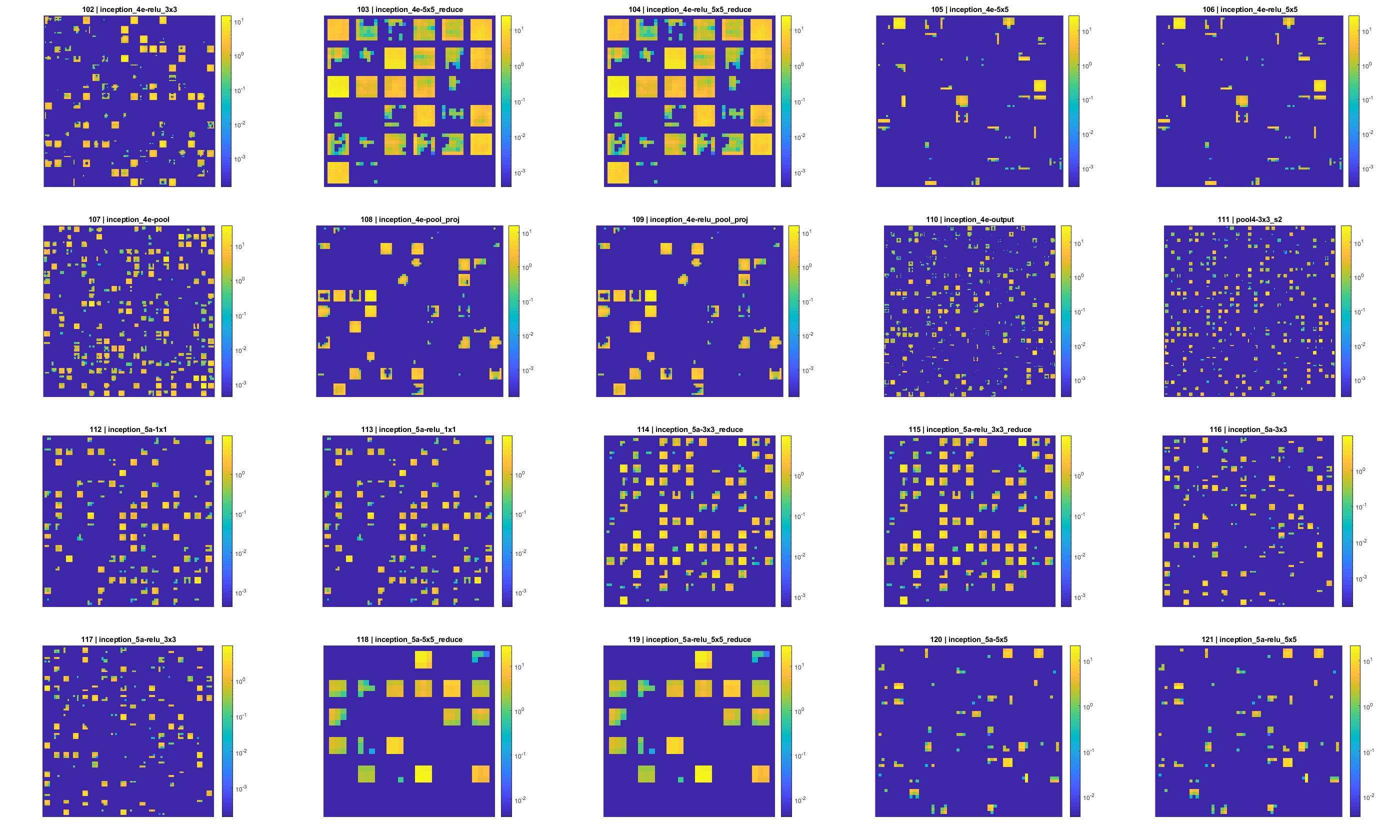


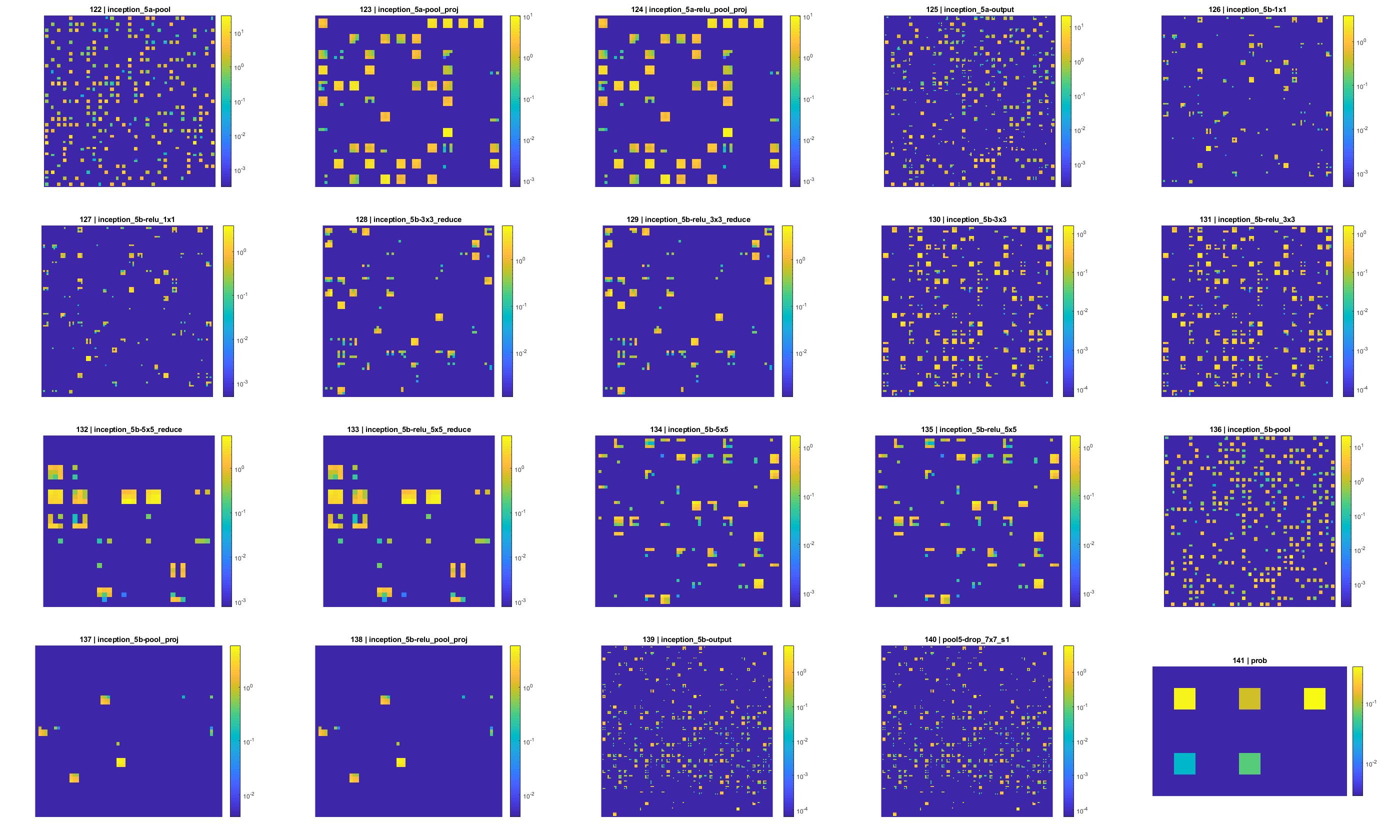


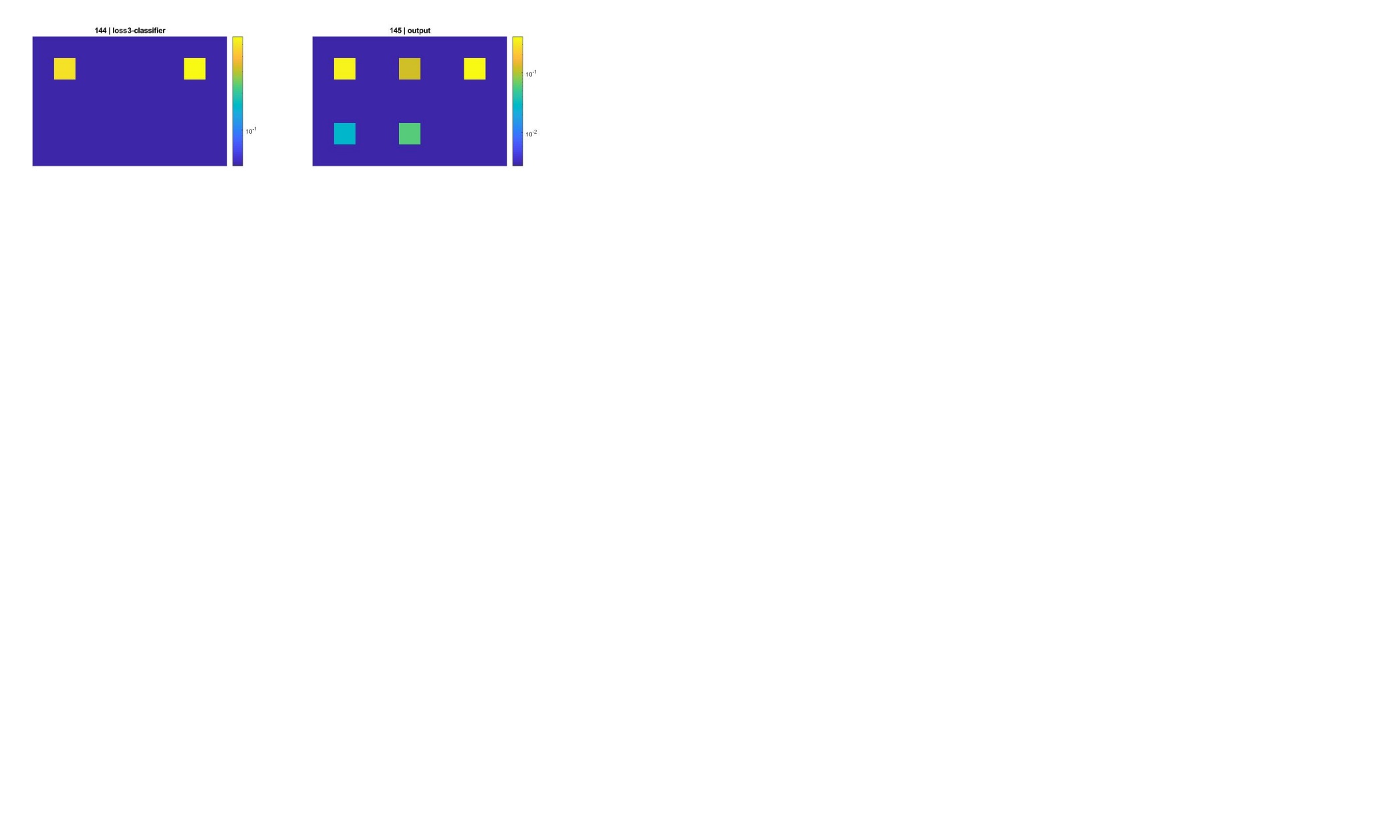


## Supplementary Data 12. ClassNet^DC^ convolutional features

Following images are showing convolutional features at given layer (in title) of CellNet^DC^. Please find **Supplementary Data 3** for corresponding layer details to the title of each convolution maps


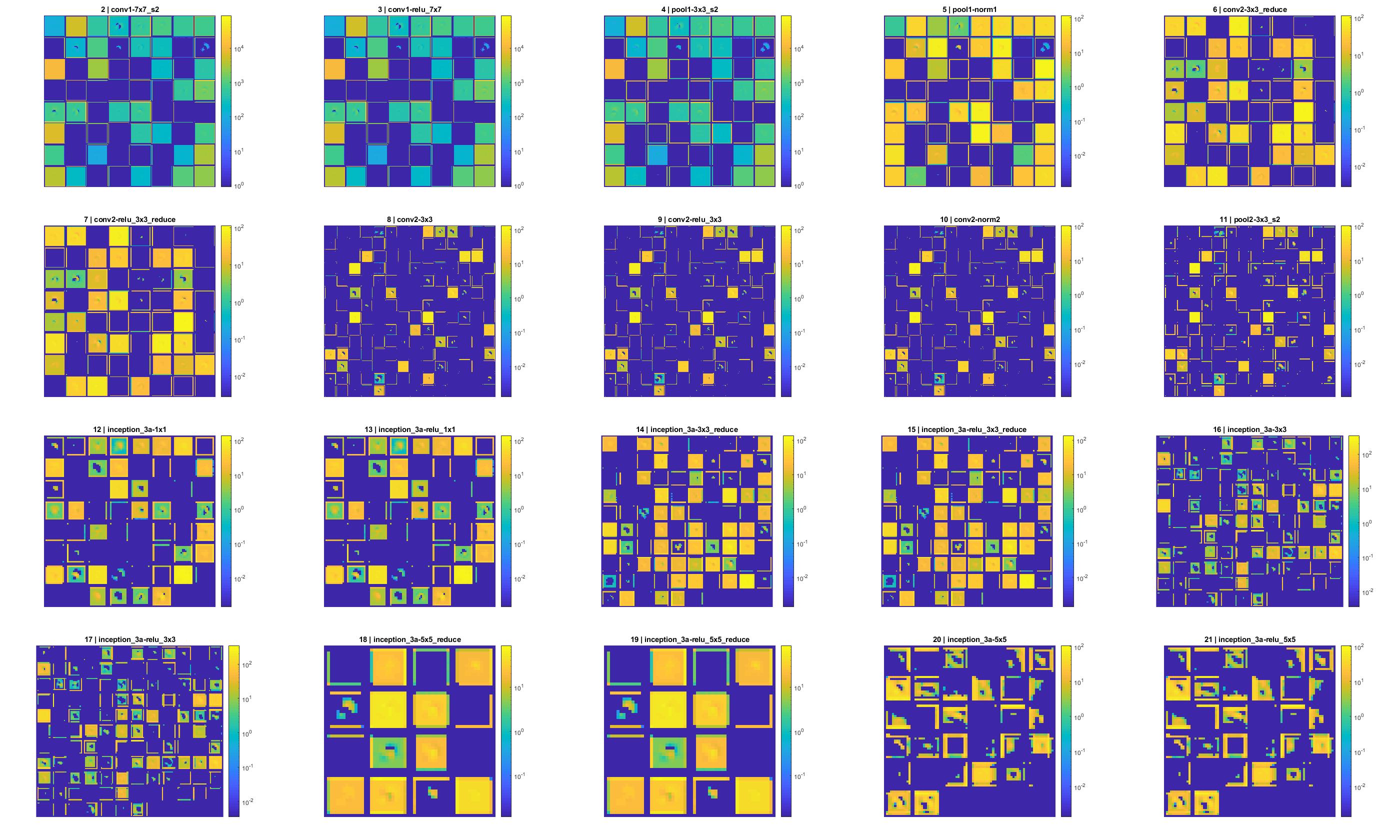


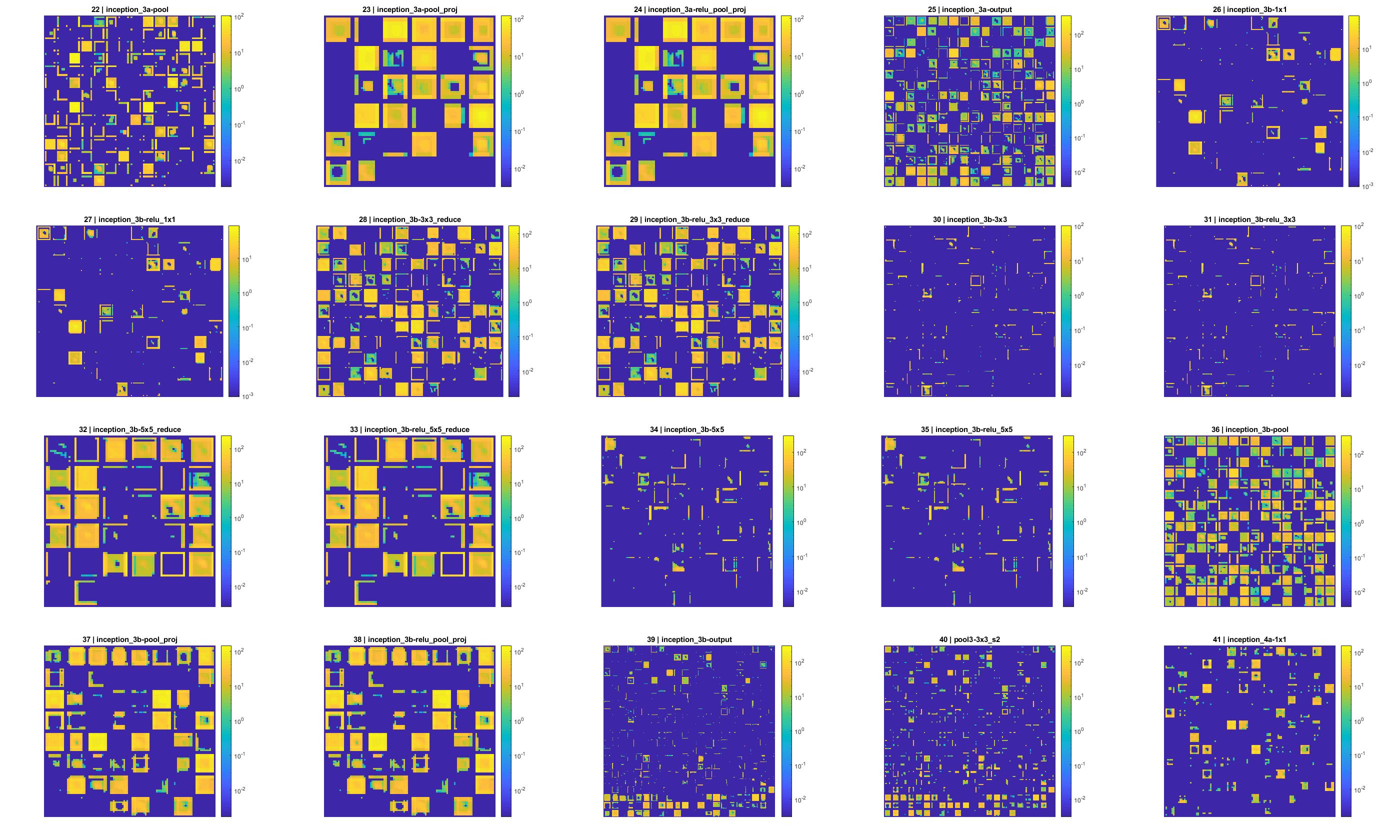


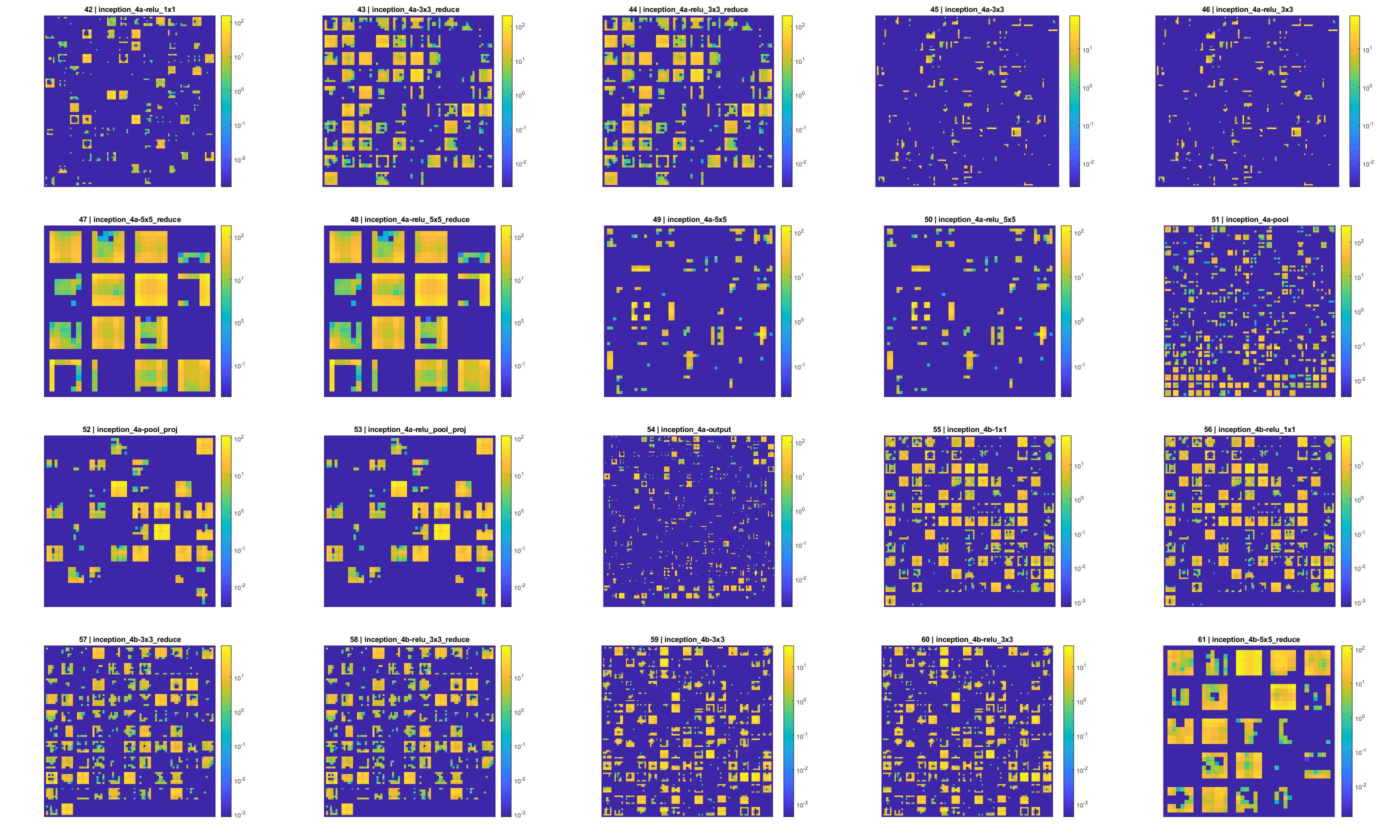


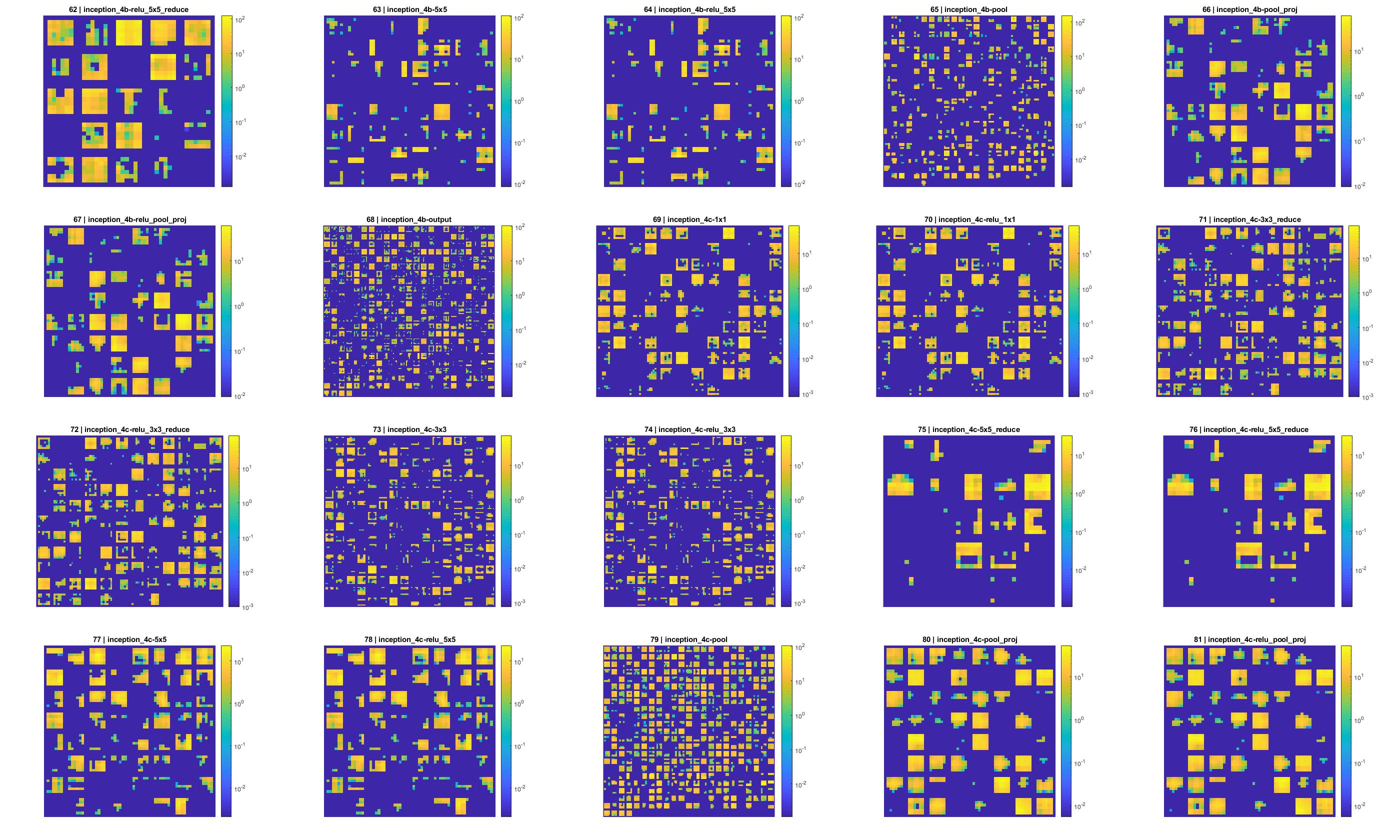


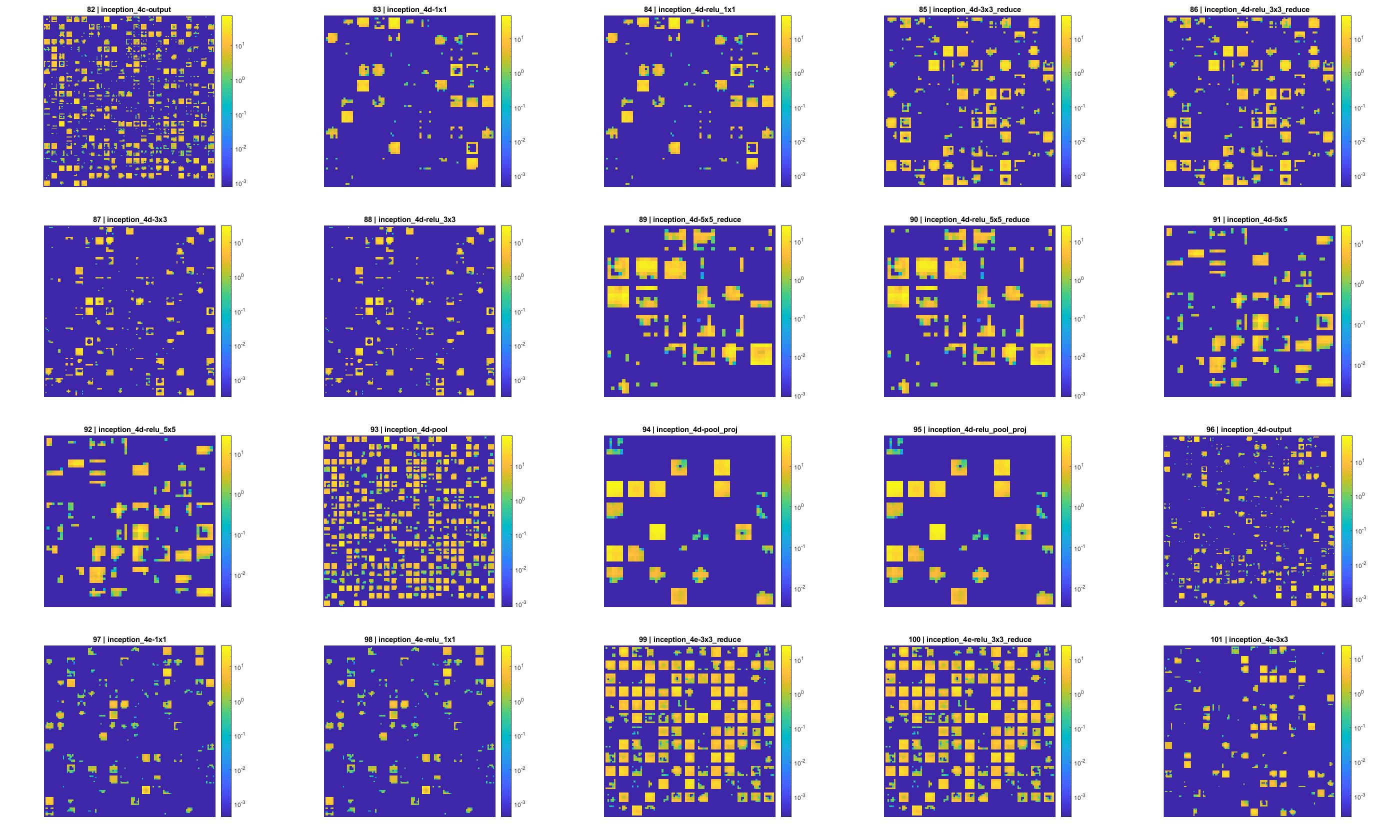


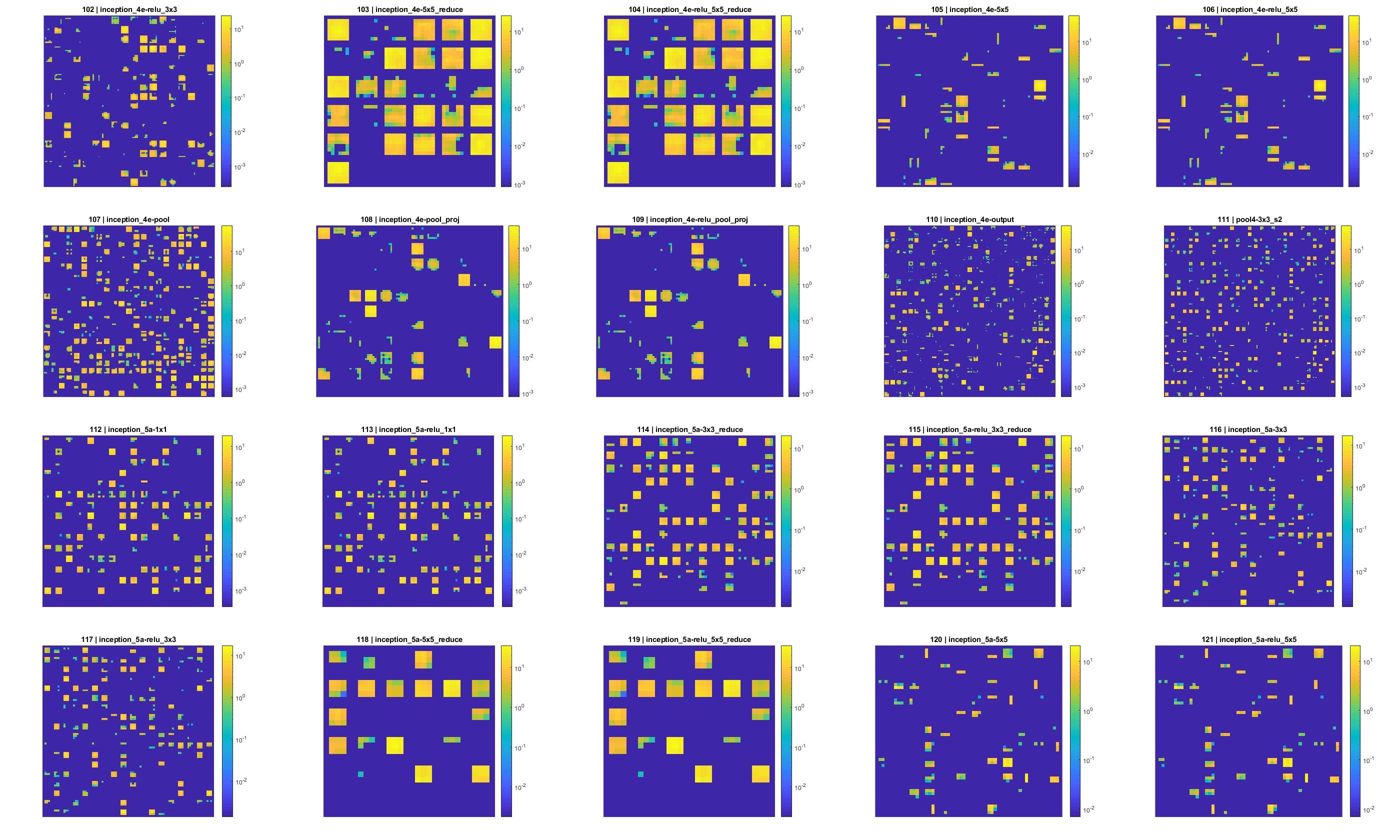


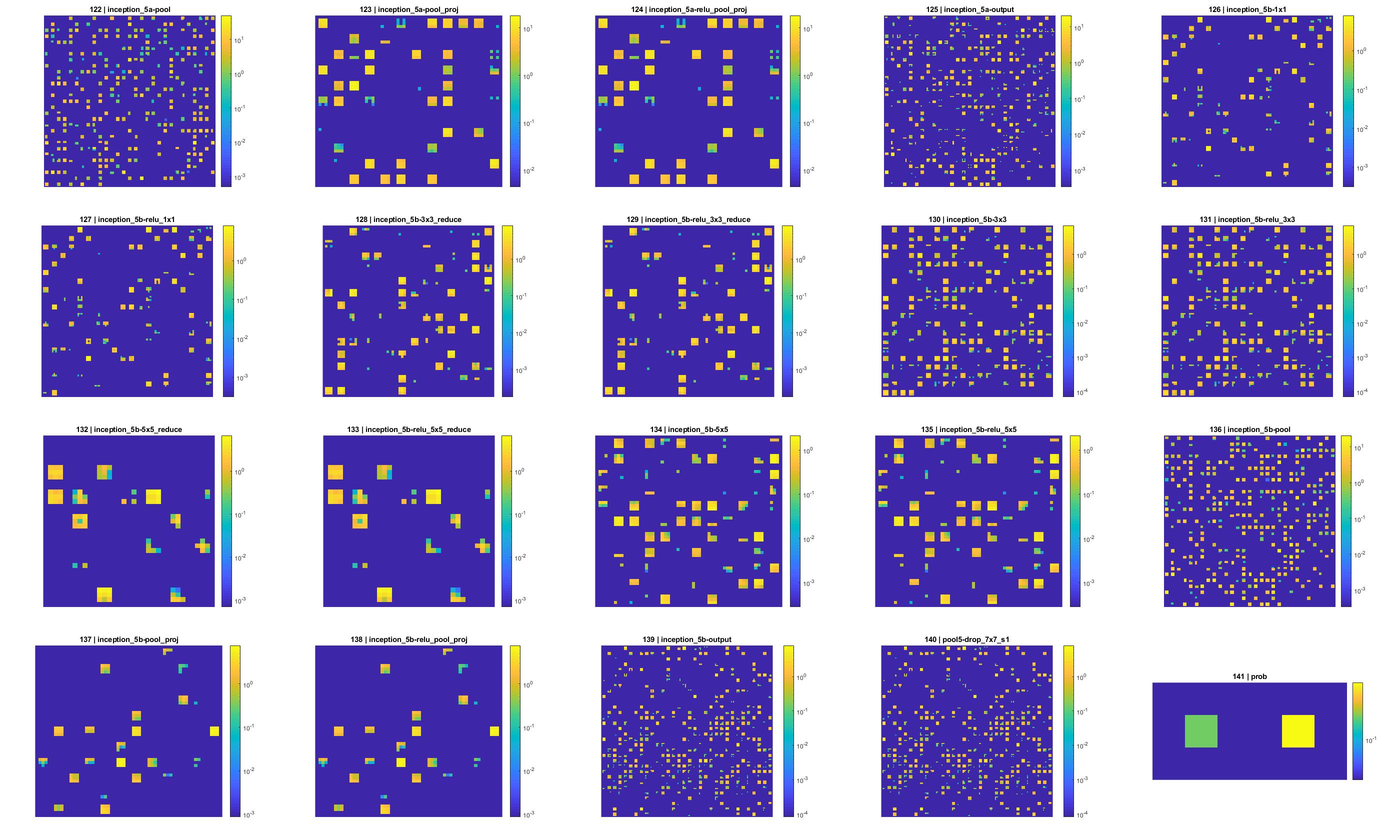


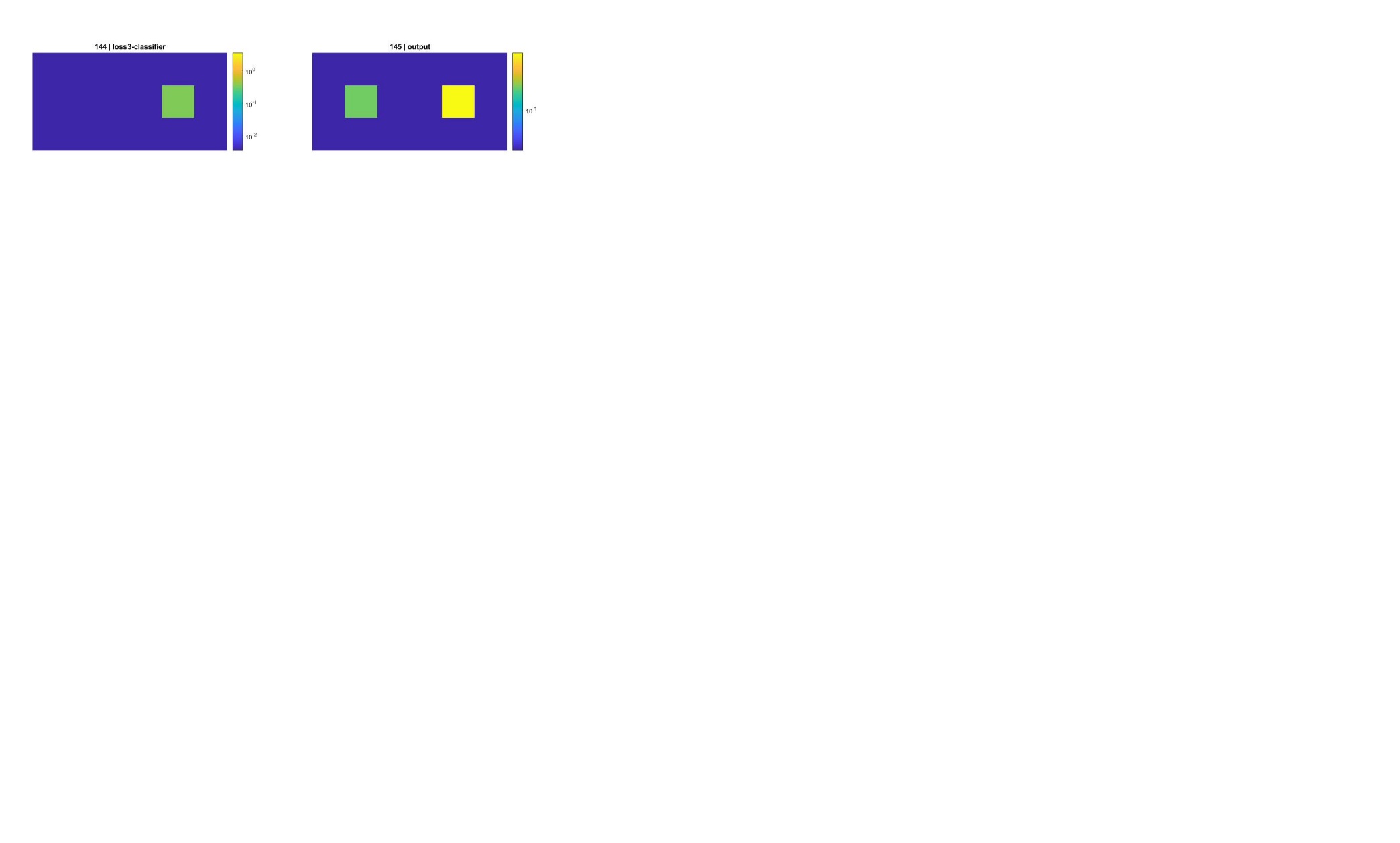


## Supplementary Data 13. TrackNet examples

Following images show trajectories of 50 cells in six field of view over 900 min imaging.


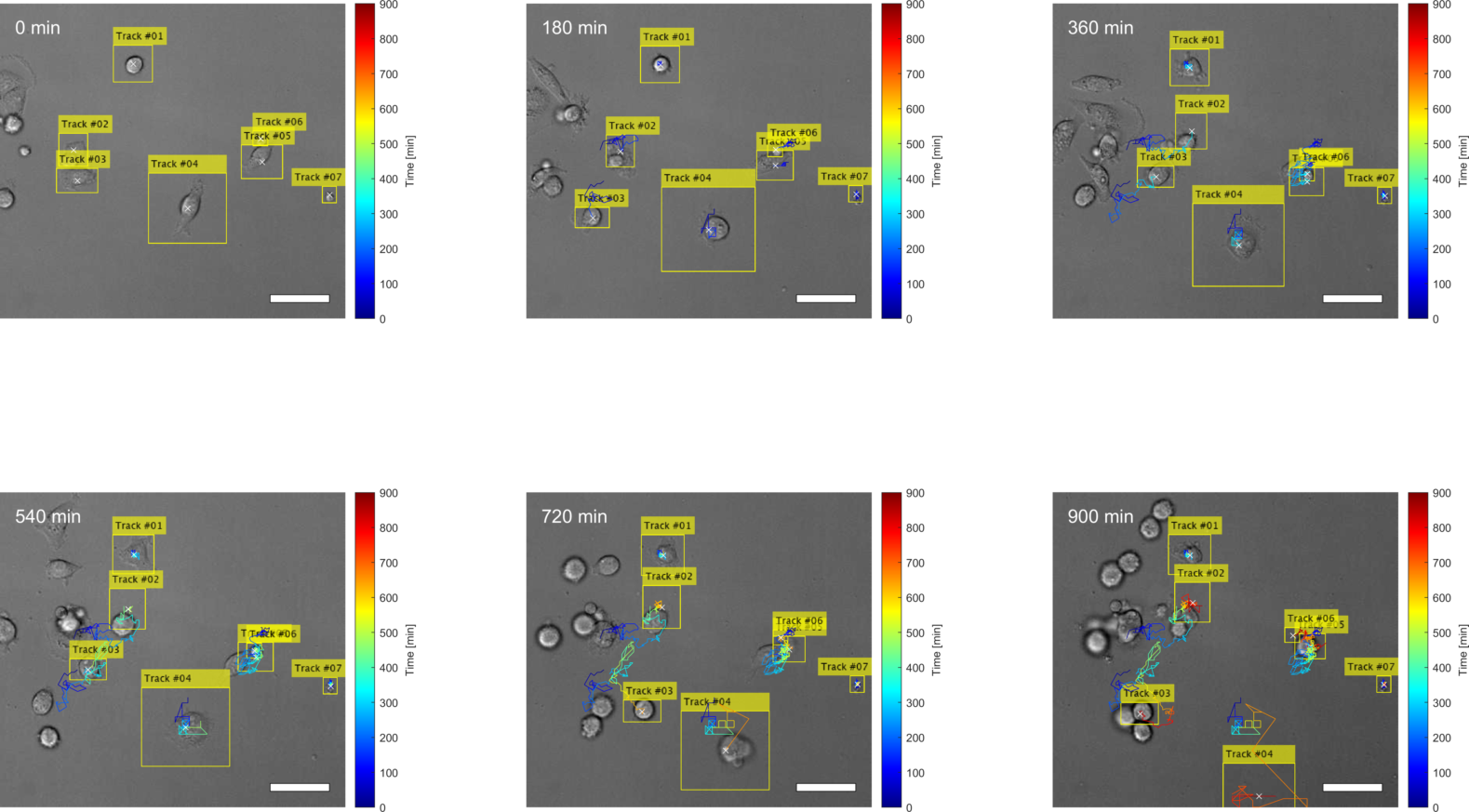


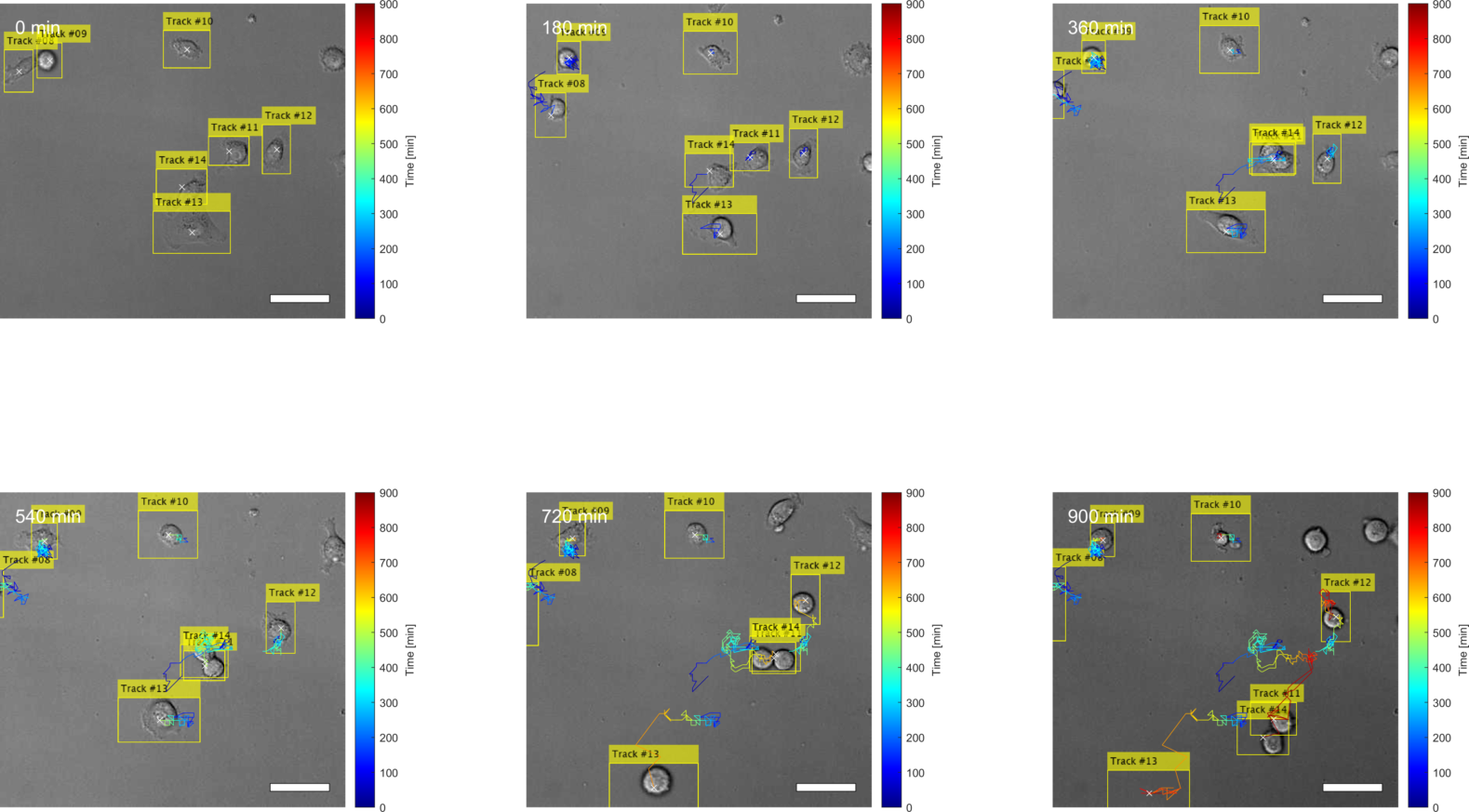


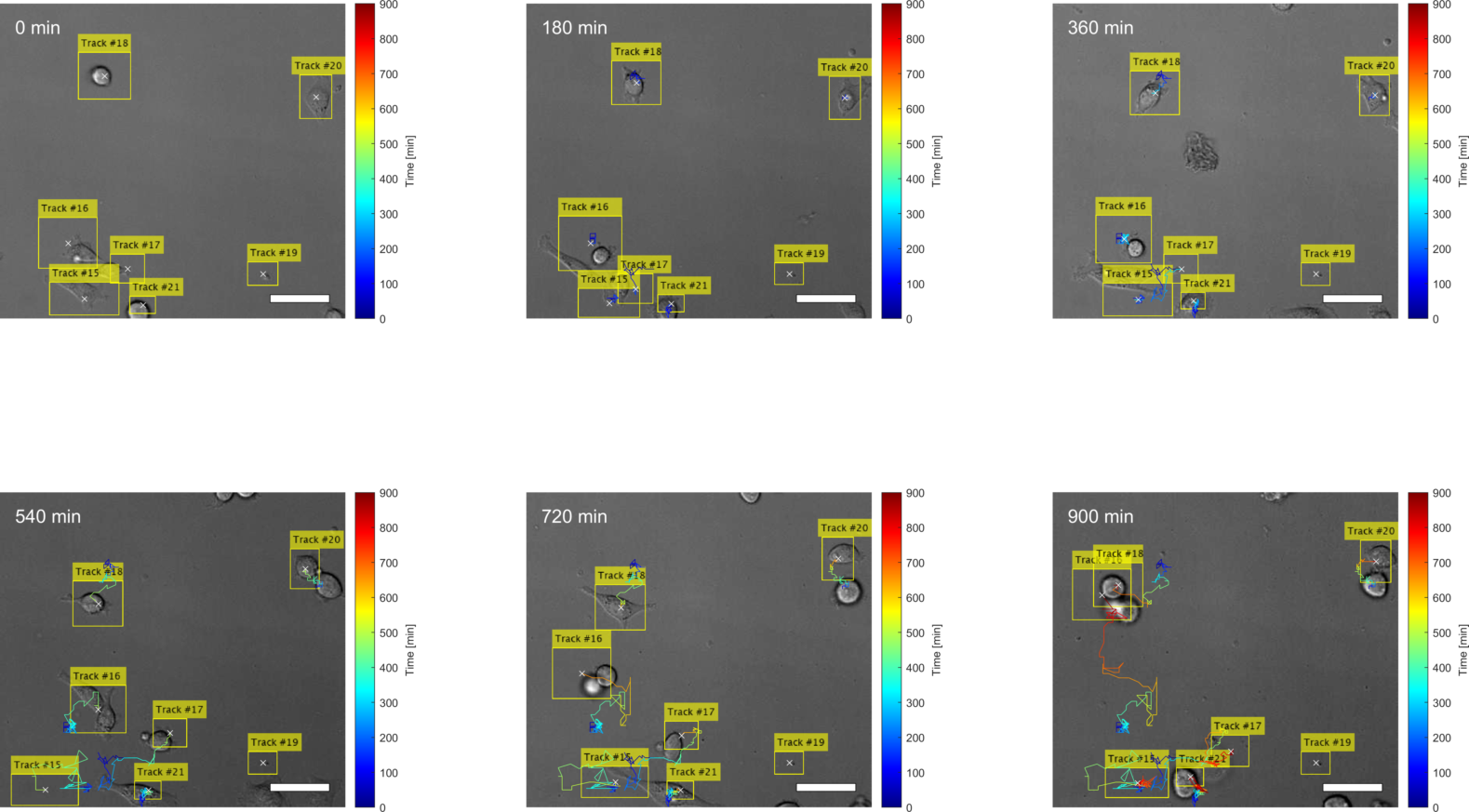


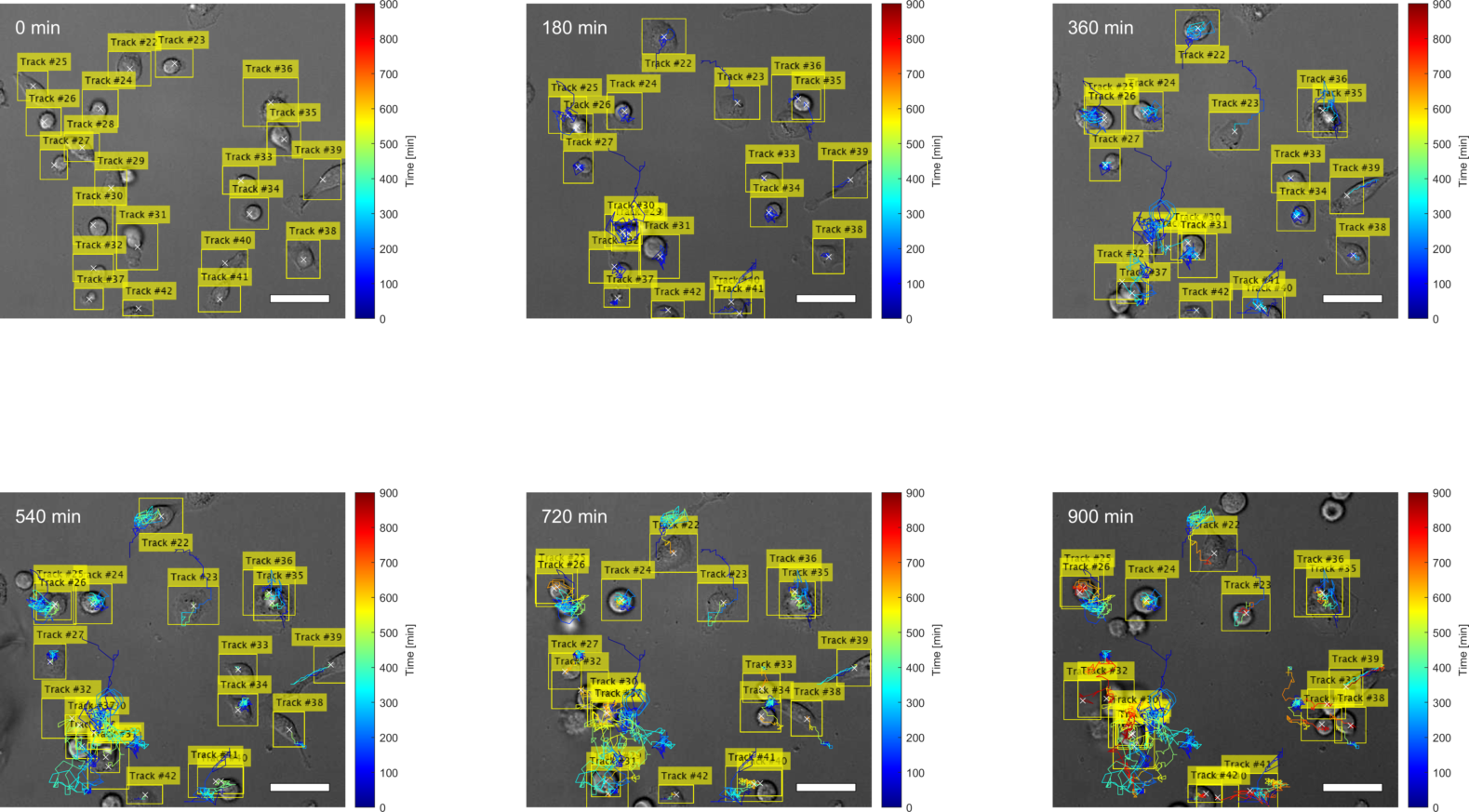


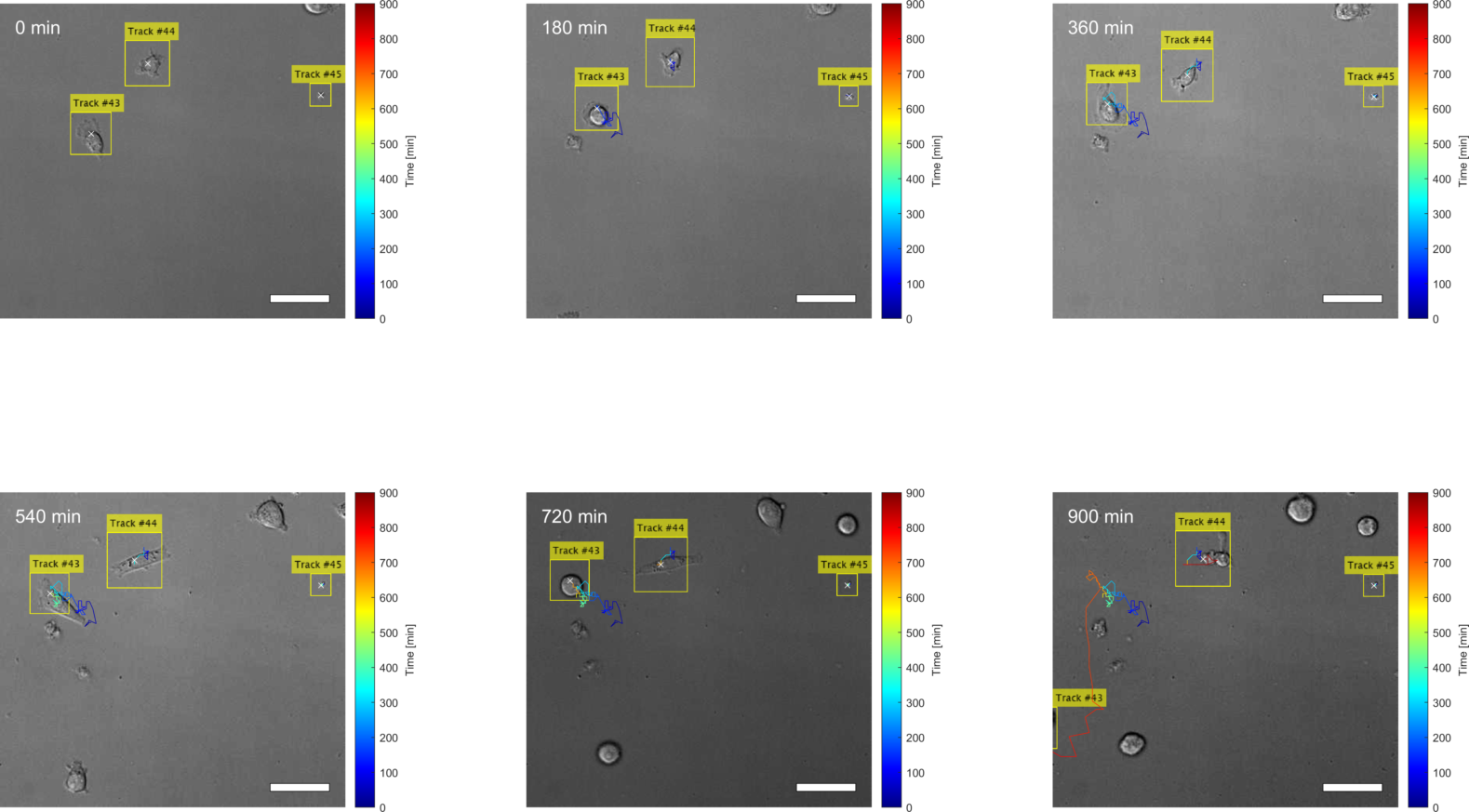


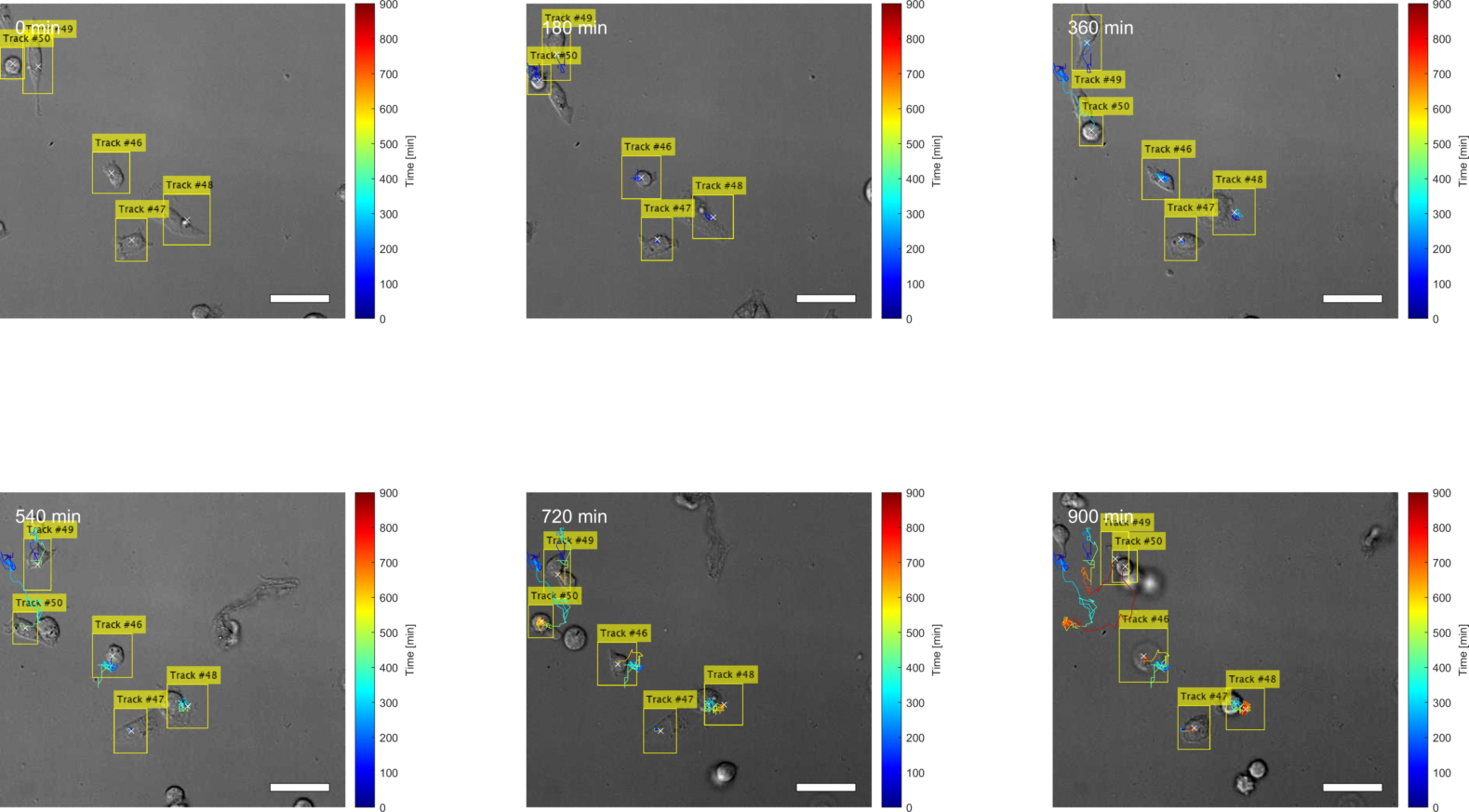


## Supplementary Data 14. AIM examples

Following images show trajectories of ten MDA-MB-231 cells imaging over 900 min. Phototoxicity was introduced by illuminating UV light for first five samples.


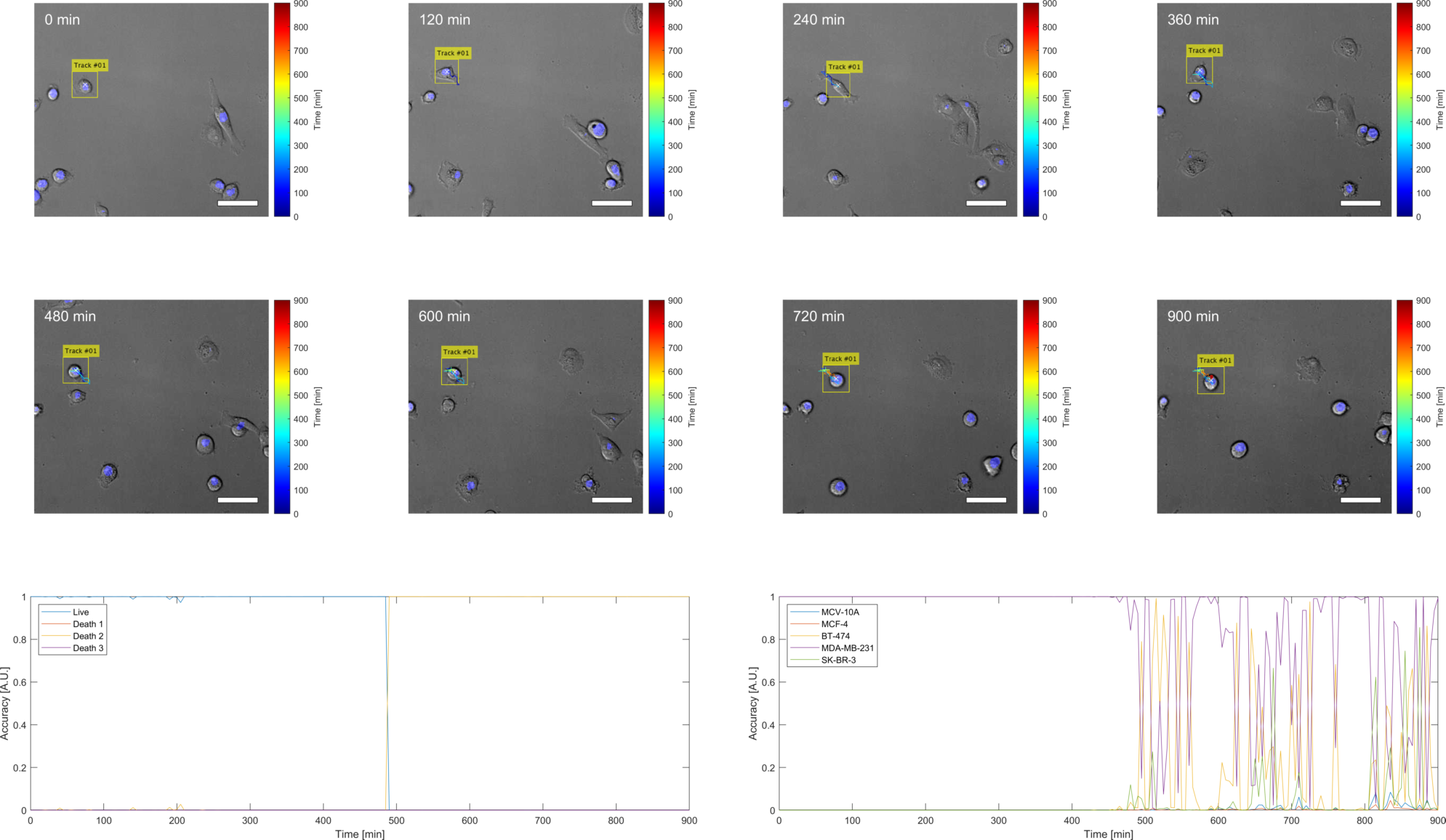


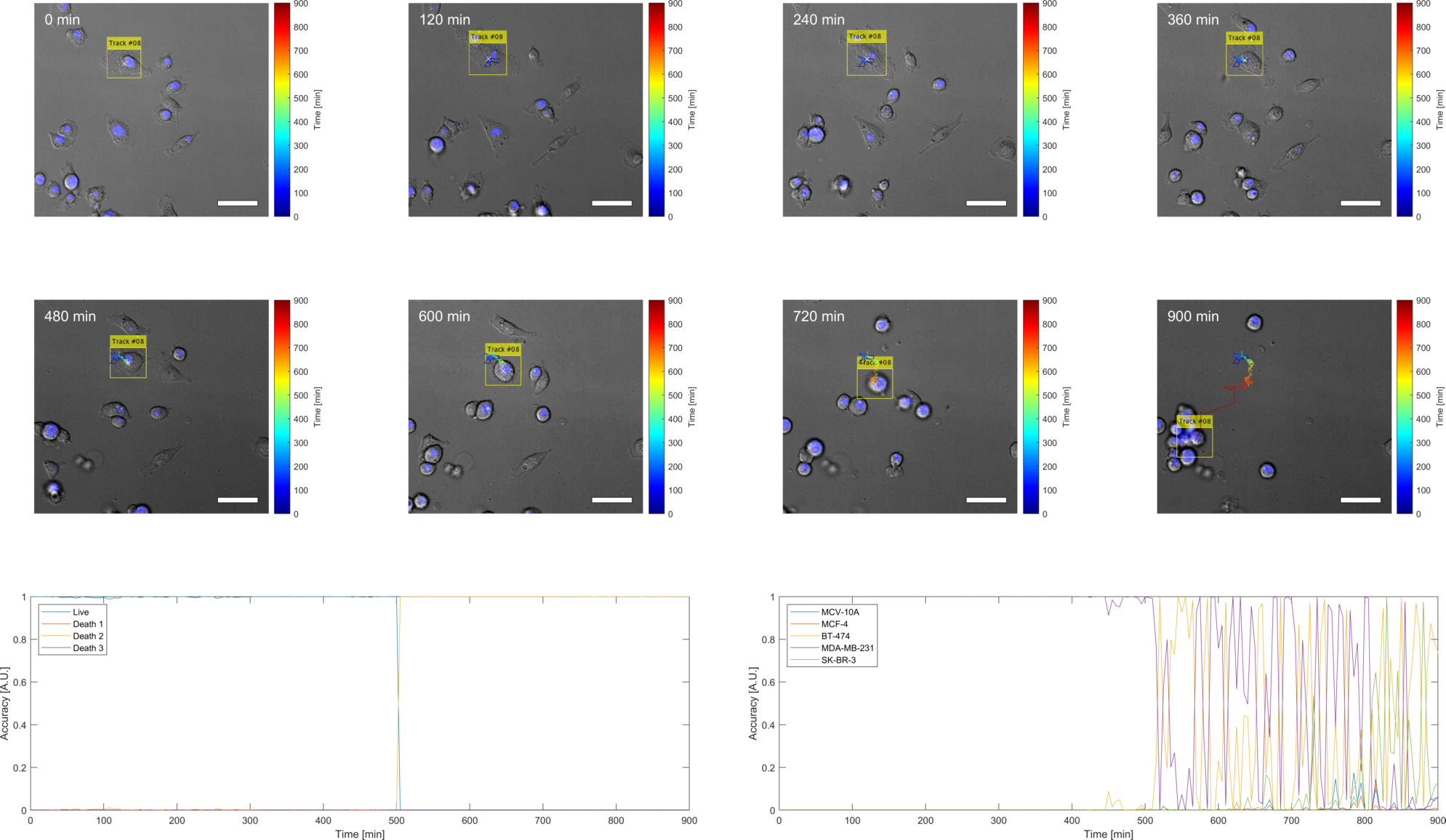


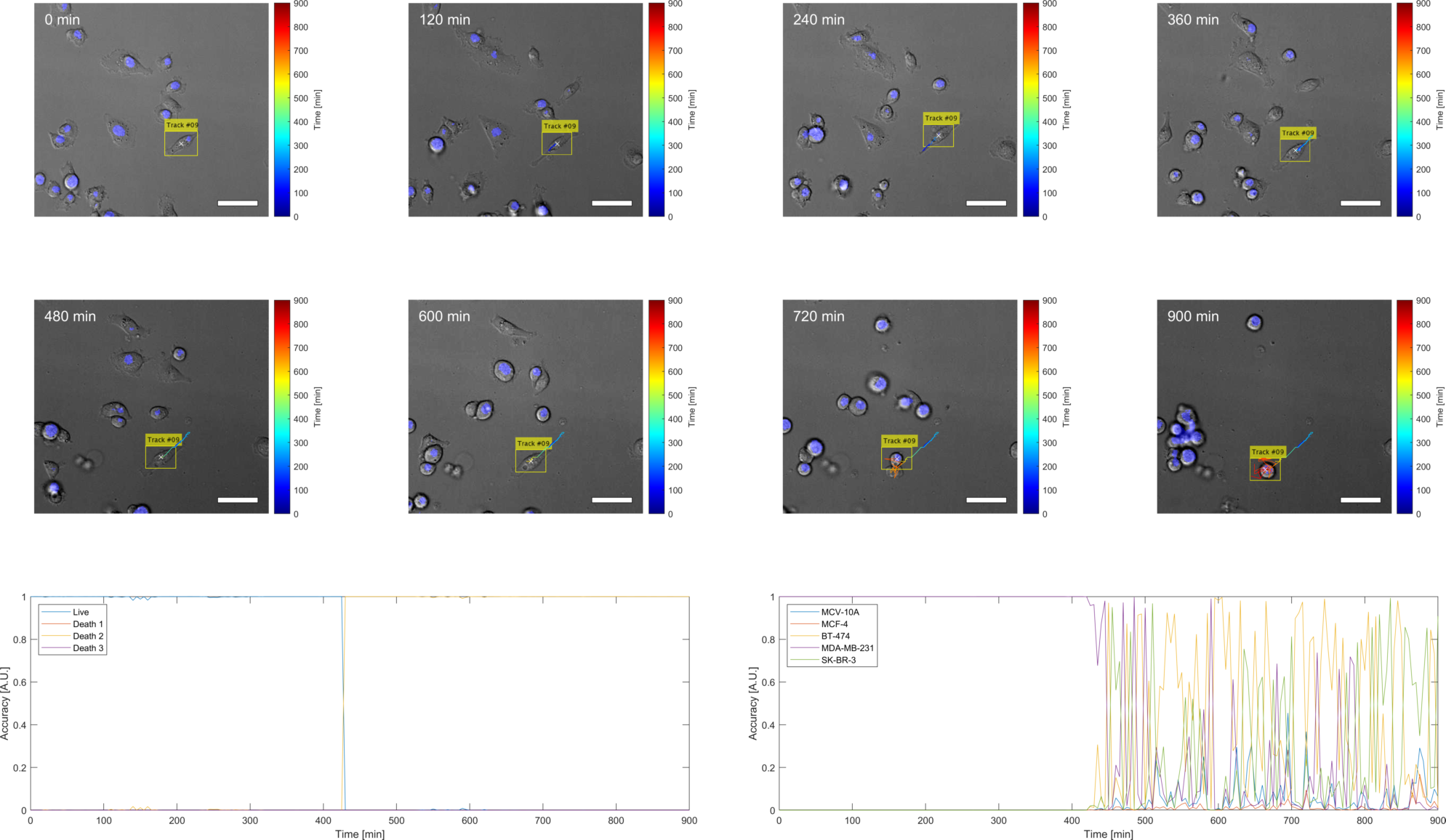


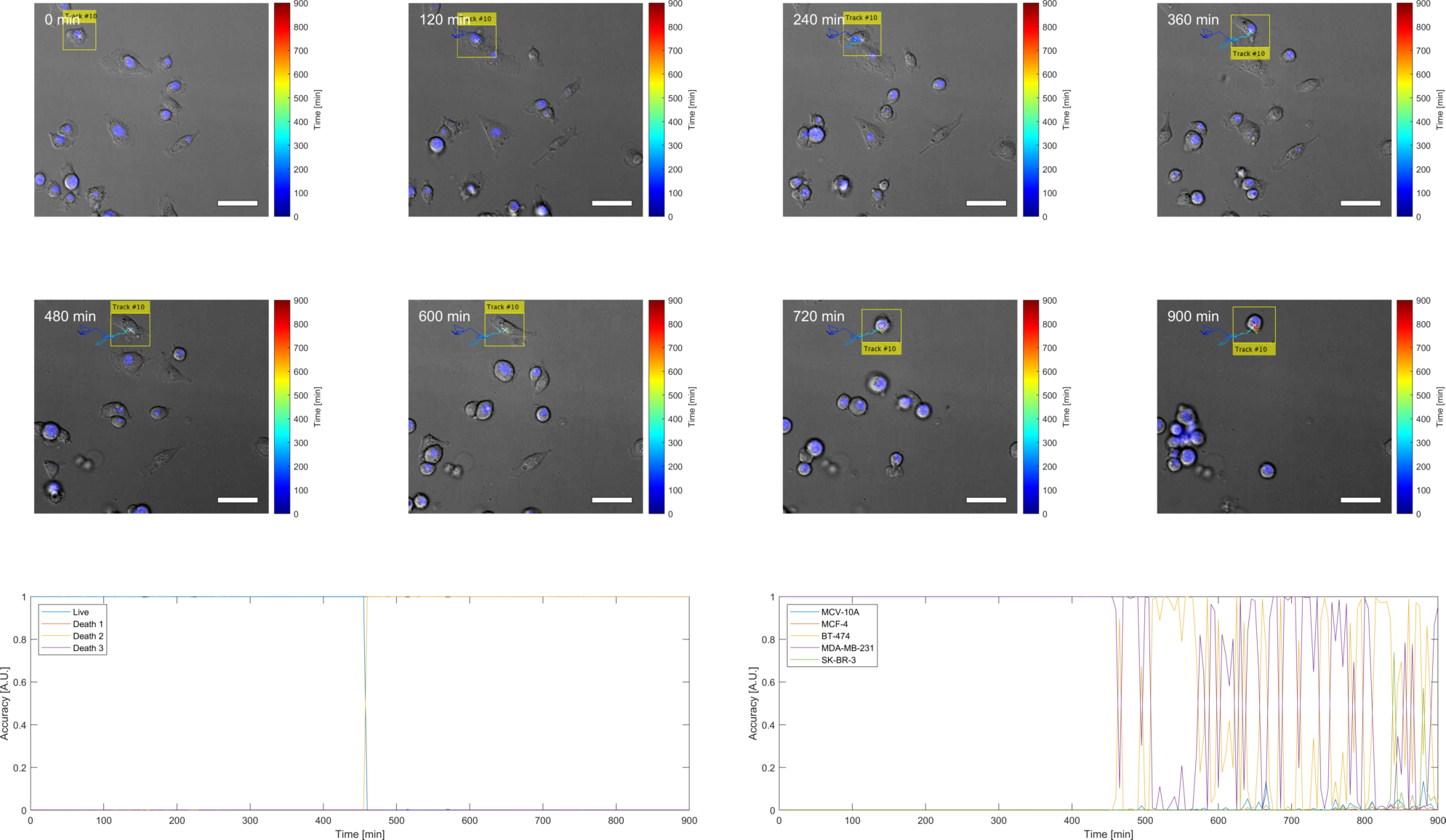

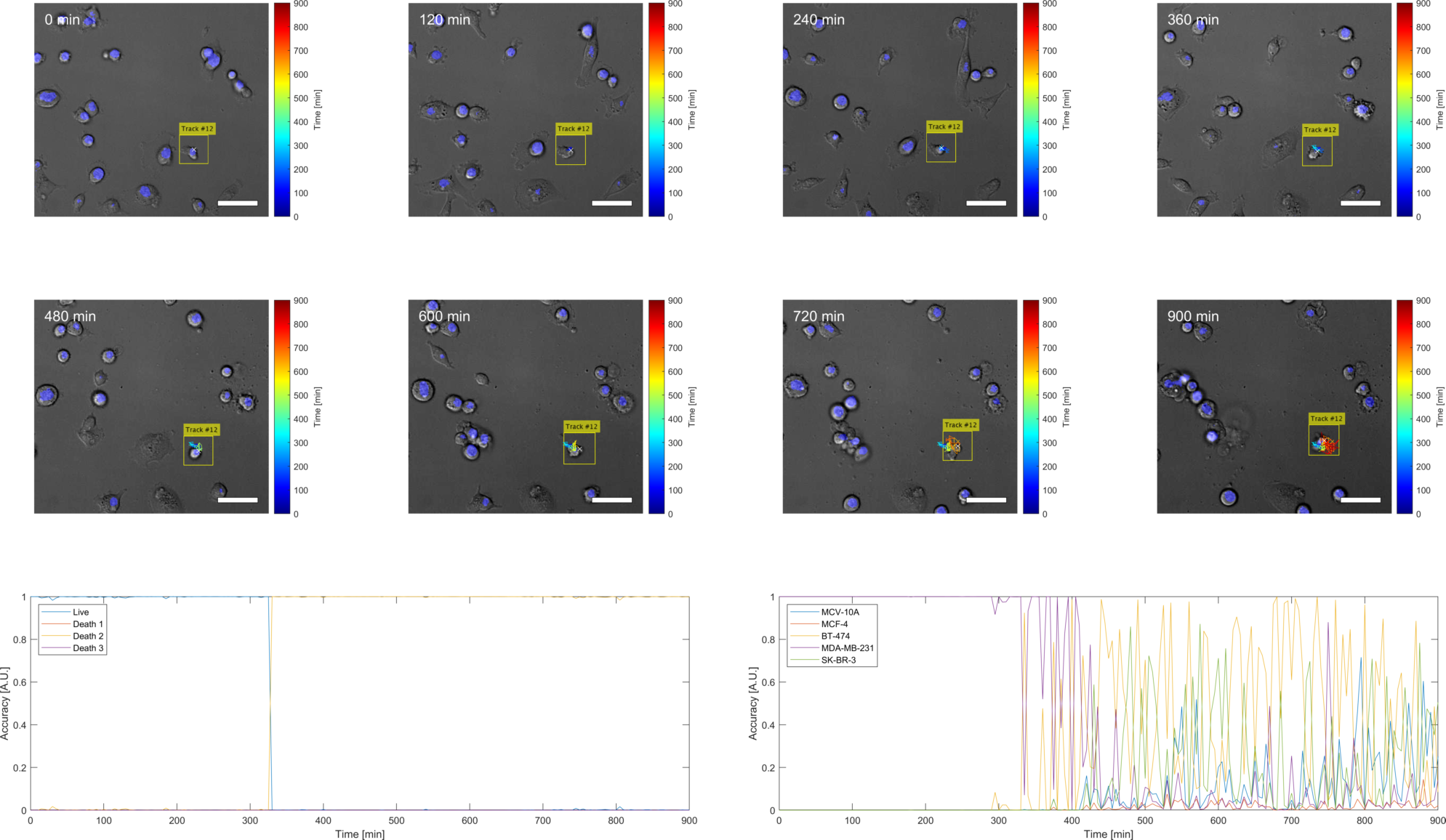


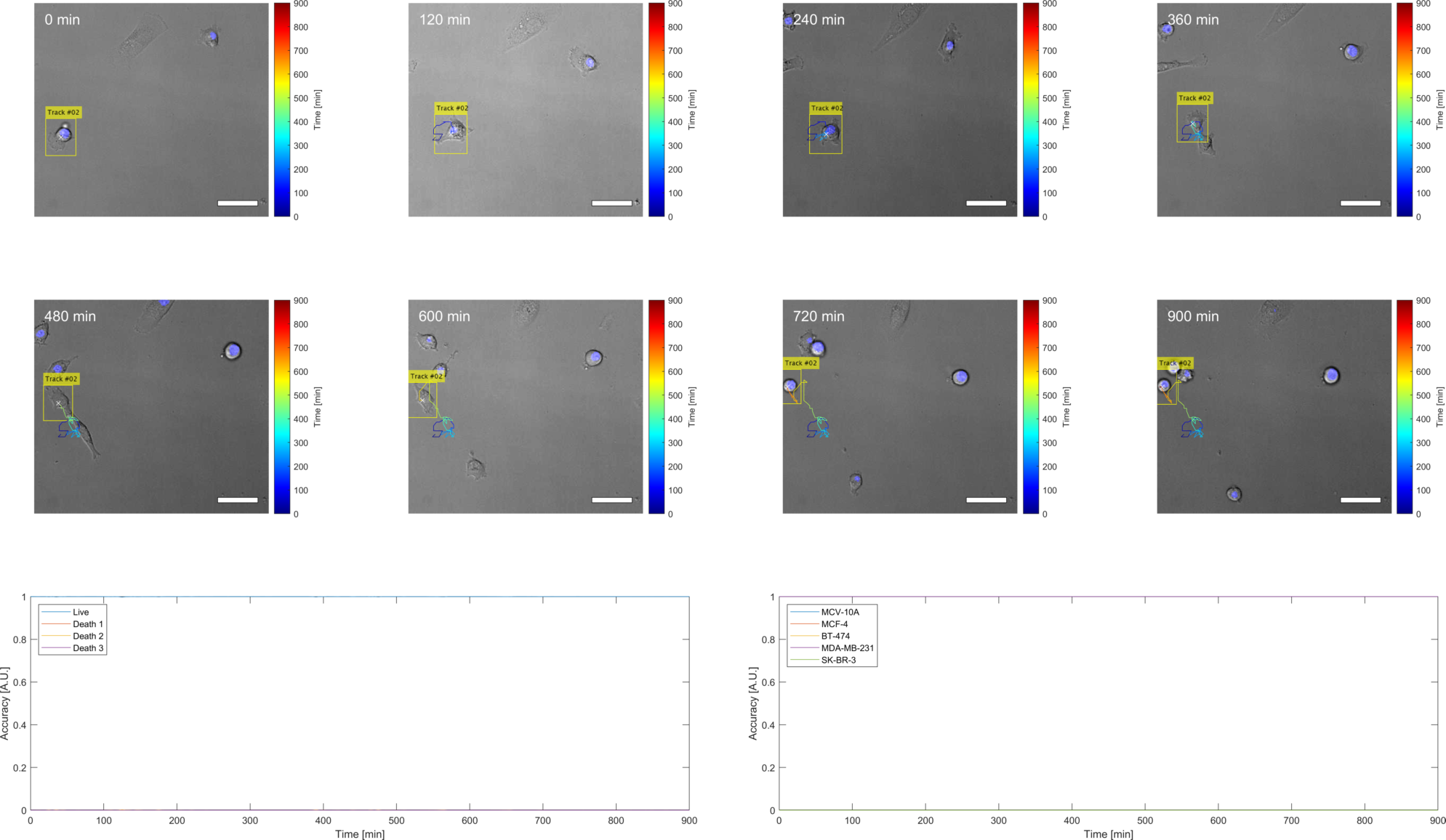


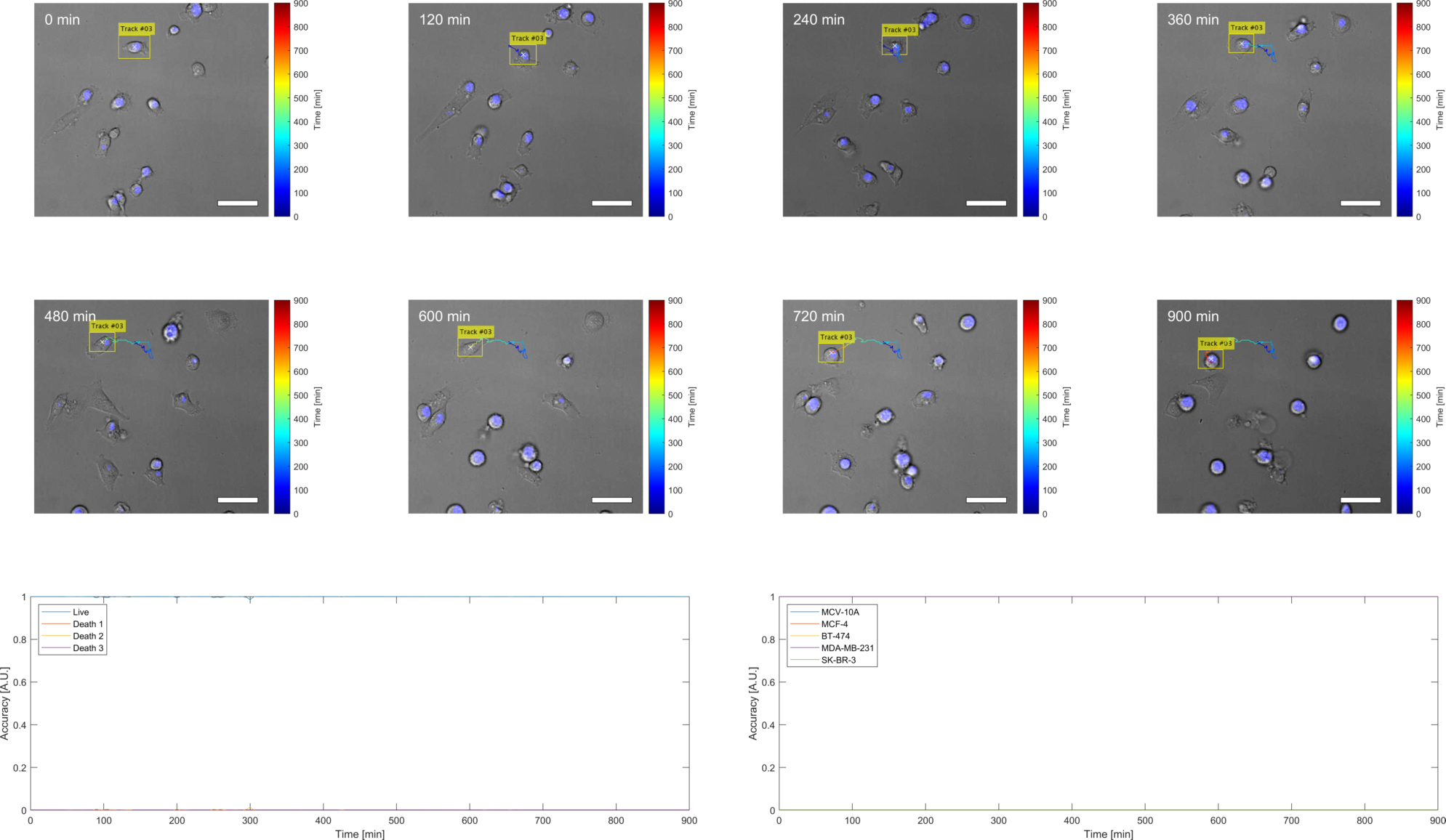


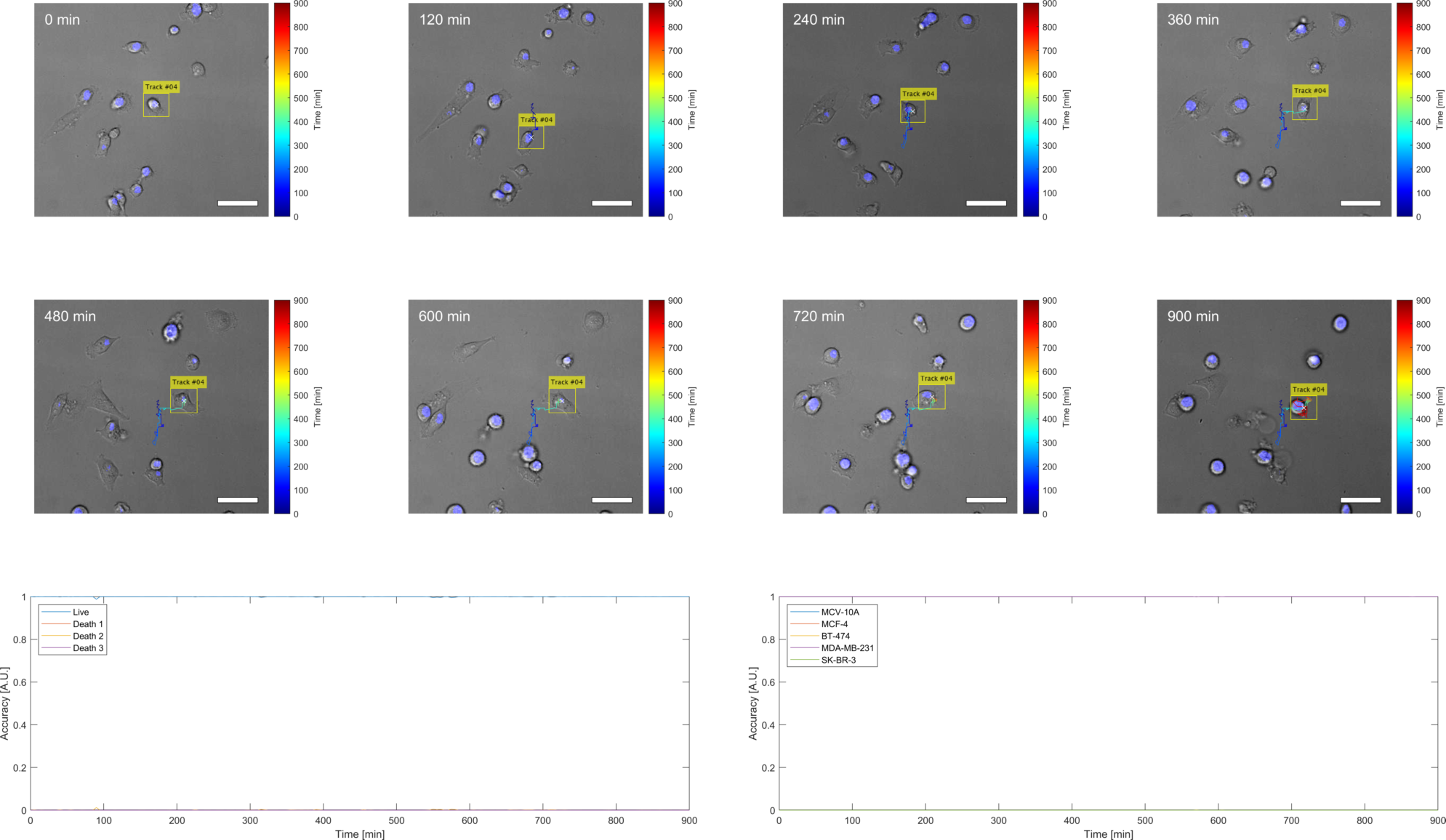


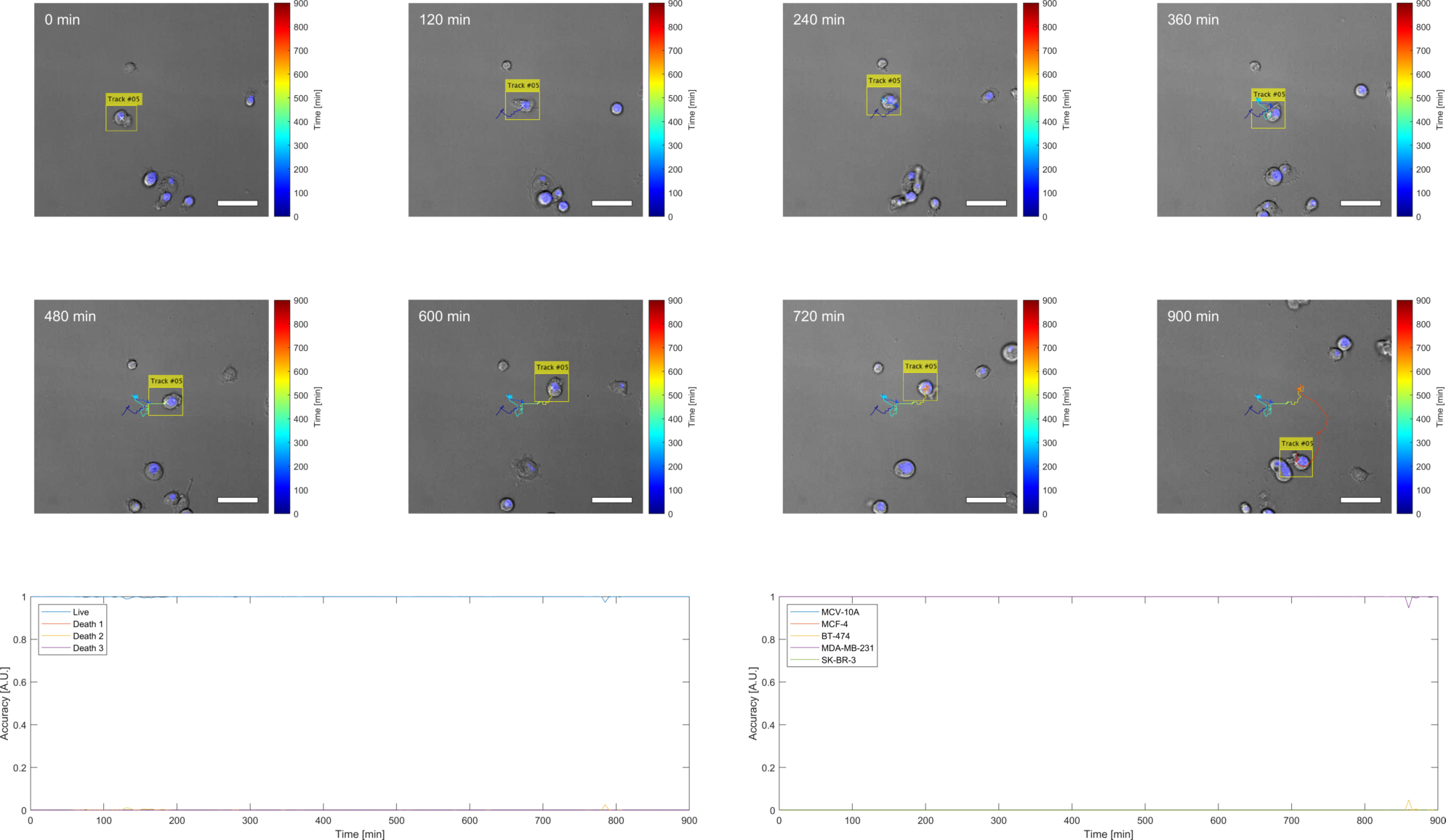


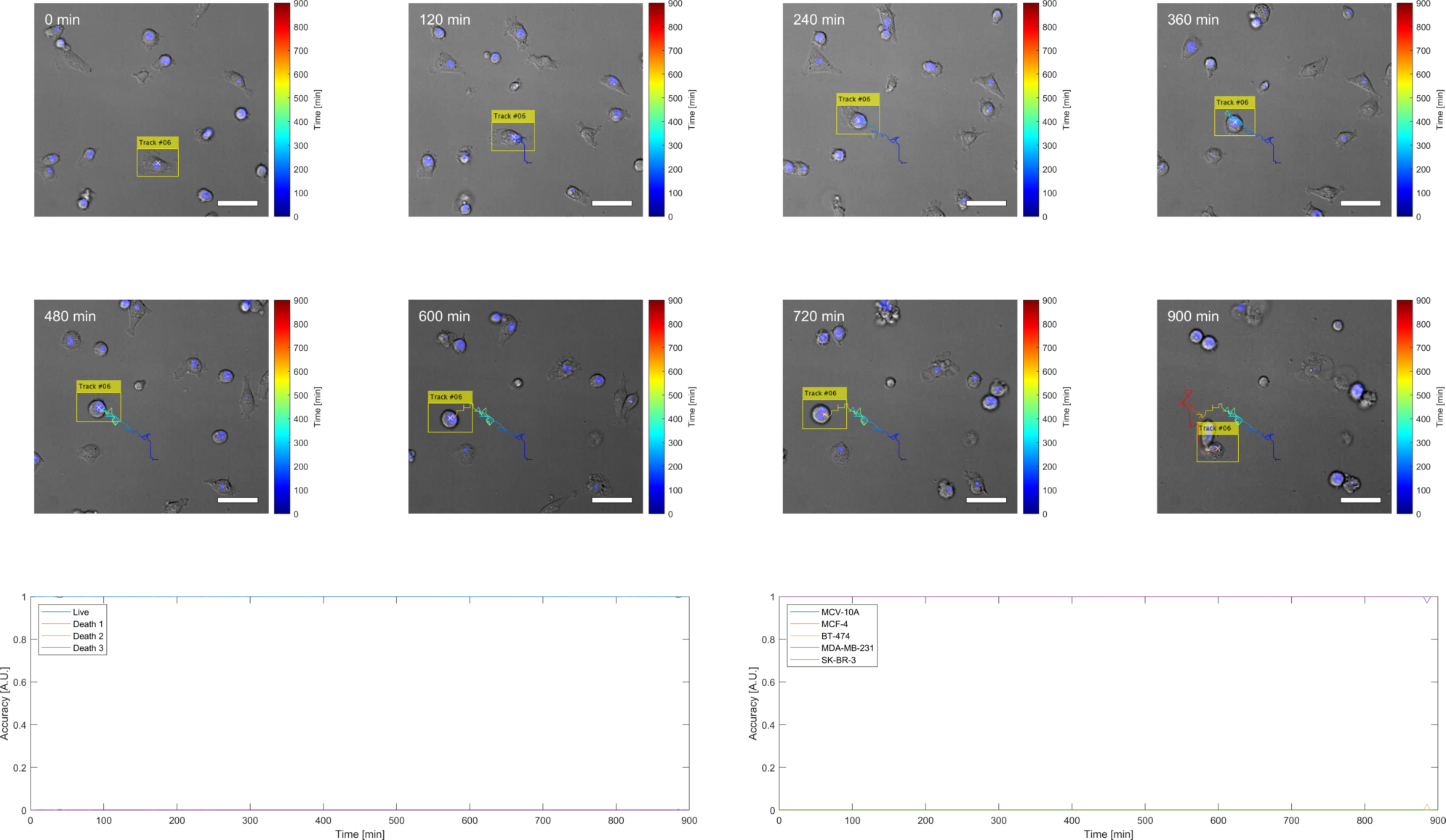


# References

1. Pearson, H. The good, the bad and the ugly. *Nature* **447**, 138–140 (2007).

2. Stewart, M. P. *et al.* In vitro and ex vivo strategies for intracellular delivery. *Nature* **538**, 183–192 (2016).

3. Hamm, A., Krott, N., Breibach, I., Blindt, R. & Bosserhoff, A. K. Efficient transfection method for primary cells. *Tissue Eng.* **8**, 235–245 (2002).

4. Banchereau, J. & Steinman, R. M. Dendritic cells and the control of immunity. *Nature* **392**, 245–252 (1998).

5. Robertson, S., Azizpour, H., Smith, K. & Hartman, J. Digital image analysis in breast pathology—from image processing techniques to artificial intelligence. *Transl. Res.* **194**, 19–35 (2018).

6. Mayerich, D. *et al.* Stain-less staining for computed histopathology. *TECHNOLOGY* **03**, 27–31 (2015).

7. Bychkov, D. *et al.* Deep learning based tissue analysis predicts outcome in colorectal cancer. *Sci. Rep.* **8**, 3395 (2018).

8. Litjens, G. *et al.* Deep learning as a tool for increased accuracy and efficiency of histopathological diagnosis. *Sci. Rep.* **6**, 26286 (2016).

9. Roux, L. *et al.* Mitosis detection in breast cancer histological images An ICPR 2012 contest. *J. Pathol. Inform.* **4**, 8 (2013).

10. Sirinukunwattana, K. *et al.* Locality sensitive deep learning for detection and classification of nuclei in routine colon cancer histology images. *IEEE Trans. Med. Imaging* **35**, 1196–1206 (2016).

11. Cireşan, D. C., Giusti, A., Gambardella, L. M. & Schmidhuber, J. Mitosis detection in breast cancer histology images with deep neural networks. in 411–418 (2013). doi:10.1007/978-3-642-40763-5_51

12. Esteva, A. *et al.* Dermatologist-level classification of skin cancer with deep neural networks. *Nature* **542**, 115–118 (2017).

13. Litjens, G. *et al.* A survey on deep learning in medical image analysis. *Med. Image Anal.* **42**, 60–88 (2017).

14. Moen, E. *et al.* Deep learning for cellular image analysis. *Nat. Methods* (2019).

15. Nyberg, K. D. *et al.* Predicting cancer cell invasion by single-cell physical phenotyping. *Integr. Biol.* **10**, 218–231 (2018).

16. Rostam, H. M., Reynolds, P. M., Alexander, M. R., Gadegaard, N. & Ghaemmaghami, A. M. Image based Machine Learning for identification of macrophage subsets. *Sci. Rep.* **7**, 3521 (2017).

17. Chen, C. L. *et al.* Deep learning in label-free cell classification. *Sci. Rep.* **6**, 21471 (2016).

18. Christiansen, E. M. *et al.* In silico labeling: predicting fluorescent labels in unlabeled images. *Cell* **173**, 792-803.e19 (2018).

19. Ounkomol, C., Seshamani, S., Maleckar, M. M., Collman, F. & Johnson, G. R. Label-free prediction of three-dimensional fluorescence images from transmitted-light microscopy. *Nat. Methods* (2018). doi:10.1038/s41592-018-0111-2

20. De Fauw, J. *et al.* Clinically applicable deep learning for diagnosis and referral in retinal disease. *Nat. Med.* **24**, 1342–1350 (2018).

21. Sadanandan, S. K., Ranefall, P., Le Guyader, S. & Wählby, C. Automated training of deep convolutional neural networks for cell segmentation. *Sci. Rep.* **7**, 7860 (2017).

22. Van Valen, D. A. *et al.* Deep learning automates the quantitative analysis of individual cells in live-cell imaging experiments. *PLOS Comput. Biol.* **12**, e1005177 (2016).

23. Hornik, K., Stinchcombe, M. & White, H. Multilayer feedforward networks are universal approximators. *Neural Networks* **2**, 359–366 (1989).

24. Li Fe-Fei, Fergus & Perona. A Bayesian approach to unsupervised one-shot learning of object categories. *Proc IEEE Int Conf Comput Vis* **2**, 1134–1141 (2003).

25. Pan, S. J. & Yang, Q. A Survey on Transfer Learning. *IEEE Trans. Knowl. Data Eng.* **22**, 1345–1359 (2010).

26. Bertinetto, L., Valmadre, J., Golodetz, S., Miksik, O. & Torr, P. H. S. Staple: complementary learners for real-time tracking. *CVPR* **38**, 1401–1409 (2016).

27. Steinhaus, H. Sur la division des corps matériels en parties. *Bull. Acad. Pol. Sci., Cl. III* **4**, 801–804 (1957).

28. Tucker, A. B. Computer science handbook. *Taylor Fr.* (2004).

29. Shelhamer, E., Long, J. & Darrell, T. Fully convolutional networks for semantic segmentation. *IEEE Trans. Pattern Anal. Mach. Intell.* **39**, 640–651 (2017).

30. Badrinarayanan, V., Kendall, A. & Cipolla, R. Segnet: a deep convolutional encoder-decoder architecture for image segmentation. *IEEE Trans. Pattern Anal. Mach. Intell.* **39**, 2481–2495 (2017).

31. Ronneberger, O., Fischer, P. & Brox, T. U-Net: convolutional networks for biomedical image segmentation. *MICCAI* **9351**, 234–241 (2015).

32. Noh, H., Hong, S. & Han, B. Learning deconvolution network for semantic segmentation. *ICCV* **2015**, 1520–1528 (2015).

33. Pohlen, T., Hermans, A., Mathias, M. & Leibe, B. Full-resolution residual networks for semantic segmentation in street scenes. *CVPR* **2017**, 3309–3318 (2017).

34. Rumelhart, D. E., Hinton, G. E. & Williams, R. J. Learning representations by back-propagating errors. *Nature* **323**, 533–536 (1986).

35. Kingma, D. P. & Ba, J. Adam: a method for stochastic optimization. *ICLR* **2014**, 1–15 (2014).

36. Dima, A. A. *et al.* Comparison of segmentation algorithms for fluorescence microscopy images of cells. *Cytom. Part A* **79A**, 545–559 (2011).

37. Du, X. Segmentation of Fluorescence Microscopy Cell Images Using Unsupervised Mining. *Open Med. Inform. J.* **4**, 41–49 (2010).

38. Mukamel, E. A., Nimmerjahn, A. & Schnitzer, M. J. Automated Analysis of Cellular Signals from Large-Scale Calcium Imaging Data. *Neuron* **63**, 747–760 (2009).

39. Yang, W. & Liu, J. Denoising fluorescence molecular image by K-Means clustering. in *2017 3rd IEEE International Conference on Computer and Communications (ICCC)* 1847–1850 (IEEE, 2017). doi:10.1109/CompComm.2017.8322858

40. Dufour, A., Meas-Yedid, V., Grassart, A. & Olivo-Marin, J.-C. Automated quantification of cell endocytosis using active contours and wavelets. in *2008 19th International Conference on Pattern Recognition* 1–4 (IEEE, 2008). doi:10.1109/ICPR.2008.4761748

41. Ren, S., He, K., Girshick, R. & Sun, J. Faster R-CNN: towards real-time object detection with region proposal networks. *IEEE Trans. Pattern Anal. Mach. Intell.* **39**, 1137–1149 (2017).

42. Krizhevsky, A., Sutskever, I. & Hinton, G. E. ImageNet classification with deep convolutional neural networks. *Adv. Neural Inf. Process. Syst.* 1–9 (2012).

43. Szegedy, C. *et al.* Going deeper with convolutions. *CVPR* **2015**, 1–9 (2015).

44. Szegedy, C., Vanhoucke, V., Ioffe, S., Shlens, J. & Wojna, Z. Rethinking the inception architecture for computer vision. *CVPR* **2016**, 2818–2826 (2016).

45. Szegedy, C., Ioffe, S., Vanhoucke, V. & Alemi, A. Inception-v4, Inception-ResNet and the impact of residual connections on learning. *Pattern Recognit. Lett.* **42**, 11–24 (2016).

46. Ulman, V. *et al.* An objective comparison of cell-tracking algorithms. *Nat. Methods* **14**, 1141–1152 (2017).

47. Moser, B., Hochreiter, B., Herbst, R. & Schmid, J. A. Fluorescence colocalization microscopy analysis can be improved by combining object-recognition with pixel-intensity-correlation. *Biotechnol. J.* **12**, 1600332 (2017).

48. Dalod, M., Chelbi, R., Malissen, B. & Lawrence, T. Dendritic cell maturation: functional specialization through signaling specificity and transcriptional programming. *EMBO J.* **33**, 1104–16 (2014).

49. Ren, S., He, K., Girshick, R. & Sun, J. Faster R-CNN: towards real-time object detection with region proposal networks. *IEEE Trans. Pattern Anal. Mach. Intell.* **39**, 1137–1149 (2015).
